# The marine diversity spectrum

**DOI:** 10.1111/1365-2656.12194

**Published:** 2014-03-03

**Authors:** Daniel C Reuman, Henrik Gislason, Carolyn Barnes, Frédéric Mélin, Simon Jennings

**Affiliations:** 1 Department of Life Sciences, Imperial College LondonSilwood Park Campus, Ascot, SL5 7PY, UK; 2 Laboratory of Populations, Rockefeller University1230 York Ave, New York, NY, 10065, USA; 3 Technical University of DenmarkCharlottenlund Slot, DK-2920, Charlottenlund, Denmark; 4 Centre for Environment, Fisheries and Aquaculture ScienceLowestoft, Suffolk, NR33 OHT, UK; 5 European Commission, Joint Research Centre, Institute for Environment and Sustainability21027, Ispra (VA), Italy; 6 School of Environmental Sciences, University of East AngliaNorwich, NR4 7TJ, UK

**Keywords:** biodiversity, body mass, community, neutral theory, power law, size spectrum

## Abstract

1. Distributions of species body sizes within a taxonomic group, for example, mammals, are widely studied and important because they help illuminate the evolutionary processes that produced these distributions. Distributions of the sizes of species within an assemblage delineated by geography instead of taxonomy (all the species in a region regardless of clade) are much less studied but are equally important and will illuminate a different set of ecological and evolutionary processes.
2. We develop and test a mechanistic model of how diversity varies with body mass in marine ecosystems. The model predicts the form of the ‘diversity spectrum’, which quantifies the distribution of species' asymptotic body masses, is a species analogue of the classic size spectrum of individuals, and which we have found to be a new and widely applicable description of diversity patterns.
3. The marine diversity spectrum is predicted to be approximately linear across an asymptotic mass range spanning seven orders of magnitude. Slope −0·5 is predicted for the global marine diversity spectrum for all combined pelagic zones of continental shelf seas, and slopes for large regions are predicted to lie between −0·5 and −0·1. Slopes of −0·5 and −0·1 represent markedly different communities: a slope of −0·5 depicts a 10-fold reduction in diversity for every 100-fold increase in asymptotic mass; a slope of −0·1 depicts a 1·6-fold reduction. Steeper slopes are predicted for larger or colder regions, meaning fewer large species per small species for such regions.
4. Predictions were largely validated by a global empirical analysis.
5. Results explain for the first time a new and widespread phenomenon of biodiversity. Results have implications for estimating numbers of species of small asymptotic mass, where taxonomic inventories are far from complete. Results show that the relationship between diversity and body mass can be explained from the dependence of predation behaviour, dispersal, and life history on body mass, and a neutral assumption about speciation and extinction.

## Introduction

Most species are small. The nature of this bias and its causes and ramifications have been a focus of ecological and evolutionary research for decades (e.g. Hutchinson & MacArthur, 1959; Van Valen 1973; May 1978; Dial & Marzluff, 1988; Maurer & Brown, 1988; Blackburn & Gaston, 1994; Loder, Blackburn & Gaston, 1997; Purvis, Orme & Dolphin, 2003; Marquet *et al.* 2005; Clauset & Erwin, 2008). As well as illuminating ecological and evolutionary processes, body mass–diversity relationships are important for conservation because they help quantify existing diversity. Most past work has considered these relationships in species assemblages delineated by taxon (e.g. mammals). We approach the topic from a fundamentally different but equally important perspective: how can body mass-diversity relationships be explained for geographically delineated but taxon-inclusive assemblages, that is, all the species in a region? Different mechanisms will take primacy in this new context. For instance, while patterns in taxon-specific global assemblages will be strongly affected by evolutionary history and physiological constraints of the taxon, patterns in geographically constrained assemblages will be more affected by community assembly. We here offer a first empirical description and explanatory model of mass-diversity patterns in an important class of geographically constrained assemblages: those in the world's continental shelf seas.

We consider a community consisting of individuals in any specified focal region in the world's continental shelf seas and with asymptotic mass in any specified focal range. Diversity of the community is influenced by four main quantities that form the main structural components of our model: (1) the number of individuals in the community; (2) the number of individuals in a much larger ‘metacommunity’ that is outside the focal region but that is delimited by the same asymptotic mass range; (3) commonness of dispersal between the community and metacommunity; and (4) a speciation rate. These determinants of diversity are well known from the theory of island biogeography (MacArthur & Wilson, 2001) and the neutral theory of biodiversity (Hubbell 2001). By unifying and extending life history and size spectrum theory from several sources (e.g. Ware 1978; West, Brown & Enquist, 2001; Andersen & Beyer, 2006), our model makes predictions from first principles for how these four quantities depend on the asymptotic mass bounds used and the environment in the focal region. The model then combines the results using formulas from neutral theory (Etienne & Olff, 2004) to predict community diversity. Diversity refers to numbers of species throughout.

Our model answers several specific questions. What is the diversity in the focal community and how does it depend on the asymptotic mass range used? How is the relationship between diversity and mass affected by the environmental characteristics of the focal region? What are the individual- and population-level mechanisms controlling these patterns?

The main assumption of our model is that organisms of similar asymptotic mass in marine pelagic realms can be approximated to be equivalent competitors – this is the neutral assumption. Our theory applies principally to pelagic environments because the neutral assumption and model parameterizations of life history, predation and dispersal are more likely accurate there. ‘Pelagic’ is used here and throughout to encompass organisms living or interacting primarily in the water column, including bottom-dwelling species which live on or near the sea bed but are not permanently constrained to the substrate. We exclude coral reefs and reef-associated species. Each species is assigned a characteristic body mass (the asymptotic mass) and counted as belonging to the mass category of its characteristic mass. Asymptotic mass is used as the characteristic mass because many aspects of life history and predation and dispersal behaviour of a species are strongly related to asymptotic mass. The approach of using characteristic masses is consistent with prior work, although much past work was based on groups exhibiting determinate growth and therefore used average mass as the characteristic mass.

As a unification of size-based life history and population growth theory with neutral theory within size categories, our model is inspired and influenced by earlier theoretical approaches such as the models of Etienne & Olff (2004), O'Dwyer *et al*. (2009) and Rossberg (2012, 2013). Our model is more targeted to a particular ecosystem type than some of these and is more directly confronted with data. Our model also builds upon and is heavily influenced by the model of Andersen & Beyer (2006). It extends theirs to address questions of diversity through the use of neutral theory. Our model has some similar features to models of Rossberg (2013), but is more focussed on diversity spectra and biogeographical variation in the diversity spectrum. Our approach goes beyond a body of prior statistical work on the biogeography of marine diversity (e.g. Alroy 2010; Barton *et al.* 2010; Beaugrand, Edwards & Legendre, 2010; Tittensor *et al.* 2010), because the focus is on how diversity varies with body size and also because the model is explicitly mechanistic. Our work is part of a broad effort to unify species- and size-based research approaches in community ecology (e.g. Jennings *et al.* 2001; Brose *et al.* 2006; Reuman *et al.* 2008, 2009b; Rossberg 2012, 2013; Trebilco *et al.* 2013). An earlier version of the diversity spectrum, similar to but distinct from that used here, was defined by Rice & Gislason (1996) and Gislason & Rice (1998). The diversity spectrum as used here was defined for the first time by (Reuman *et al.* 2008, 2009b) and was shown there to have consistent properties among ecosystems with systematic variation in parameters.

The empirical and theoretical descriptions of the diversity spectrum we provide are important for several reasons. First, and perhaps most importantly, we found that the diversity spectrum, described systematically here for the first time for marine systems, captures very widespread phenomena of diversity and reflects how abiotic factors influence diversity. It therefore merits empirical and mechanistic theoretical description. Secondly, data and theory about the diversity spectrum are useful for estimating numbers of species in small mass categories, where taxonomic inventories are far from complete. We provide such estimates for all continental shelf-sea regions in aggregate and for specific regions. Estimates such as these, as well as the general form and systematic variation in the diversity spectrum that we describe, may be useful for establishing baselines in conservation and monitoring efforts, including planning aimed at marine reserve design. Finally, by formulating a mechanistic model, this study tests the hypothesis that well-known patterns of life history, predation and dispersal of marine organisms combined with a neutral null assumption for speciation and extinction can explain patterns of marine diversity. Our model is a useful approximating model that illuminates the main mechanisms behind a new set of important global diversity phenomena.

## Model formulation

### Preliminaries: spectra and distributions

**Fig 1:**
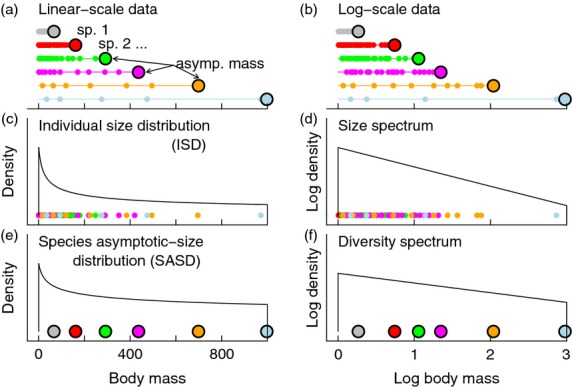
Schematic illustration of basic definitions of spectra and distributions. Each species occurring in a region has an asymptotic mass (large dots), and the individuals of that species have masses less than or equal to the asymptotic mass (small dots, linear scale, (a); separate data on the log scale, (b)). Individuals of a species are all growing towards the species asymptotic mass, indicated by the thin coloured lines in (a) and (b). The individual size distribution (ISD; c) describes how the body sizes of all individuals in the region, regardless of species, are distributed. The size spectrum (d) provides equivalent information in different form – it is the log of the distribution of log individual body sizes. The species asymptotic-size distribution (SASD; e) is a species analogue of the ISD, and the diversity spectrum (f) is a species analogue of the size spectrum – these tools indicate how species asymptotic sizes are distributed.

Diversity–body mass relationships can be characterized using the *diversity spectrum* and a mathematically equivalent but superficially different *species asymptotic-size distribution,* defined here along with related concepts (Fig. 1). Let *R* be the focal shelf-sea region, and let *m* denote the body mass of an individual and *m*_∞_ the asymptotic body mass of a species or an individual.

The *individual size distribution* is defined as the probability density function (pdf) of *m* for individuals in *R*, regardless of species (Fig. 1c). The classic *size spectrum* (also called the abundance spectrum; Kerr & Dickie, 2001) is usually obtained by dividing the log(*m*) axis into bins of equal width and plotting against bin centres the log numbers of individuals (again regardless of species) in *R* in each bin (the logarithmic base used here and elsewhere in this section makes no substantive difference). If η = log(*m*), one can alternatively use the equivalent definition that the size spectrum is the log of the pdf of η for the region (Fig. 1d). We adopt the latter definition because of statistical weaknesses of the bin-based definition (White, Enquist & Green, 2008). The size spectrum is linear if and only if the individual size distribution is a power-law distribution, in which case its slope is 1 plus the exponent of the power law (Andersen & Beyer, 2006; White, Enquist & Green, 2008; Reuman *et al.* 2008; Appendix S2·1).

The *individual asymptotic-size distribution* and the *asymptotic-size spectrum* can be defined in an analogous way to the individual size distribution and size spectrum. The individual asymptotic-size distribution is the pdf of *m*_∞_ for individuals in *R*, regardless of species. The asymptotic-size spectrum can be obtained by dividing the individual log(*m*_∞_) axis into bins of equal width and plotting against bin centres the log numbers of individuals in *R* with *m*_∞_ in each bin. If η_∞_ = log(*m*_∞_), then the statistically preferable but conceptually equivalent definition of the asymptotic-size spectrum that we adopt is the log of the pdf of η_∞_ for individuals. The asymptotic-size spectrum is linear if and only if the individual asymptotic-size distribution is a power law, and then its slope is 1 plus the exponent (Appendix S2·1).

The species asymptotic-size distribution is the pdf of *m*_∞_ for species in *R* (Reuman *et al.* 2008, 2009b; Reuman, Cohen & Mulder, 2009a; Fig. 1e). A bin-based definition of the diversity spectrum exists, but the statistically preferable definition is the log of the pdf of η_∞_ for species (Fig. 1f). The diversity spectrum is linear if and only if the species asymptotic-size distribution is a power-law distribution, and then its slope is 1 plus the exponent (Appendix S2·1). The individual size distribution and size spectrum quantify the distribution of individuals' *m*, the individual asymptotic-size distribution and the asymptotic-size spectrum quantify the distribution of individuals' *m*_∞_, and the species asymptotic-size distribution and diversity spectrum quantify the distribution of species' *m*_∞_.

### Model conceptual framework and assumptions

Beginning with notation, denote the asymptotic mass range boundaries for the community by *m*_∞,*l*_ and α*m*_∞,*l*_, where *l* stands for ‘lower bound’ and α is a factor >1. This range has width log(α) on a logarithmic scale, and represents a moving window on that scale. Denote by *f*(*m*_∞_) a minimum mass cut-off larger than the eggs of individuals of asymptotic mass *m*_∞_ – this describes egg mass as a function of asymptotic mass. Denote by *C*(*m*_∞,*l*_, α*m*_∞,*l*_) the community of individuals in region *R* with asymptotic body mass, *m*_∞_, in the range *m*_*∞,l*_ to α*m*_*∞,l*_ and body mass, *m*, in the range *f*(*m*_∞_) ≤ *m* ≤ *m*_∞_. Denote by *M*(*m*_*∞,l*_, α*m*_*∞,l*_) the metacommunity, delimited by the same ranges of *m*_∞_ and *m*, but in the region outside *R* instead of in *R*. The region *R* is assumed to be 10000 km^2^ or larger. Denote by *J*_*C*_(*m*_*∞,l*_, α*m*_*∞,l*_) and *J*_*M*_(*m*_*∞,l*_, α*m*_*∞,l*_) the numbers of individuals in the community and metacommunity, respectively, and denote by *S*_*C*_(*m*_*∞,l*_, α*m*_*∞,l*_) and *S*_*M*_(*m*_*∞,l*_, α*m*_*∞,l*_) the numbers of species represented in each. The abbreviations *C, M, J*_*C*_*, J*_*M*_*, S*_*C*_ and *S*_*M*_ are used when *m*_*∞,l*_ and α*m*_*∞,l*_ are understood from context. *T* and *A*_*R*_ denote the average temperature and area, respectively, of *R*.

Model dynamics assume fixed numbers of individuals in the community (*J*_*C*_) and metacommunity (*J*_*M*_), with deaths occurring at random. Dead individuals in the metacommunity are replaced, with probability *ν* by an individual of an entirely new species, and with probability 1−*ν* by the offspring of a randomly chosen individual from the metacommunity. Dead individuals in the community are also replaced, with probability m by the offspring of a randomly chosen individual from the metacommunity, and with probability 1−m by the offspring of a random individual from the community. So *ν* is a speciation rate parameter and m is an immigration rate; these parameters are borrowed from the neutral model (Hubbell 2001). The four model components outlined in the Introduction and derived in the following sections correspond to (1) *J*_*C*_; (2) *J*_*M*_; (3) m; and (4) *ν*. Model parameters are introduced in the text and summarized in Table S1. Following common practice, mathematical symbols in different fonts or with different capitalization are different; notational conventions are explained in full in Appendix S1.

To represent multispecies dynamics in *C* and *M* by the neutral model, we assume the same mortality and reproduction probabilities for all individuals in *C* and *M*, regardless of species (Hubbell 2001). This assumption is not strictly met because niche differences resulting in life-history variation are inevitable, but the assumption is a reasonable first approximation because *m*_∞_ explains much of the variation in life history in marine organisms: the growth trajectory (West, Brown & Enquist, 2001), survival probability and reproductive output of an individual can all be predicted from *m*_∞_ (Charnov 1993; Charnov & Gillooly, 2004). Because growth trajectory is determined by *m*_*∞*_ and because body mass, *m*, which is governed by the growth trajectory, largely explains what a marine organism eats and what eats it (Jennings *et al.* 2001), all organisms in *C* and *M* are considered by our model to face approximately equivalent competitive landscapes, on average, over their lifetimes. There is some variation in egg mass among organisms of asymptotic mass *m*_*∞*_, which could affect the functional equivalence assumption. However, by defining *C* and *M* as those individuals that have grown past the threshold mass *f*(*m*_*∞*_), recruitment to *C* or *M* only occurs at *f*(*m*_∞_). The factor α must be larger than 1, but not so large as to violate the assumption that individuals in *C*(*m*_∞,*l*_, α*m*_*∞,l*_) can be treated as functionally equivalent. The precise value of α does not affect our results.

### Derivation of model components 1 and 2: numbers of individuals *J_C_* and *J_M_*

Theoretical predictions for *J*_*C*_ and *J*_*M*_ are based on a formula of Andersen & Beyer (2006) for the joint distribution, *N*(*m*, *m*_*∞*_), of individual *m* and *m*_*∞*_. Derivation of the formula, with augmentations for the current application, is in Appendix S4. The distribution *N*(*m*, *m*_*∞*_) is defined, as for any distribution, such that *N*(*m*, *m*_∞_)*dmdm*_∞_ is the density in the marine community of individuals with body masses between *m* and *m* + *dm* and asymptotic body masses between *m*_∞_ and *m*_∞_ + *dm*_∞_, for small *dm* and *dm*_∞_.

The formula for *N*(*m*, *m*_∞_) incorporates several well-known parameterizations of aspects of the life history and behaviour of marine organisms. Field metabolic rate, *I*_*F*_, depends on body mass as a power law, 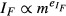 (Appendix S3 and S8·1; see also Clarke & Johnston, 1999; White, Phillips & Seymour, 2006; Hudson, Isaac & Reuman, 2013). Optimal swimming speed, *u*_*opt*_, has been theoretically predicted to be a power law of body mass, 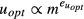 (Ware 1978; Appendix S3), with empirical support provided by Peters (1983). Ontogenetic growth rate, *g*, of marine organisms is known to be well approximated by the formula 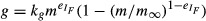 (West, Brown & Enquist, 2001; Andersen & Beyer, 2006; Appendix S4.5). Predation mortality risk in marine systems, *d*, has been parameterized in past work as 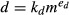 (Lorenzen 1996; Gislason *et al.* 2010). Large teleost fish have small eggs that do not covary in size with species *m*_*∞*_ (Duarte & Alcaraz, 1989; Kamler 2005); and small fish and organisms smaller than fish have egg sizes that scale as a power law of body size (Duarte & Alcaraz, 1989; Hendriks & Mulder, 2008). So *f*(*m*_*∞*_), the upper bound of egg mass for organisms of asymptotic mass *m*_*∞*_, was modelled as 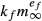 for *m*_*∞*_ less than a threshold *m*_*cut*_, and equal to a constant, *m*_*egg*_, for *m*_*∞*_ ≥ *m*_*cut*_.

The formula for *N*(*m*, *m*_*∞*_) is


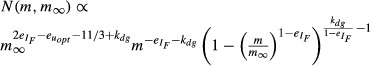
eqn 1

where *k*_*dg*_ = *k*_*d*_*/k*_*g*_. The formula holds for any *m*_∞_ > *m*_*egg*_ and *m* in the range *f*(*m*_∞_) to *m*_∞_. The marginal distribution 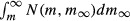 is proportional to the individual size distribution for *m *> *m*_*egg*_. The other marginal distribution, 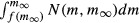, is proportional to the individual asymptotic-size distribution, henceforth denoted 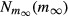, for 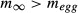.

Via the above proportionality for the individual asymptotic-size distribution, theory predicts how *J*_*C*_ and *J*_*M*_ scale with *m*_*∞,l*_. The numbers of individuals *J*_*C*_ and *J*_*M*_ in the asymptotic mass bounds *m*_*∞,l*_ to α*m*_*∞,l*_ are proportional to 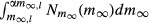. Because 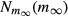 can be computed numerically for *m*_*∞*_ > *m*_*egg*_, this integral can also be computed numerically for *m*_*∞,l*_ > *m*_*egg*_, providing the predictions for the scaling of *J*_*C*_ and *J*_*M*_.

The derivation of the scaling of *J*_*C*_ and *J*_*M*_ also reveals that this scaling is independent of the temperature and area of *R* and its metaregion: although the constants of proportionality relating *J*_*C*_ and *J*_*M*_ to 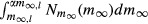 will differ from each other and may vary from one region, *R*, to another, the proportionalities themselves are the same for all regions. Eqn 1 and hence the proportionalities for the individual asymptotic-size distribution, *J*_*C*_, and *J*_*M*_, were derived regardless of temperature and region area (Andersen & Beyer, 2006; Appendix S4). The parameters in eqn 1 also do not depend on these environmental factors (Appendix S4·6).

The quotient *J*_*C*_/*J*_*M*_ turns out to be important for the diversity spectrum and its variation among regions. It will later be shown to be proportional to the ‘size of *C* relative to speciation’, a quantity defined as the number of individuals in the community *C* compared to a total number of speciation events in the metacommunity per generation. Therefore, we now explore the dependence of *J*_*C*_/*J*_*M*_ on *m*_*∞,l*_, *T* and *A*_*R*_. The *m*_*∞,l*_ dependence of *J*_*C*_/*J*_*M*_ follows immediately: because *J*_*C*_ and *J*_*M*_ scale with *m*_*∞,l*_ in the same way, *J*_*C*_/*J*_*M*_ is independent of *m*_*∞,l*_.

The parameter *J*_*C*_/*J*_*M*_ is smaller for regions with higher *T*. In marine ecosystems, temperature and primary production are not strongly positively related on the spatial scales we consider because low nutrient availability limits primary production in areas with intense solar radiation and because currents as well as local solar heating drive sea temperature (Sarmiento & Gruber, 2006). Thus, in warm regions, resource supply rate at the base of the food web is not systematically larger than in cold regions. Because the metabolic demands of heterotrophic marine ectotherms are greater in warmer regions, *J*_*C*_ will be smaller, on average, in warmer regions. A similar result is obtained by Jennings *et al*. (2008). But the metaregions will not vary appreciably in their average temperatures from one region, *R*, to the next because the metaregions themselves will not vary much: the metaregion is the area outside the region, and the area outside one reasonably sized region nearly coincides with the area outside another. Therefore, the metacommunity sizes, *J*_*M*_, will be nearly constant for reasonably sized regions. Because *J*_*C*_ is smaller and *J*_*M*_ nearly the same for warmer regions, *J*_*C*_/*J*_*M*_ will be smaller for warmer regions, as claimed.

The quotient *J*_*C*_/*J*_*M*_ is also larger for regions with larger area *A*_*R*_. In fact, for each increase in *A*_*R*_ by an arbitrary factor, γ, the ratio *J*_*C*_/*J*_*M*_ will be larger by a factor of at least γ: *J*_*C*_ is proportional to *A*_*R*_ and *J*_*M*_ shrinks as *A*_*R*_ increases because the metaregion is the area outside the region.

### Derivation of model component 3: dispersal, m

In the neutral model, deaths occur at random and each dead individual in the community is replaced from the metacommunity with probability m and from the community with probability 1−m. For a tractable derivation of m, we idealize the layout of *R* as a disc, *D*, of effective radius 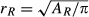 in the real Euclidean plane. The disc *D* contains the community, *C*, and the region in the Euclidean plane outside *D* contains the metacommunity, *M*. In the event of a death at location **v**_1_ = (*x*_1_, *y*_1_) in *D*, let 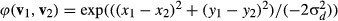 be the relative pressure from any other point **v**_2_ = (*x*_2_, *y*_2_) to fill the vacancy resulting from this death; *φ* is a dispersal kernel, and σ_*d*_ is a dispersal distance parameter for organisms in *C* and *M*. The average total replacement pressure from outside *D* divided by that from inside *D* is
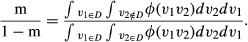
eqn 2

This quotient simplifies to provide an expression for m (eqn S5·1·8, Appendix S5·1) that can be computed numerically given *r*_*R*_/σ_*d*_, therefore providing theoretical predictions for the dependence of m on *m*_*∞,l*_, *T* and *A*_*R*_ if we can now predict how *r*_*R*_/σ_*d*_ depends on these variables.

But a relationship of the form 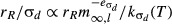 can be derived, where 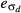 is positive and 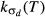 is smaller for larger *T*. We here derive it by deriving the formula 
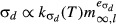
, from which the formula for *r*_*R*_*/σ*_*d*_ follows immediately. The formula for σ_*d*_ is empirically and theoretically supported for both larval and adult dispersal. A large portion of dispersal in a marine environment is via planktonic or weakly swimming larvae. Species dispersal distances, σ_*d*_, as inferred from genetic data and the expansion rates of invasive species, or as measured directly for dispersing larvae, have been found to be strongly related to larval duration by a power law (Shanks, Grantham & Carr, 2003; Siegel *et al.* 2003). Larval duration is in turn related to maximum body mass via a power law (Bradbury *et al.* 2008) and has also been shown to decrease with increasing *T* both within and among species (O'Connor *et al.* 2007; Bradbury *et al.* 2008). Combining these patterns supports the stated dependence of σ_*d*_ on *m*_*∞,l*_ and *T* if dispersal is primarily larval. Adult dispersal is reasonably assumed to be proportional to *u*_*opt*_ times life span. Theory in Appendix S3 shows 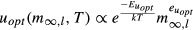. Life span is known to scale approximately as inverse mass-specific metabolic rate, which scales as 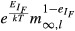 because metabolic rate is proportional to 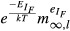 (Gillooly *et al.* 2001). Multiplying the expressions for *u*_*opt*_ and life span gives 
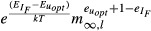
. This product is a positive-exponent power law of *m*_*∞,l*_, as claimed, as long as 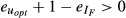, and it decreases as *T* increases, as claimed, as long as 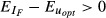, supporting the stated dependence of σ_*d*_ on *m*_*∞,l*_ and *T* if dispersal is primarily by adults. See Appendix S5·2 for a more detailed theoretical development that comes to the same conclusions. A positive relationship between dispersal and asymptotic body mass was further empirically supported by a negative correlation between the genetic differentiation within species and species' maximum body size (Bradbury *et al.* 2008). Decreased dispersal at higher *T* was supported in the same study by a negative correlation between species' genetic differentiation and their maximum latitude.

Given the relationship 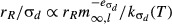, we let the unknown value of *r*_*R*_/σ_*d*_ for the reference asymptotic mass *m*_*egg*_ be denoted *K*_1_, so that 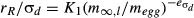 and 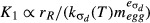. Given values for *K*_1_, *m*_*egg*_ and 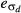, *r*_*R*_/σ_*d*_ and therefore m can be computed for any *m*_*∞,l*_. Theory thereby provides predictions for how m depends on *m*_*∞,l*_. We call *K*_1_ the *relative radius of the region R*. It is the effective radius of the region relative to the dispersal kernel of the smallest mass category, and turns out to be important for the diversity spectrum of *R*. The dependence of m on the environmental variables *T* and *A*_*R*_ is through *K*_1_ because *r*_*R*_/σ_*d*_ depends on *T* and *A*_*R*_ through *K*_1_. *K*_1_ is larger for warmer or larger regions. The expression 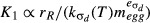 makes it clear that higher *T* implies larger *K*_1_, because 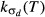 is smaller for higher *T*; and increasing *A*_*R*_ by an arbitrary factor γ increases *r*_*R*_ and therefore *K*_1_ by a factor of 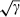.

### Model component 4: speciation, *ν*

Prior work suggestively but not irrefutably supports the assumption that *ν* is independent of *m*_*∞,l*_ and *T*. Gillooly *et al*. (2005) showed that molecular evolution rates, in units of nucleotide substitutions per unit time and per site in a genome, are proportional to species characteristic body mass to the power of −1/4, times 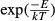, where *E* is about the same as the Arrhenius activation energy of metabolism. As generation time is approximately proportional to the inverse of this product, molecular evolution rates expressed in units of nucleotide substitutions per generation and per site are independent of body mass and temperature. Thus, rates of molecular evolution per recruit are mass and temperature independent. Because the parameter *ν* is a per-recruit rate, this reasoning would support the constancy of *ν* if speciation rates are primarily controlled by rates of genetic divergence, as suggested by Allen *et al*. (2006). Thomas *et al*. (2006) found that rates of molecular evolution in invertebrate taxa do not depend systematically on body mass, casting doubt on the generality of the results of Gillooly *et al*. (2005), but Thomas *et al*. (2006) did not control for temperature. Perhaps more importantly, the link between molecular evolution and morphological change and speciation is uncertain (Bromham *et al.* 2002). Several of these points were made by Mittelbach *et al*. (2007). Nevertheless, because the assumption of constant *ν* is better supported than alternative assumptions and is also more parsimonious, we explore the consequences of this assumption for our model instead of alternative assumptions.

### The unifying model component: numbers of species, *S_C_* and *S_M_*

Formulas were provided by Etienne & Olff (2004) for the numbers of species *S*_*M*_ and *S*_*C*_, depending on the quantities *J*_*C*_, *J*_*M*_, m and *ν* derived above. The formulas provide expected numbers of species at stochastic equilibrium in the neutral model. The formulas can be well approximated by
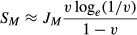
eqn 3
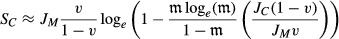
eqn 4(Appendix S6·2). These approximations are used below to develop testable predictions. The inaccuracies of the formulas and their effects on our conclusions are quantified and shown to be negligible in Appendix S9. We use approximations in place of the original formulas of Etienne & Olff (2004) because it simplifies analysis and interpretations.

Because *ν* is very small, the expression *J*_*C*_ (1 − *ν*)/(*J*_*M*_*ν*) in eqn 4 is approximately *J*_*C*_/(*J*_*M*_*ν*), the number of individuals in *C* relative to the number of speciation events in *M* per generation. We denote this constant by *K*_2_, which we name the *size of C relative to speciation*. *K*_2_ is constant with respect to *m*_*∞,l*_ because *ν* is constant and we showed that *J*_*C*_ and *J*_*M*_ scale in the same way with respect to *m*_*∞,l*_. *K*_2_ is smaller for regions with higher *T*, and, for each increase in *A*_*R*_ by an arbitrary factor, γ, *K*_2_ is larger by a factor of at least γ; these facts hold because *ν* is constant and we showed that *J*_*C*_/*J*_*M*_ has the same properties.

## Model parameterization

### Model parameters

The model was parameterized from data in the literature. The values 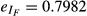 and 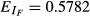 were derived from our own theoretical and empirical analyses (Appendix sections S3 and S8.1). Similar values were estimated from a large data set in prior work (Clarke & Johnston, 1999). The value 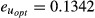 was derived theoretically (Ware 1978; Appendix sections S3 and S8.5) and supported empirically by Peters (1983), who obtained the value 0·13. 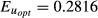 was obtained from the same theory. Because *k*_*dg*_ is a function of 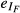, 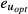, β_*f*_, σ_*f*_ and a food conversion efficiency, the value *k*_*dg*_ = 0·737 was derived from literature estimates of these parameters (Appendix S8.5). To parameterize *f*(*m*_*∞*_), data from several sources on the sizes of the eggs of fish and other marine organisms were used to support the values *m*_*egg*_ = 6·5 × 10^−5^ kg, *m*_*cut*_ = 0·316 kg, *e*_*f*_ = 0·5 and 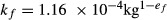 (Appendix S8.4).

We adopted a range of values for the dispersal parameter 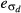 because it was the parameter known with the least certainty from the literature and because it can comprise both larval and adult dispersal. Dispersal distance is related to larval duration by a power law with exponent 1 (Siegel *et al.* 2003; Shanks, Grantham & Carr, 2003), and larval duration is related to maximal body mass by a power law with exponent 0·25 (Bradbury *et al.* 2008). Thus 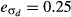 if dispersal is primarily larval. For adults, we derived 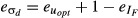 in the Model predictions, the value of which is 0·336. The same or very similar values were obtained by the more detailed reasoning in Appendix S5·2 (see also Appendix S8·6). We considered the range 0·2 to 0·4 for 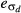. The value 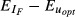, which if positive indicates that adult dispersal is theoretically expected to be reduced at higher temperatures (see Derivation of model component 3: dispersal, *m*), is 0·5782–0·2816 = 0·2966 > 0. Additional details on model parameters are given in Appendix S8, and parameter values are summarized in Table S1.

### Bounds for *k*_1_ and *K*_2_

Two bounds can be derived within which *K*_1_, the relative radius of the region, and *K*_2_, the size of *C* relative to speciation, must reasonably lie for any of the regions we consider. These bounds are important for understanding the range of possibilities for diversity spectra because *K*_1_ and *K*_2_ affect the diversity spectrum of *R*. By definition, *K*_2_ = *J*_*C*_(1−*ν*)/(*νJ*_*M*_), where *νJ*_*M*_ is a measure of the commonness of speciation and *J*_*C*_ (1−*ν*) ≈ *J*_*C*_ is large for large regions *R*. Because speciation is rare and we consider only regions of area more than 10 000 km^2^, it is safe to assume *K*_2_ > 10, that is, that the number of new species per generation is not more than 1/10th the population of the region *R*. This seems likely to be a conservative bound. The bound applies for the smallest regions we consider (those of area 10 000 km^2^); the higher bound *K*_2_ > (*A*_*R*_/10 000 km^2^)10 therefore holds for larger regions. An upper bound for *K*_1_ that depends on *A*_*R*_ can also be produced: solving 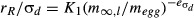 for *K*_1_ and substituting 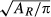 for *r*_*R*_ gives 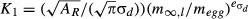. Using the reasonable assumption that σ_*d*_ > 10 km for organisms of asymptotic mass 1000 kg (and σ_*d*_ is certain to be larger than 10 km for such large organisms, which on energetic grounds alone would need to forage over extensive areas), we have 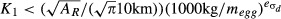. This bound for *K*_1_ can be combined with the bound for *K*_2_ by algebraically eliminating *A*_*R*_ to get 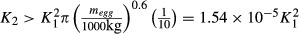, using the central value 0·3 for 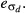 Both bounds are linear on log(*K*_2_)-versus-log(*K*_1_) axes. Details of the derivation are in Appendix S8·7.

## Model predictions

### Abundance predictions

**Fig 2:**
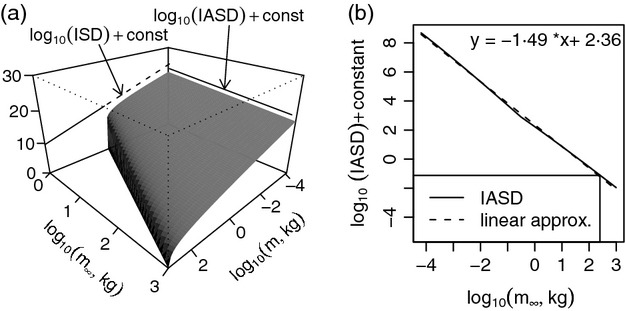
(a) The joint distribution of individual mass, *m*, and asymptotic mass, *m*_∞_, expressed as log_10_(*N*(*m*, *m*_∞_)) +  constant (see eqn 1) for *m* between *m*_*egg*_ (upper bound fish egg size) and 1000 kg and *m*_∞_ between 1 and 1000 kg. The marginal distributions, which are the individual size distribution (ISD) and the individual asymptotic-size distribution (IASD), are labelled. The dashed line in the individual size distribution indicates the part of the plot to which organisms with *m*_∞_ < 1 kg contribute. (b) The log_10_ individual asymptotic-size distribution plotted and linearly approximated for *m*_∞_ between *m*_*egg*_ and 1000 kg, illustrating the theoretical prediction that the individual asymptotic-size distribution is approximately a power law in *m*_∞_ with exponent about −1·49.

Linearity of the size spectrum and slope about −1 have been empirically supported (e.g. Sheldon, Sutcliff & Prakash, 1972; Kerr & Dickie, 2001). Our model predicts this, providing reassurance of model reasonableness. The distribution *N*(*m*, *m*_*∞*_), using the parameters of section Model parameters, is pictured in Fig. 2a. The marginal distribution 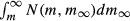, proportional to the individual size distribution, was shown by Andersen & Beyer (2006) to be a power-law distribution with exponent −2·003. The model of Andersen & Beyer (2006) is included in our model; hence, our model also predicts a power-law individual size distribution with exponent −2·003 and therefore a linear size spectrum of slope −1·003 (Fig. 2a). The derivation is reproduced in Appendix S4·3.

The other marginal distribution of *N*(*m*, *m*_*∞*_), computed numerically and proportional to the individual asymptotic-size distribution, is shown in Fig. 2b. The predicted log_10_ individual asymptotic-size distribution is approximately linear in log_10_(*m*_∞_), of slope −1·49. Hence, theory predicts that the individual asymptotic-size distribution is a power law in *m*_∞_ with exponent −1·49, and the asymptotic-size spectrum is linear with slope −0·49.

### Diversity spectra

Because the individual asymptotic-size distribution is approximately a power law in *m*_∞_ with exponent −1·49, *J*_*C*_ and *J*_*M*_ are approximately proportional to 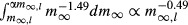. As *ν* is constant with respect to *m*_*∞,l*_, eqn 3 implies that *S*_*M*_ should scale with *m*_*∞,l*_ in the same way *J*_*M*_ does, leading to **Prediction 1**: The number of species *S*_*M*_ in the metacommunity *M* is approximately a power law in *m*_*∞,l*_ with exponent −0·49. Equivalently, the diversity spectrum of the metaregion is approximately linear with slope −0·49. This prediction is for *m*_*∞,l*_ > *m*_*egg*_, a limitation which comes from the same limitation for eqn 1. Thus, theory predicts that the number of species in a category of log asymptotic mass in the metaregion will be proportional to the number of individuals in that category.

**Fig 3:**
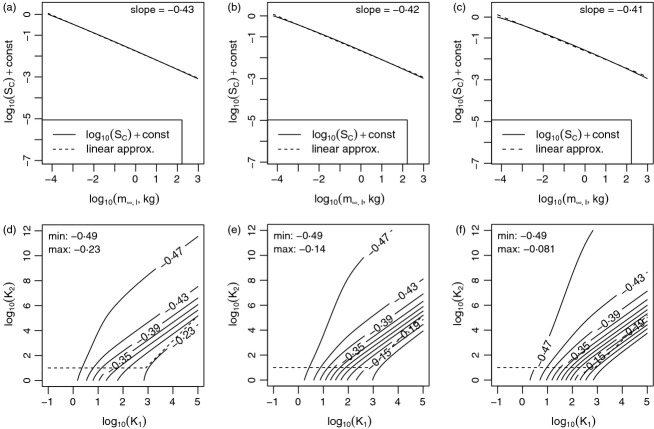
Predicted regional diversity spectra and their slopes for different values of *K*_1_ (the relative radius of the region), *K*_2_ (the size of the community relative to speciation) and 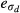 (the dispersal distance scaling exponent). Examples of predicted regional diversity spectra (a–c) were close to linear. These panels show the log_10_ number of species in the region *R* (i.e. *S*_*C*_(*m*_∞,*l*_, *αm*_∞,*l*_)) plotted for lower-bound asymptotic mass *m*_∞,*l*_ between *m*_*egg*_ and 1000 kg. *S*_*C*_ is computed using eqn 4. *K*_1_ = 10^2·5^ and *K*_2_ = 10^4^ were used for a–c; 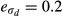, 0·3 and 0·4 were used for a, d; b, e; and c, f, respectively, spanning the range selected in the section Model parameters. The relationship between log_10_(*S*_*C*_) and log_10_(*m*_∞,*l*_) was always close to linear, not just in the examples shown (see text). Panels d–f are contour plots showing slopes of log_10_(*S*_*C*_) versus log_10_(*m*_∞,*l*_) for a range of values of 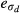, *K*_1_, and *K*_2_. Dashed lines in d–f delineate the bounds for *K*_1_ and *K*_2_ given in the section Bounds for *k*_1_ and *K*_2_. The minimum slope and maximum slope occurring in the bounds are given, and indicate that regional diversity spectrum slopes should be between −0·5 and about −0·1 for real regions.

Predictions for the dependence of *S*_*C*_ on *m*_*∞,l*_ can be computed numerically using eqn 4 for any given values of 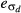. *K*_1_ and *K*_2_. We plotted log_10_(*S*_*C*_) against log_10_(*m*_*∞,l*_) (the diversity spectrum) for values of 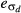 in the range 0·2 to 0·4 and for *K*_1_ and *K*_2_ in a region bounded by the constraints of the section Bounds for *k*_1_ and *K*_2_. Plots were always close to linear: root mean squared deviations between linear approximations to the plots and the plots themselves were always < 0·175 (Fig. 3a–c for example plots). Slopes were always shallower than (greater than) −0·49 and were steeper than (less than) about −0·1 for reasonable values of *K*_1_ and *K*_2_ (Fig. 3d–f). These results precipitate two predictions. **Prediction 2**: The number of species *S*_*C*_ in the community *C* is approximately a power law in *m*_*∞,l*_. Equivalently, the diversity spectrum of the region *R* is linear. **Prediction 3**: The power-law exponent is greater than −0·49 and likely less than −0·1 for large regions. Equivalently, the diversity spectrum slope for *R* is between −0·49 and −0·1. These predictions are for *m*_*∞,l*_ > *m*_*egg*_. A derivation that does not use the approximations used here is in Appendix sections S9·2 and S9·3; results are substantially the same.

### Environmental gradients in diversity spectra

**Fig 4:**
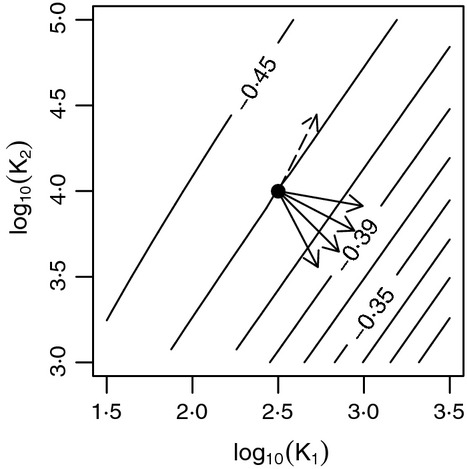
Predicted variation in regional diversity spectrum slopes along environmental gradients. Contour lines show diversity spectrum slopes, enlarging part of Fig. 3e. Starting from reference values of *K*_1_ and *K*_2_ (solid dot), arrows show the predicted variation in *K*_1_, *K*_2_ and diversity spectrum slope along gradients of increasing temperature, *T* (solid arrows, several possible outcomes shown) and increasing region area, *A*_*r*_ (dashed arrow). Diversity spectrum slopes are predicted to become shallower with increasing *T* and steeper with increasing *A*_*r*_. Arrows show directions of predicted effects but not magnitudes. Results are similar for other values of 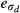 (Fig. 3).

Suppose given a collection of continental shelf-sea regions with different average temperatures, *T*, and areas, *A*_*R*_; the collection of regions has a collection of associated metaregions, each metaregion being the area outside its region. We showed that *K*_1_ is predicted to be larger and *K*_2_ smaller for warmer regions than for colder ones. Therefore, Fig. 3d–f leads to **Prediction 4**: Diversity spectrum slopes will be shallower (less negative) in warmer regions. Moving to the right (increasing *K*_1_) and down (decreasing *K*_2_) on any of the panels d–f of Fig. 3 implies a shallower predicted slope (see Fig. 4 for a detailed depiction for the central value 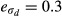). This prediction is for *m*_*∞,l*_ > *m*_*egg*_. It holds as long as *T* and net primary productivity are truly not positively related among regions; the derivation of the *T* dependence of *J*_*C*_/*J*_*M*_, and therefore *K*_1_, relied on this expectation.

We showed that across a gradient of increasing *A*_*R*_, both *K*_1_ and *K*_2_ are predicted to increase, with *K*_1_ being larger by a factor of 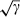 and *K*_2_ being larger by a factor of at least γ for each factor-of-γ increase in *A*_*R*_. The net effect is **Prediction 5**: The diversity spectrum of larger regions will be steeper (more negative) than that of smaller regions (Fig. 4). This prediction is for *m*_*∞,l*_ > *m*_*egg*_. The prediction matches with intuition because larger regions are closer in size to their associated metaregions, which have predicted diversity spectrum slope −0·49, at the steep end of the range of predicted regional slopes. A derivation of predictions 4 and 5 that does not use the approximations used here is in Appendix S9·3; results are substantially the same.

## Methods for testing model predictions

To test theoretical predictions, we empirically estimated diversity spectra of 63 of the 64 large marine ecosystems (LMEs) that partition the world's continental shelf seas (Sherman, Alexander & Gold, 1993). LME boundaries are standardized and are delineated by downloadable GIS shapefiles from the United States National Oceanic and Atmospheric Administration (NOAA; Table S3 and Fig. S7). LMEs are large, the smallest having area 1·52 × 10^11^ m^2^. The Arctic LME was excluded because environmental variables were unavailable.

The range of variation in LME areas was modest (1·52 × 10^11^ to 4·17 × 10^12^ m^2^, a factor of 27·3). Therefore, to examine the influence of region area on diversity spectra, LMEs were also aggregated to form larger regions for which diversity spectra were estimated. LMEs were aggregated to form 15 ‘provinces’ of area 9·77 × 10^11^ to 1·85 × 10^13^ m^2^, 7 ‘basins’ of area 2·53 × 10^12^ to 2·55 × 10^13 ^m^2^, 3 ‘latitudinal bands’ of area 1·69 × 10^13^ to 3·13 × 10^13 ^m^2^ and a single aggregate of all 63 LMEs, the ‘global region’ (area 7·58 × 10^13 ^m^2^). Regions are listed and mapped in Appendix S10·1, Table S4 and Figs S8 to S10.

Our theory applies to the entire community for *m*_*∞,l*_ > *m*_*egg*_ and is not constrained to a taxonomic group. However, taxonomically inclusive data are very difficult to obtain. To test our theory, we use the fact that fish dominate the biomass and diversity of marine pelagic ecosystems over the asymptotic mass range 1 to 1000 kg and are likely to provide an adequate representation of the whole community in that range (Jennings *et al.* 2008). Theory was tested using fish data in that range. The effect of including other groups such as mammals, cephalopods and scyphozoans was considered to the extent possible.

Data on the asymptotic sizes of fish species and their occurrence by LME were downloaded from FishBase (Froese & Pauly, 2006, August 2013). FishBase provides the maximum length ever observed in any ecosystem for each species. This was taken as a surrogate for species asymptotic length, *l*_∞_. Asymptotic lengths were converted to asymptotic masses via the relationship *m*_∞_=10^1.038^ *l*_∞_^2.541^, where *l*_∞_ is in metres and *m*_∞_ is in kilograms. This relationship was determined from mass and length data for world-record fish caught by angling, for 526 species, from the International Game Fish Association; world-record lengths and masses are taken as surrogates for asymptotic lengths and masses for a species, and an interspecific regression was carried out to approximate the relationship between asymptotic mass and length (Appendix S11·1 for details). The same value of *m*_∞_ was used for a species in all LMEs in which it occurred. We excluded species in the FishBase life-type category ‘reef-associated’ because our theory is for pelagic species. The number of LME species occurrence records extracted from FishBase was 27 817. Lists of species for larger regions (provinces, basins, etc.) were compiled by combining the species lists for component LMEs and removing duplicates.

The diversity spectrum of a region was estimated by estimating the mathematically equivalent species asymptotic-size distribution. A truncated Pareto (tP) distribution was fitted by maximum likelihood (Aban, Meerschaert & Panorska, 2006) to the species asymptotic mass data between 1 and 1000 kg for each region that had at least 30 species in that range (58 LMEs and all the larger regions).

The quality of fit of the tP distribution, and hence the correctness of theoretical predictions about the linearity of diversity spectra, was assessed with statistical tests and plots. For each region, we tested the composite hypothesis that data came from a tP distribution with truncation points 1 and 1000 kg and unknown exponent. The statistical test used is based on the Kolmogorov–Smirnov statistic (Appendix S10·2). Tests such as this one can detect very small deviations from the null hypothesis for large sample sizes. For the speciose LMEs and for the larger regions, sample sizes were large, so we also produced plots that depict the magnitude of deviations from linearity. These plots were produced using a simple transformation that converts the cumulative distribution function (cdf) of a tP distribution to the associated diversity spectrum (Appendix S10·2). The transformation was applied to the empirical cdf of each region, producing a plot we call the *empirical diversity spectrum*. The plot was compared to the diversity spectrum associated with the fitted tP distribution. Agreement between the plots was assessed visually and also using a coefficient of determination, 1 − SSE/SST, where SSE was the sum of squared differences between the two plots, and SST was the sum of squared deviations of the empirical diversity spectrum from its mean. This statistic, which we call the *spectrum linearity statistic*, is the fraction of the variation in the empirical diversity spectrum that is explained by the linear hypothesis. If the spectrum linearity statistic was greater than 98% for a region, the diversity spectrum of that region was deemed linear for the purposes of this study even if the tP distribution was statistically rejected by the above test. This is reasonable because we are trying to understand the most important determinants of diversity patterns.

For regions for which the tP distribution was statistically rejected, a quadratic generalization of the tP distribution, here called the *quadratic truncated Pareto* (qtP) distribution (Reuman *et al*. 2008; Appendix S2·2), was also fitted for confirmation. Its fit was compared with that of the tP distribution using a likelihood ratio test. The qtP is mathematically equivalent to a quadratic diversity spectrum; hence, our comparison of the tP and qtP distributions constituted a comparison of the hypothesis of a linear diversity spectrum against a quadratic alternative. The qtP distribution is the same as a log-normal distribution truncated on both sides, and the log-normal distribution is a commonly considered hypothesis for species size distributions. The quality of fit of both the tP and qtP distributions was judged visually by plotting log_10_ asymptotic body masses, sorted in ascending order, against log_10_-scale medians of the order statistics of the fitted distributions, to provide log_10_-scale probability plots. When these plots were straight it indicated that the distribution used was a good fit. When the plot for the qtP distribution was not substantially straighter than that for the tP distribution, it indicated that the null hypothesis of a linear diversity spectrum was at least as good as the curved alternative. For regions for which the tP distribution was statistically rejected, the diversity spectra corresponding to both the fitted tP and qtP distributions were also plotted and compared visually. The dual use of formal hypothesis tests and visual comparisons is again appropriate because we are interested in whether theory and data agree on major patterns, but minor deviations can cause statistical rejection of hypotheses for large data sets.

Slopes of diversity spectra that were deemed linear (either the tP distribution was not statistically rejected or the spectrum linearity statistic was > 98%) were retrieved from the parameters of the best-fitting tP distribution. The tP distribution has pdf proportional to 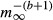 where *b* is the fitted parameter. The diversity spectrum is linear, and −*b* is the diversity spectrum slope if the tP distribution is a good fit (see section Preliminaries: spectra and distributions; Appendix S2·1). Confidence intervals for *b* and therefore for diversity spectrum slope were obtained by a resampling scheme. For each region, *m*_∞_ values in the range 1 to 1000 kg were resampled 1000 times with replacement, and *b* was estimated for each resampling, with quantiles providing confidence intervals.

Estimates of average sea surface temperature, used as a surrogate for *T*, and primary production in the LMEs were obtained from remote sensing data. *T* estimates were averages of 1997–2007 outputs of the version 5·0 Advanced Very High Resolution Radiometer Pathfinder project conducted by the University of Miami's Rosenstiel School of Marine and Atmospheric Science and the NOAA National Oceanographic Data Center. The Pathfinder data set is distributed by the Physical Oceanography Data Active Archive Center (PODAAC) of the United States National Aeronautics and Space Administration Jet Propulsion Laboratory. Net primary productivity was depth-integrated primary production (mg C m^−2^ d^−1^) and was calculated from chlorophyll concentration following the approach of Platt & Sathyendranath (1988) as implemented by Mélin & Hoepffner (2004). Model inputs of surface chlorophyll concentration were obtained from the Sea-viewing Wide Field-of-view Sensor (SeaWiFS) time series for the years 1997–2007. Averaging procedures are in Appendix S10·3. The Arctic LME was not included because near-continuous cloud and ice cover prevented adequate estimates of environmental variables (Gregg & Casey, 2007).

Dependence of diversity spectrum slopes on environmental variables was examined with linear models, using those LMEs deemed to have adequately linear diversity spectra. A linear model with predictors log_10_(*A*_*R*_) and *T* was used. *A*_*R*_ was log-transformed because the transformed variable appeared symmetrically and unimodally distributed. For verification of results, a linear model was also used in which the importance of individual systems for fitting was weighted according to the inverse variances of the diversity spectrum slope estimates.

## Results of testing model predictions

**Fig 5:**
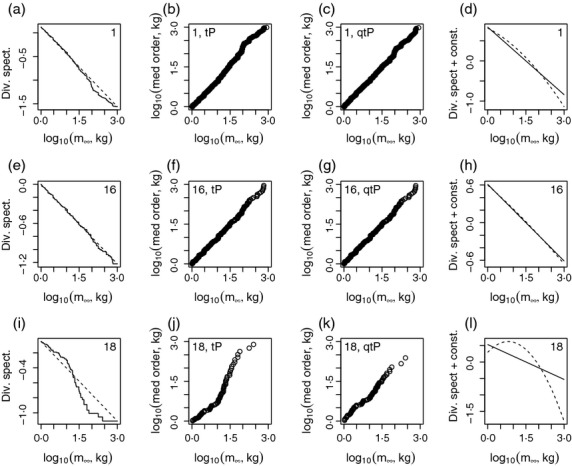
Example results for testing the hypothesis that diversity spectra are linear. Empirical diversity spectra (see the section Methods for testing model predictions) and diversity spectra corresponding to fitted tP distributions for selected regions (a, e, i). Log-scale probability plots for truncated Pareto (tP; b, f, j) and quadratic truncated Pareto (qtP; c, g, k) fits. Comparison of diversity spectra corresponding to tP and qtP fits (d, h, l). Panels are as follows: a–d, the global region (all 63 LMEs combined); e–h, the Brazil Shelf; i–l the West Greenland Shelf. Numeric codes in the upper corners also identify regions – Tables S3 and S4 list the system names that correspond to the codes. See Fig. S11 for other regions.

Prediction 1 was validated in main substance: the metaregion diversity spectrum was approximately linear with slope close to −0·49. The metaregion corresponding to any of our regions, *R*, is the area of the global region outside *R*, which is well approximated by the whole global region. So we tested prediction 1 for the global region. Although the tP distribution was statistically rejected at the 1% level (Table S6 for *P*-values), the empirical diversity spectrum was very close to linear (Fig. 5a), and the spectrum linearity statistic was greater than 98% (Table S6 for spectrum linearity statistics). Because sample size was large for the global region (*n *=* *2885), very small deviations from linearity were detected by the test of fit of the tP distribution. Probability plots indicated that the tP distribution was a good, but not perfect fit for the global region (Fig. 5b). A likelihood ratio test showed that the qtP distribution was statistically preferred (1% level) to the tP distribution, but the qtP probability plot was only slightly straighter than the tP plot (Fig. 5c), and the diversity spectrum corresponding to the best-fitting qtP was hardly different from that of the best-fitting tP (Fig. 5d). Thus, the spectrum deviated significantly but only slightly from linearity. These deviations are real features of the data, but they do not influence our understanding of broad patterns in diversity, because they are small compared with the overall pattern.

The diversity spectrum slope estimated by fitting the tP distribution was −0·561, with 95% confidence intervals (−0·585, −0·536) and 99% intervals (−0·590, −0·532). These intervals did not contain the predicted slope, −0·49, but were close to it, possibly indicating that the model contains the most important mechanisms controlling the diversity spectrum but omits some less influential mechanisms. Alternatively, model-data deviations may be because we used fish to approximate the whole community. Although fish are expected to dominate marine pelagic biomass and diversity in the *m*_*∞*_ range 1 to 1000 kg (Jennings *et al.* 2008), to precisely evaluate the accuracy of this approximation would require the compilation of a large amount of data for other groups, probably not currently possible for the global region. We instead examined the approximation by looking at the group other than fish that seems most likely to contribute diversity that may affect estimates of diversity spectrum slopes: marine mammals. Marine mammals are large and hence contribute diversity to the upper end of the range 1 to 1000 kg. Estimates of slope are most sensitive to additional diversity at the upper end of the range, where there are few species. Of approximately 120 known, extant marine mammals, body mass data were provided by Smith *et al*. (2003) for 113, of which 80 had average body mass in the range 1 to 1000 kg. When these 80 mammals were combined with the 2885 fish species in the global region, the tP distribution was still a good description, with spectrum linearity statistic greater than 98% (Fig. S12; Table S5), and the slope was even closer to model predictions (slope −0·521, 95% confidence intervals (−0·544, −0·498) and 99% intervals (−0·551, −0·494)).

Prediction 2 was generally validated, but with a few interesting exceptions: regional diversity spectra were usually, but not always linear or very close to linear. Of the 58 LMEs for which sufficient data were available, the tP distribution was statistically rejected (1% level) and spectrum linearity statistics were less than 98% for only five LMEs, namely the Baltic Sea, the Faroe Plateau, the Iceland Shelf, the Norwegian Sea and the West Greenland Shelf. Empirical diversity spectra were close to linear except for these examples (Figs 5 and S11). Probability plots confirmed that the tP distribution was a reasonable fit, and comparison between tP and qtP fits revealed only small differences, except for the five exceptional examples (Figs 5 and S11). These five systems had empirical diversity spectra that were clearly not straight, spectrum linearity statistics less than 98% and probability plots that indicated substantial nonlinearity (Figs 5i–l and S11 and Table S6). The qtP was preferred to the tP for these systems, that is, spectra were curved. These systems violated theoretical predictions for unknown reasons. These LMEs were all located in the same area. Three of them (the West Greenland Shelf, the Iceland Shelf and the Norwegian Sea) were part of the North Atlantic province, which was the only province for which the tP distribution was rejected and the spectrum linearity statistic was less than 98%. Basins and latitudinal bands were deemed linear (either the tP was not rejected or the spectrum linearity statistic was greater than 98%), except for the South Atlantic basin, which was close to linear, with spectrum linearity statistic 0·978. Diversity spectra were thus generally close to linear, validating prediction 2, except for some atypical LMEs in the North Atlantic.

Prediction 3 was validated: estimates of regional diversity spectrum slopes were broadly consistent with the predicted range −0·5 to −0·1. Of the 53 LMEs with adequately linear diversity spectra, none had estimated slope above −0·1, only six had slopes below −0·5, only three had 95% confidence intervals of the slope that did not overlap with the range −0·5 to −0·1, and only two had 99% intervals that did not overlap with the range (the Antarctic and Sea of Okhotsk). Other than the Antarctic, all provinces, basins and latitudinal bands had confidence intervals that overlapped with the range −0·5 to −0·1 (Table S5).

Before testing predictions 4 and 5, we tested the underlying assumption about the regions, *R*, that is, that temperature, *T*, for the regions was not positively related to net primary productivity. Across the 53 LMEs for which sufficient data were available to estimate diversity spectra and for which diversity spectra were linear, *T* and net primary productivity were actually significantly negatively related (*R* = −0·326, *P* = 0·017). The association was weak. Similar results held using log_10_ net primary productivity (*R* = −0·347, *P* = 0·011). *T* and 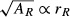 were not significantly related (Pearson's *R* = 0·113, *P* = 0·419). Similar results held using log_10_(*A*_*R*_) (*R* = 0·112, *P* = 0·426) or *A*_*R*_ (*R* = 0·108, *P* = 0·443) in place of 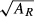. Net primary productivity and *A*_*R*_ were not significantly related (*R* = −0·184, *P* = 0·188).

**Fig 6:**
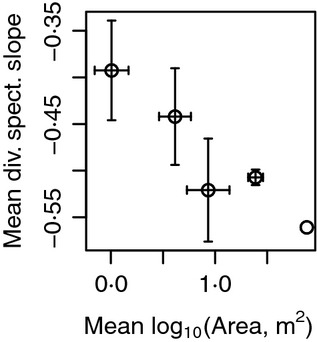
Diversity spectrum slopes for regions with linear diversity spectra as a function of spatial scale. From left to right, dots represent LME, province, basin, latitudinal band and global region means for log_10_ area (horizontal axis) and diversity spectrum slope (vertical axis). Error bars indicate standard deviations. There are no error bars for the global region point (the right point) because there was only one global region. Nonlinear regions were excluded.

**Fig 7:**
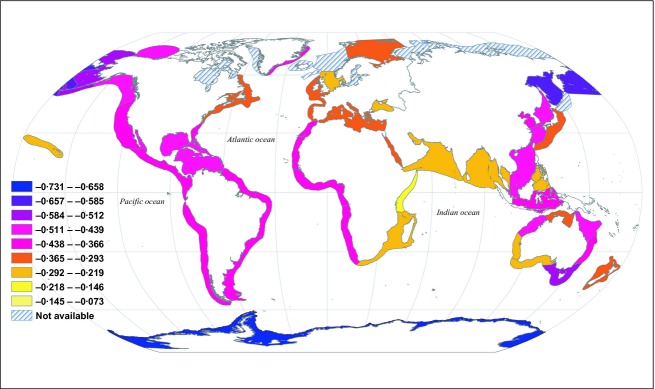
Diversity spectrum slope estimates for LMEs, excluding LMEs for which insufficient species were present to estimate the diversity spectrum or for which the diversity spectrum was nonlinear.

Predictions 4 and 5 were validated: warmer or smaller regions had shallower diversity spectrum slopes. A linear model with predictors log_10_(*A*_*R*_) and *T* explained 30·4% of the variation in slopes, and the coefficients of both predictors were significantly different from 0 (*t*-tests, *P* = 6·82 × 10^−5^ for *T*, *P* = 0·032 for log_10_(*A*_*R*_)). The *T* coefficient was positive (5·86 × 10^−3^, standard error 1·35 × 10^−3^) and the log_10_(*A*_*R*_) coefficient was negative (−0·086, standard error 0·039), as predicted by theory. Results were qualitatively the same when models were used in which LMEs were weighted by the inverses of the variances of diversity spectrum slope estimates (Appendix S10·4). The effects of area may have appeared weak in the linear model because variation in area among LMEs was modest. But area effects were clearly seen across spatial scales, by comparing diversity spectrum slopes of LMEs, provinces, basins, latitudinal bands and the global region (Fig. 6). Diversity spectrum slopes for LMEs are mapped in Fig. 7.

## Discussion

We proposed a mechanistic theory of the diversity spectrum and showed that it predicts: linearity of the diversity spectrum; its slope for the world's continental shelf seas; the range of possible slopes for smaller regions; and shallower slopes for warmer or smaller regions. To test our theory, we provided the first systematic global empirical estimates of the diversity spectrum and its geographical variation. Theoretical predictions were correct, broadly speaking, but with deviations in some details and with a few exceptional systems in the North Atlantic. Our principal qualitative conclusion is that variation in diversity with body mass can be explained, in large part, from well-known life history, predation and dispersal information and a neutral null assumption about speciation and extinction.

Our theory predicted that diversity spectra are linear, with slopes between −0·5 and −0·1; a similar range was found empirically. Slopes −0·5 and −0·1 are strikingly different. For every species in the mass category *m*_*∞,l*_ to α*m*_*∞,l*_, a community with diversity spectrum slope −0·5 has 3·2 species in the category *m*_*∞,l*_/10 to α*m*_*∞,l*_/10 and 10 species in the category *m*_*∞,l*_/100 to α*m*_*∞,l*_/100. Diversity spectrum slope −0·1 means only 1·3 and 1·6 species, respectively, in the smaller categories for each species in the largest category.

During any period of time, individuals of a given asymptotic mass compete with other individuals of the same asymptotic mass and also with other individuals of the same mass but different asymptotic mass. It is an important feature of our theory that it includes both of these types of competition. We accounted for competition among categories of asymptotic mass by incorporating the theory of Andersen & Beyer (2006). That theory provides the joint distribution *N*(*m*, *m*_*∞*_), and therefore its marginal distribution 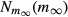, which is a complete account of the relative abundances of asymptotic mass categories and hence of the outcome of competition among categories. What Andersen & Beyer (2006) did not attempt is to describe the outcome of competition within each category, that is, into how many species is an asymptotic mass category partitioned? Our model accounts for this.

How can we intuitively understand the results that diversity spectrum slopes are steeper for larger or colder regions? Soininen, Lennon & Hillebrand (2007) argued that more dispersive assemblages should show less variation in species composition among habitats in a region (beta diversity), and provided evidence in a major meta-analysis that larger organisms show lower beta diversity. Although exceptions exist, larger organisms in the systems we study are generally more dispersive because they have longer larval durations and higher adult mobility (Siegel *et al.* 2003; Shanks, Grantham & Carr, 2003; Bradbury *et al.* 2008; see section Derivation of model component 3’); so beta diversity is expected to be a lesser contributor to total regional diversity (gamma diversity) for categories of large sizes than it is for categories of smaller sizes. But for larger regions, the contribution of beta diversity to gamma diversity is greater because spatial turnover will play a more important role in large regions (Soininen, Lennon & Hillebrand, 2007). Thus, because beta diversity contributes a bigger portion of gamma diversity for smaller size categories than for larger size categories, and because the overall importance of beta diversity is accentuated in larger regions, it is intuitively understandable that larger regions should have steeper diversity spectrum slopes. Another view of the same logic is that more large species can be found per small species in subglobal regions than in the global region because larger species have larger range sizes; a greater fraction of the global list of species in a large size category will be represented in any subglobal region compared to a smaller size category. In colder regions, there is evidence (see section Derivation of model component 3’) that dispersal is generally greater for all sizes because larval durations and life spans are longer (Gillooly *et al.* 2001; O'Connor *et al.* 2007; Bradbury *et al.* 2008). So the relative advantages large species have in ensuring their presence in more smaller regions through greater range size are less important, providing an intuitive interpretation of steeper diversity spectrum slopes in colder regions.

### Theoretical assumptions revisited

An assumption implicit in model structure is that numbers of pelagic marine species are in stochastic equilibrium, that is, not showing sustained trends over recent evolutionary time. This assumption is manifested in equations 3 and 4, which provide expected numbers of species at stochastic equilibrium. The assumption of stochastic equilibrium is consistent with available paleontological data: Benton (2009) states that ‘The equilibrium model has prevailed, among marine paleobiologists at least, for a long time…’ (see also Alroy 2008; Alroy *et al.* 2008). At regional scales, rates of migration and community assembly will primarily control the speed with which stochastic equilibrium is reached, and these rates are much higher than rates of speciation and extinction. The locations of continents and the thermohaline circulation have been approximately stable for the last 3 myr since the formation of the Isthmus of Panama. More recently, glacial cycles changed LME areas through sea level rise and fall and changed their average temperatures; but the gradient of LME temperatures has been broadly maintained through the cycles. The time since the end of the last glacial period, 10 000 years, appears long enough for any migration-related transients in regional diversity distributions to have passed, given that marine pelagic organisms generally disperse readily. LME areas, average temperatures and productivities have likely been approximately stable since then, except for recent anthropogenic climate change (Spicer & Parrish, 1986; Frakes, Probst & Ludwig, 1994; Scotese 1997).

The dispersal model (see section Derivation of model component 3’) idealized the region *R* as a disc, and migration was assumed to be non-directional (the dispersal kernel *φ* was radially symmetric). In contrast, for real shelf-sea regions, region shape and directional ocean currents can play a role in dispersal. Geographic and oceanographic details may be worth incorporating into a future model, although such a model would not be amenable to analytic solution and may require enormous computational resources. Our model shows what fraction of the variation in diversity spectrum slopes can be explained mechanistically by gross properties (temperature and area), without taking into account difficult-to-parameterize detailed information: about a third of the variation was explainable.

### Data revisited

Fish were used as a proxy for the whole community in the asymptotic mass range 1 to 1000 kg. This choice relies on the assumptions that fish have been adequately sampled in that range and that fish truly represent almost all or a consistent fraction of the diversity in that range. Mora, Tittensor & Myers (2008) estimated that about 80% of the world's marine fish diversity has been described, considering all fish species regardless of size. Species in the asymptotic mass range 1 to 1000 kg are very heavily sampled because they are more noticeable and often have economic importance. Thus, it is reasonable to expect that considerably more than 80% of the fish species in the range are known. This is enough for this study, which examines log-scale species richness patterns.

Although fish are expected to dominate marine pelagic biomass and diversity in the *m*_*∞*_ range 1 to 1000 kg (Jennings *et al.* 2008), to precisely evaluate the accuracy of this approximation would require the compilation of a large amount of data for other groups. We have instead examined the approximation by looking at the few groups other than fish that seem most likely to contribute non-negligible diversity to the range. Marine mammals were examined in Results and Table S5. Diversity patterns that could be evaluated with marine mammal data included were in even better agreement with theory. Cephalopods are another group which may appear to violate the assumption, but this was shown not to be the case in Appendix S11·2, where scyphozoans are also discussed. These analyses support as reasonable the assumption that fish adequately represent pelagic diversity over the range 1 to 1000 kg (see also section Caveats and epistemological goals below).

Our model does not explicitly address the effects of fishing and climate change on distributions of species' sizes. Fishing has caused reductions in abundances of many fish stocks (Worm *et al.* 2009) and extirpations of a few species from LME-scale regions (Dulvy, Sadovy & Reynolds, 2003; Wonham & Carlton, 2005; del Monte-Luna *et al.* 2007). Climate change has caused shifts in species ranges (Beaugrand *et al.* 2002; Perry *et al.* 2005; Sumaila *et al.* 2011). These changes have the potential to modify diversity spectra at LME scales but, on decadal timescales, rates of change in the species present in LMEs are expected to be small in relation to the total species pool. LME-scale fish species occurrence data are unlikely to have yet been markedly influenced by fishing or other anthropogenic impacts given that known extinctions comprise a small portion of diversity (Dulvy, Sadovy & Reynolds, 2003; Wonham & Carlton, 2005; del Monte-Luna *et al.* 2007). However, if impacts continue, diversity spectra may be affected and the analyses of this study can provide a baseline.

The quality of some data in the Ocean Biogeographic Information System (OBIS) has been criticized, and OBIS draws on FishBase, the source of our data (Robertson 2008). The OBIS data criticized were at the level of sightings of individuals of fish species; hence, documented errors in OBIS are at much higher resolution than the LME-level species occurrence data we use. However, many of the documented errors are large in the sense of comprising reports of fish sightings that are far outside the species actual range. We explored if the reported errors could have affected our use of the coarser data, finding this to be very unlikely (Appendix S11·3).

Maximum lengths ever recorded were used as surrogates for asymptotic lengths. We checked the reasonability of this approximation by comparing FishBase maximum lengths with maximum lengths from an independent data set, the International Game Fish Association world angling records. For 525 species for which a comparison was possible, 97·3% of species had a FishBase log_10_ maximum length value that exceeded the log_10_ angling record length minus 0·1 (Appendix S11·1). Although this is a rough check of suitability, it provides evidence that shortcomings in the data, although they may have added noise, are not sufficient to have artifactually created the prominent patterns we observed.

### Caveats and epistemological goals

Although we made all reasonable efforts to ensure that shortcomings in the data and approximations in the model did not affect conclusions, our data and model remain approximations of reality. The value of the work stands in spite of its unavoidable approximate nature because we provide a first comparison between data and a mechanistic theory explaining a newly described global-scale diversity phenomenon, the diversity spectrum. A theory including every known biological mechanism that may influence diversity is not possible and is probably not desirable. A problem-free data set of community diversity for all LMEs is not close to achievable and will probably never be available. We view our work as a first step and suggest that other researchers try to explain diversity spectra by comparing model-data agreement after formulating models with additional or alternative mechanisms and/or compiling improved data.

### Further implications and future directions

Our model makes additional predictions which could be tested if additional data were assembled. First, the model predicts that the global diversity spectrum for the asymptotic mass range *m*_*egg*_ to 1 kg should be approximately linear with slope about −0·5, as it was for the range 1 to 1000 kg. To get specific predictions for global numbers of species in various ranges of asymptotic mass, we considered a tP distribution with exponent corresponding to diversity spectrum slope −0·5 (exponent −1·5, *b* = 0·5) and with lower bound *m*_*egg*_ and upper bound 1000 kg. The distribution was normalized so that its integral from 1 to 1000 kg was 2885, the number of known species of pelagic fish with *m*_*∞*_ in that range. We then integrated this distribution from 0·1 to 1 kg to get the prediction that there are about 6400 species in the pelagic marine environment with *m*_∞_ in this range. Integrating from 0·01 to 0·1 kg provides the prediction that there are about 20 400 species with *m*_∞_ in this smaller range. Similarly, there are predicted to be about 64 400 species with *m*_∞_ in the range 0·001 to 0·01 kg. These are order-of-magnitude predictions. Predictions may be less accurate for the categories of smallest asymptotic mass, because they represent a longer extrapolation. Predictions for specific LMEs can be calculated by a similar procedure. For instance, the predicted numbers of species in the North Sea in the *m*_∞_ categories 0·1 to 1, 0·01 to 0·1 and 0·001 to 0·01 were 125, 232 and 431, respectively. Some predictions could be tested in future work if comprehensive data on crustaceans and other pelagic marine faunas with *m*_∞_ in the range *m*_*egg*_ to 1 kg were assembled. Predictions would also be straightforward regarding how diversity varies across trophic levels and regarding variation in trophic vulnerability and generality with size, and could be tested (Appendix S12).

Studies of distributions of species' body sizes for a specified taxonomic group have been much more common than approaches that consider all species in geographically delimited regions regardless of taxonomy. For instance, Hutchinson & MacArthur (1959); May (1978); Blackburn & Gaston (1994); Loder, Blackburn & Gaston (1997); Marquet *et al*. (2005) and Brown & Nicoletto (1991) have all studied clade-specific species mass distributions either theoretically, empirically or both. Our theory differs in approach by considering all taxa, although we approximated the whole community by a single clade in a mass range to test theory. Clade-specific theory must explicitly or implicitly consider physiological limits that constrain the sizes of species in that clade by virtue of their traits. For instance, mammals probably cannot be smaller than some threshold because endothermy is difficult to maintain at small sizes. Further, competition and predation between the focal clade and other clades may need to be understood but is rarely considered in clade-specific studies. An interesting subject for future research would be to theoretically explain and empirically test how clade-specific distributions combine to form whole community distributions.

We are aware of only one prior study (Reuman *et al*. 2008) that systematically examined distributions of species characteristic masses from a geographically delimited, taxonomically inclusive viewpoint. That study considered systems on much smaller spatial scales and of very different ecological types from the marine systems of this study: 149 freshwater pelagic, estuarine and soil systems. Nevertheless, Reuman *et al*. (2008) found diversity spectra that were typically (though not always) approximately linear, with a range of slopes that overlapped substantially with the range of this study. The slopes of their 129 linear systems had 2·5th and 97·5th percentiles −0·316 and −0·099, respectively. Only two systems had 99% or 95% confidence intervals not overlapping the range −0·5 to −0·1 predicted by our theory for marine systems.

It may be possible in future work to generalize our theory to ecosystems of other types. Important components of the theory appear to generalize readily. For instance, the growth model of West, Brown & Enquist (2001) is a general model. Regularity in predation behaviour, as assayed by predator-prey mass ratios, has also been documented in non-marine systems (Brose *et al.* 2006). Other components of our theory, such as the neutrality assumption, appear more difficult to generalize but may be suitable approximations for some ecosystem types. It may also be possible to judiciously modify model structure to make the neutral approximation more reasonable for other systems. We here used asymptotic mass to delimit categories within which functional equivalence of individuals is a reasonable first approximation. In systems of other types, traits in addition to or instead of body size (e.g. metabolic category, stoichiometry, leaf area, shade tolerance) may be necessary to delimit categories of approximate functional equivalence. If the relative abundance of categories can then be modelled, the same broad approach of our theory could be applied. These observations and the coincidence of our predictions with the empirical results of Reuman *et al*. (2008) suggest it may be possible to generalize our theory to freshwater, soil, estuarine and perhaps other ecosystems.

## References

[b1] Aban I, Meerschaert M, Panorska A (2006). Parameter estimation for the Truncated Pareto distribution. Journal of the American Statistical Association.

[b2] Allen AP, Gillooly JF, Savage VM, Brown JH (2006). Kinetic effects of temperature on rates of genetic divergence and speciation. Proceedings of the National Academy of Sciences.

[b3] Alroy J (2008). Dynamics of origination and extinction in the marine fossil record. Proceedings of the National Academy of Sciences.

[b4] Alroy J (2010). The shifting balance of diversity among major marine animal groups. Science.

[b5] Alroy J, Aberhan M, Bottjer DJ, Foote M, Fürsich FT, Harries PJ (2008). Phanerozoic trends in the global diversity of marine invertebrates. Science.

[b6] Andersen KH, Beyer JE (2006). Asymptotic size determines species abundance in the marine size spectrum. American Naturalist.

[b7] Barton AD, Dutkiewicz S, Flierl G, Bragg J, Follows MJ (2010). Patterns of diversity in marine phytoplankton. Science.

[b8] Beaugrand G, Edwards M, Legendre L (2010). Marine biodiversity, ecosystem functioning, and carbon cycles. Proceedings of National Academy of Sciences.

[b9] Beaugrand G, Reid P, Ibañez F, Lindley J, Edwards M (2002). Reorganization of North Atlantic marine copepod biodiversity and climate. Science.

[b10] Benton MJ (2009). The red queen and the court jester: species diversity and the role of biotic and abiotic factors through time. Science.

[b11] Blackburn TM, Gaston KJ (1994). Animal body size distributions: patterns, mechanisms and implications. Trends in Ecology and Evolution.

[b12] Bradbury IR, Laurel B, Snelgrove PVR, Bentzen P, Campana SE (2008). Global patterns in marine dispersal estimates: the influence of geography, taxonomic category, and life history. Proceedings of the Royal Society B.

[b13] Bromham L, Woolfit M, Lee M, Rambaut A (2002). Testing the relationship between morphological and molecular rates of change along phylogenies. Evolution.

[b14] Brose U, Jonsson T, Berlow EL, Warren P, Banašek-Richter C, Bersier LF (2006). Consumer-resource body-size relationships in natural food webs. Ecology.

[b15] Brown JH, Nicoletto PF (1991). Spatial scaling of species composition: body masses of North American land mammals. The American Naturalist.

[b16] Charnov E (1993). Life History Invariants: Some Explorations of Symmetry in Evolutionary Ecology.

[b17] Charnov EL, Gillooly JF (2004). Size and temperature in the evolution of fish life histories. Integrative and Comparative Biology.

[b18] Clarke A, Johnston NM (1999). Scaling of metabolic rate with body mass and temperature in teleost fish. Journal of Animal Ecology.

[b19] Clauset A, Erwin DH (2008). The evolution and distribution of species body size. Science.

[b20] Dial KP, Marzluff JM (1988). Are the smallest organisms the most diverse?. Ecology.

[b21] Duarte C, Alcaraz M (1989). To produce many small or few large eggs: a size-independent reproductive tactic of fish. Oecologia.

[b22] Dulvy NK, Sadovy Y, Reynolds J (2003). Extinction vulnerability in marine populations. Fish and Fisheries.

[b23] Etienne RS, Olff H (2004). How dispersal limitation shapes species-body size distributions in local communities. American Naturalist.

[b24] Frakes LA, Probst JL, Ludwig W (1994). Latitudinal distribution of paleotemperatures on land and sea from early Cretaceous to middle Miocene. Comptes Rendus de l'Academie des Sciences Serie II.

[b25] Froese R, Pauly DE (2006). FishBase.

[b26] Gillooly JF, Brown JH, West GB, Savage VM, Charnov EL (2001). Effects of size and temperature on metabolic rate. Science.

[b27] Gillooly JF, Allen AP, West GB, Brown JH (2005). The rate of DNA evolution: effects of body size and temperature on the molecular clock. Proceedings of the National Academy of Sciences.

[b28] Gislason H, Rice J (1998). Modelling the response of size and diversity spectra of fish assemblages to changes in exploitation. ICES Journal of Marine Science.

[b29] Gislason H, Daan N, Rice J, Pope J (2010). Size, growth, temperature and the natural mortality of marine fish. Fish and Fisheries.

[b30] Gregg WW, Casey NW (2007). Sampling biases in MODIS and SeaWiFS ocean chlorophyll data. Remote Sensing of Environment.

[b31] Hendriks AJ, Mulder C (2008). Scaling of offspring number and mass to plant and animal size: model and metanalysis. Oecologia.

[b32] Hubbell SP (2001). The Unified Neutral Theory of Biodiversity and Biogeography.

[b33] Hudson L, Isaac N, Reuman D (2013). The relationship between body mass and field metabolic rate among individual birds and mammals. Journal of Animal Ecology.

[b34] Hutchinson GE, MacArthur RH (1959). A theoretical ecological model of size distributions among species of animals. The American Naturalist.

[b35] Jennings S, Pinnegar J, Polunin N, Boon T (2001). Weak cross-species relationships between body size and trophic level belie powerful size-based trophic structuring in fish communities. Journal of Animal Ecology.

[b36] Jennings S, Mélin F, Blanchard J, Forster R, Dulvy N, Wilson R (2008). Global-scale predictions of community and ecosystem properties from simple ecological theory. Proceedings of the Royal Society B.

[b37] Kamler E (2005). Parent-egg-progeny relationships in teleost fishes: an energetic perspective. Reviews in Fish Biology and Fisheries.

[b38] Kerr S, Dickie L (2001). The Biomass Spectrum: A Predator-Prey Theory of Aquatic Production.

[b39] Loder N, Blackburn TM, Gaston KJ (1997). The slippery slope: towards an understanding of the body size frequency distribution. Oikos.

[b40] Lorenzen K (1996). The relationship between body weight and natural mortality in juvenile and adult fish: a comparison of natural ecosystems and aquaculture. Journal of Fish Biology.

[b41] MacArthur RH, Wilson EO (2001). The Theory of Island Biogeography.

[b42] Marquet PA, Quinones RA, Abades S, Labra F, Tognelli M, Arim M (2005). Scaling and power-laws in ecological systems. Journal of Experimental Biology.

[b43] Maurer BA, Brown JH (1988). Distribution of energy use and biomass among species of North American terrestrial birds. Ecology.

[b44] May RM, Waloff N, Mound LA, Waloff N (1978). The dynamics and diversity of insect faunas. Diversity of Insect Faunas.

[b45] Mélin F, Hoepffner N (2004). Global marine primary production: a satelite view.

[b46] Mittelbach G, Schemske D, Cornell H, Allen A, Brown J, Bush M (2007). Evolution and the latitudinal diversity gradient: speciation, extinction, and biogeography. Ecology Letters.

[b47] del Monte-Luna P, Lluch-Belda D, Serviere-Zaragoza E, Carmona R, Reyes-Bonilla H, Aurioles-Gamboa D, Castro-Aguirre JL (2007). Marine extinctions revisited. Fish and Fisheries.

[b48] Mora C, Tittensor DP, Myers RA (2008). The completeness of taxonomic inventories for describing the global diversity and distribution of marine fishes. Proceedings of the Royal Society of London B.

[b49] O'Connor M, Bruno J, Gaines S, Halpern B, Lester S, Kinlan B (2007). Temperature control of larval dispersal and the implications for marine ecology, evolution, and conservation. Proceedings of the National Academy of Sciences.

[b50] O'Dwyer J, Lake J, Ostling A, Savage V, Green J (2009). An integrative framework for stochastic, size-structured community assembly. Proceedings of the National Academy of Sciences.

[b51] Perry A, Low P, Ellis J, Reynolds J (2005). Climate change and distribution shifts in marine fishes. Science.

[b52] Peters RH (1983). The Ecological Implications of Body Size.

[b53] Platt T, Sathyendranath S (1988). Oceanic primary production: estimation by remote sensing at local and regional scales. Science.

[b54] Purvis A, Orme CDL, Dolphin K (2003). Why are Most Species Small-Bodied? Macroecology: Concepts and Consequences.

[b55] Reuman D, Cohen J, Mulder C (2009a). Human and environmental factors influence soil faunal abundance-mass allometry and structure. Advances in Ecological Research.

[b56] Reuman DC, Mulder C, Raffaelli D, Cohen JE (2008). Three allometric relations of population density to body mass: theoretical integration and empirical tests in 149 food webs. Ecology Letters.

[b57] Reuman D, Mulder C, Banašek-Richter C, Cattin Blandenier MF, Breure A, Den Hollander H (2009b). Allometry of body size and abundance in 166 food webs. Advances in Ecological Research.

[b58] Rice J, Gislason H (1996). Patterns of change in the size spectra of numbers and diversity of the North Sea fish assemblage, as reflected in surveys and models. ICES Journal of Marine Science.

[b59] Robertson DR (2008). Global biogeographical data bases on marine fishes: caveat emptor. Diversity and Distributions.

[b60] Rossberg A (2012). A complete analytic theory for structure and dynamics of populations and communities spanning wide ranges in body size. Advances in Ecological Research.

[b61] Rossberg A (2013). Food Webs and Biodiversity: Foundations, Models, Data.

[b62] Sarmiento J, Gruber N (2006). Ocean Biogeochemical Dynamics.

[b63] Scotese CR (1997). Continental Drift.

[b64] Shanks AL, Grantham BA, Carr MH (2003). Propagule dispersal distance and the size and spacing of marine reserves. Ecological Applications.

[b65] Sheldon RW, Sutcliff WH, Prakash A (1972). Size distribution of particles in the ocean. Limnology and Oceanography.

[b66] Sherman K, Alexander L, Gold B (1993). Large Marine Ecosystems: Stress, Mitigation and Sustainability.

[b67] Siegel DA, Kinlan BP, Gaylord B, Gaines SD (2003). Lagrangian descriptions of marine larval dispersion. Marine Ecology Progress Series.

[b68] Smith FA, Lyons SK, Ernest SKM, Jones KE, Kaufman DM, Dayan T (2003). Body mass of late quaternary mammals. Ecology.

[b69] Soininen J, Lennon J, Hillebrand H (2007). A multivariate analysis of beta diversity across organisms and environments. Ecology.

[b70] Spicer RA, Parrish JT (1986). Paleobotanical evidence for cool north polar climates in middle cretaceous (albian-cenomanian) time. Geology.

[b71] Sumaila U, Cheung W, Lam V, Pauly D, Herrick S (2011). Climate change impacts on the biophysics and economics of world fisheries. Nature Climate Change.

[b72] Thomas JA, Welch JJ, Woolfit M, Bromham L (2006). There is no universal molecular clock for invertebrates, but rate variation does not scale with body size. Proceedings of the National Academy of Sciences.

[b73] Tittensor DP, Mora C, Jetz W, Lotze HK, Ricard D, Vanden Berghe E (2010). Global patterns and predictors of marine biodiversity across taxa. Nature.

[b74] Trebilco R, Baum J, Salomon A, Dulvy N (2013). Ecosystem ecology: size-based constraints on the pyramids of life. Trends in Ecology and Evolution.

[b75] Van Valen L (1973). Body size and numbers of plants and animals. Evolution.

[b76] Ware D (1978). Bioenergetics of pelagic fish: theoretical change in swimming speed and ration with body size. Journal of the Fisheries Research Board of Canada.

[b77] West GB, Brown JH, Enquist BJ (2001). A general model for ontogenetic growth. Nature.

[b78] White EP, Enquist BJ, Green JL (2008). On estimating the exponent of power-law frequency distributions. Ecology.

[b79] White C, Phillips N, Seymour R (2006). The scaling and temperature dependence of vertebrate metabolism. Biology Letters.

[b80] Wonham MJ, Carlton JT (2005). Trends in marine biological invasions at local and regional scales: the Northeast Pacific Ocean as a model system. Biological Invasions.

[b81] Worm B, Hilborn R, Baum J, Branch T, Collie J, Costello C (2009). Rebuilding global fisheries. Science.

