# Supplementary material for: The marine diversity spectrum
Source: J Anim Ecol. 2014 Mar 3;83(4):963–79. doi: 10.1111/1365-2656.12194 (PMC4286008; doi:10.1111/1365-2656.12194)
Supplement: Data S1 — Supporting information. [file jane0083-0963-SD1.pdf]

THE MARINE DIVERSITY SPECTRUM: ONLINE APPENDIX

D.C. REUMAN, H. GISLASON, C. BARNES, F. MÉLIN, AND S. JENNINGS

Contents

List of Figures 1
List of Tables 2
S1. Units and notational conventions 2
S2. Spectra and distributions 2
S2.1. Equivalence between spectra and distributions 2
S2.2. The quadratic truncated Pareto distribution and quadratic diversity spectra 3
S3. Optimal swimming speed 3
S4. The joint distribution of individual mass and asymptotic mass 4
S4.1. Volume searched 5
S4.2. Consumption 5
S4.3. The individual size distribution 6
S4.4. Death rate by predation 6
S4.5. Individual growth 7
S4.6. The joint distribution of individual mass and asymptotic mass 8
S5. The dispersal model 9
S5.1. The neutral model dispersal parameter, $\mathfrak{m}$ 9
S5.2. The dispersal kernel width, σ_d 10
S6. The neutral model and formulas for expected numbers of species 10
S6.1. The neutral model 10
S6.2. Formulas for expected numbers of species 11
S6.3. An alternative neutral model 11
S7. Comparison between our theory and that of Etienne and Olff (2004) 12
S8. Model parameters 12
S8.1. Metabolic rates 12
S8.2. Viscosity of water 13
S8.3. Feeding kernel parameters 13
S8.4. Egg size 13
S8.5. Other parameters 14
S8.6. Adult dispersal kernel width parameter 14
S8.7. Bounds for K_1 and K_2 14
S9. Approximations 15
S9.1. Approximations involved in modeling the individual asymptotic-size distribution, J_C, and J_M 15
S9.2. Approximations involved in modeling S_M 15
S9.3. Approximations involved in modeling S_C 17
S10. Additional methods for testing model predictions 17
S10.1. Region definitions and environmental characteristics 17
S10.2. Testing for linearity 18
S10.3. Averaging procedures for computing T and P_{net} 18
S10.4. Additional linear models of diversity spectrum slope 18
S11. Data 18
S11.1. Mass-length conversion and maximum masses 18
S11.2. Cephalopods and scyphozoans 19
S11.3. Data errors documented by Robertson, 2008 19
S12. Another future direction: vulnerability and generality 20
S13. Proofs and extended computations 20
References 24

List of Figures

S1 Plot of logit($\mathfrak{m}$) against $\log_e(r_R/\sigma_d)$. 26
S2 Egg mass limit, $f(m_\infty)$. 26
S3 Accuracy of approximations used for J_C, J_M, and S_M. 26

S4 Accuracy of approximations used for S_M. 27
S5 Accuracy of approximations used for S_C: example diversity spectra. 27
S6 Accuracy of approximations used for S_C: diversity spectrum slopes. 28
S7 Map of LMEs. 29
S8 Map of provinces. 29
S9 Map basins. 30
S10 Map of latitudinal bands. 30
S11 Diversity spectrum plots for regions for which the tP was rejected. 31
S12 Diversity spectrum plots for the global region, including marine mammals. 33
S13 Diagram supporting S13.0.38. 34

List of Tables

S1 Model parameters. 35
S2 Viscosity of sea water at a range of temperatures. 36
S3 LMEs and environmental data. 37
S4 Province, basin, latitudinal band, and global-region codes and areas. 39
S5 Truncated Pareto fit results. 39
S6 Statistics for regions for which the tP was rejected. 41

S1. Units and notational conventions

Units are SI (meters, m, for distance, seconds, s, for time, kilograms, kg, for mass, and derived units based on these including Joules, J, for energy, and Watts, W, for power). However, activation energies, E, in Arrhenius factors, $e^{\frac{-E}{kT}}$ (k is Boltzmann's constant, T is temperature, see below), are in electron Volts, eV, a common practice in the metabolic literature. Notational conventions are as follows. For individual organisms: m denotes body mass (kg), m_∞ denotes asymptotic body mass (kg), l denotes length (m), a denotes surface area (m^2), and u denotes swimming speed ($\mathrm{m \cdot s^{-1}}$). For species, m_∞ is also used to denote asymptotic body mass. This does not create confusion because we assume that all individuals of a species have the same asymptotic body mass. Temperatures in formulas are in degrees Kelvin (°K). Boltzmann's constant is $k = 8.617343 \times 10^{-5}\mathrm{eV} \cdot^\circ \mathrm{K}^{-1}$, with units chosen appropriately so that Arrhenius terms are dimensionless. If x is a quantity proportional to a power of m, then the exponent is denoted e_x, so that $x \propto m^{e_x}$. The prefactor for x is denoted k_x, so that $x = k_x m^{e_x}$. Many quantities are expressed in these terms. The prefactor is treated as constant within our region of interest, R, of the ocean, but potentially taking different values for different regions. If x is proportional to an Arrhenius term then the activation energy is denoted E_x so that $x \propto e^{\frac{-E_x}{kT}}$. Prefactors for proportionalities $x \propto e^{\frac{-E_x}{kT}} m^{e_x}$ are denoted $\overline{k}_x$, so that $x = \overline{k}_x e^{\frac{-E_x}{kT}} m^{e_x}$; they are not only constant within regions, but constant among regions with respect to temperature. In one instance we use the notation $\tilde{k}_x$ to denote a prefactor that is constant within a region with respect to m, but not with respect to m_∞. We used intuitive and systematic notation or notation similar to existing well-accepted notation. For instance, neutral-theory notation is generally consistent with the neutral theory literature. We denote the neutral-theory dispersal parameter by $\mathfrak{m}$, although we also employ the notation m for individual body mass, m_∞ for asymptotic body mass, and M for the metacommunity, because notation similar to $\mathfrak{m}$ is commonly accepted in the neutral theory literature. As long as the reader is reminded that different fonts and capitalizations represent different variables, confusion will be minimized and connections with prior work made clearer by this approach.

S2. Spectra and distributions

S2.1. **Equivalence between spectra and distributions.** If x is any random variable (e.g., individual body mass, m, or individual asymptotic body mass, m_∞, or species asymptotic body mass, m_∞) and $\varphi(x)$ is its pdf, we derive here the pdf $\psi(y)$ of $y = \log_e(x)$. For x representing individual body mass or individual or species asymptotic body mass, $\log_e(\psi(y))$ is the corresponding spectrum (size spectrum for $x = m$, asymptotic-size spectrum for x equal to individual m_∞, and diversity spectrum for x equal to species m_∞). The pdf $\varphi(x)$ is characterized by the property that the probability that x is between x_1 and x_2 is

$$\int_{x_1}^{x_2} \varphi(x)dx \tag{S2.1.1}$$

for any x_1 and x_2. But this is the same as the probability that y is between $y_1 = \log_e(x_1)$ and $y_2 = \log_e(x_2)$, i.e.,

$$\int_{x_1}^{x_2} \varphi(x)dx = \int_{y_1}^{y_2} \psi(y)dy. \tag{S2.1.2}$$

Making the substitutions $y = \log_e(x)$ and $dy = dx/x$ in S2.1.2 gives

$$\int_{x_1}^{x_2} \varphi(x)dx = \int_{x_1}^{x_2} \psi(\log_e(x))dx/x. \tag{S2.1.3}$$

Because this equation holds for any x_1 and x_2, we know

$$\varphi(x) = \psi(\log_e(x))/x, \tag{S2.1.4}$$

i.e.,

$$\psi(\log_e(x)) = x\varphi(x). \tag{S2.1.5}$$

Therefore $\varphi(x) \propto x^{e_x}$ if and only if $\log_e(\psi(y))$ is linear in y of slope $e_x + 1$. Thus there is an equivalence between a power-law distribution $\varphi(x)$ and a linear spectrum $\log_e(\psi(y))$, and the slope of the spectrum is one more than the exponent of the power law. This applies equally to the individual size distribution and the size spectrum, the individual asymptotic-size distribution and the asymptotic-size spectrum, and the species asymptotic-size distribution and the diversity spectrum. The same results holds if $\log_{10}$ is used in place of $\log_e$: the pdf of $\log_{10}(x)$ is $x \log_e(10)\varphi(x)$.

S2.2. **The quadratic truncated Pareto distribution and quadratic diversity spectra.** Using the random variable m_∞, the quadratic truncated Pareto distribution is defined to have pdf of

$$\eta_\infty = \log_e(m_\infty) \tag{S2.2.1}$$

proportional to

$$\exp(-b_1\eta_\infty - b_2\eta_\infty^2) \tag{S2.2.2}$$

for η_∞ in the range $\log_e$(1kg) to $\log_e$(1000kg) and equal to 0 outside that range. The name "quadratic truncated Pareto distribution" is justified because for m_∞ distributed as a truncated Pareto, η_∞ has pdf proportional to

$$\exp(-b\eta_\infty) \tag{S2.2.3}$$

for η_∞ in the range $\log_e$(1kg) to $\log_e$(1000kg) (see section S2.1). Whereas the expression in the parentheses of S2.2.3 is linear, that in the parentheses of S2.2.2 is quadratic, so the diversity spectrum associated with a truncated Pareto species asymptotic-size distribution is linear whereas that associated with a quadratic truncated Pareto species asymptotic-size distribution is quadratic. It is easy to see by completing the square that S2.2.2 is proportional to the pdf of a normal distribution when $b_2 > 0$. So a quadratic truncated Pareto distribution includes as a special case a log-normal distribution truncated on the left and right at 1 and 1000kg respectively.

S3. Optimal swimming speed

The derivation presented here is an augmentation of a theory from [58] which has found wide application (e.g., [2, 4]). We added to the previous theory by including the effects of temperature as well as body mass.

Denote by

$$I_B = \bar{k}_{I_B} e^{\frac{-E_{I_B}}{kT}} m^{e_{I_B}} \tag{S3.0.4}$$

the resting metabolic rate (units W) of an individual marine organism of mass m at body temperature T [7, 22, 35]. By hydrodynamics theory, the power P needed to overcome the drag on a body of surface area a moving at speed u is

$$P = \frac{\rho_w a C_T u^3}{2f_p} \tag{S3.0.5}$$

where ρ_w is the density of water (kg $\cdot$ m^{-3}) and f_p is a dimensionless efficiency for converting chemical energy into propulsive power. Here

$$C_T = \frac{k_{C_T}}{R_L^w} \tag{S3.0.6}$$

is a dimensionless drag coefficient and

$$R_L = \frac{\rho_w l u}{\mu} \tag{S3.0.7}$$

is a dimensionless Reynolds number, where μ is the dynamic viscosity of water (kg $\cdot$ m^{-1} $\cdot$ s^{-1}). Assuming

$$a = k_{al} l^2 \tag{S3.0.8}$$

and

$$m = k_{ml} l^3, \tag{S3.0.9}$$

we have

$$P = \left(\frac{k_{al} k_{C_T} \rho_w^{1-w}}{2f_p}\right) \mu^w l^{2-w} u^{3-w}. \tag{S3.0.10}$$

The parameters k_{C_T} and w are dimensionless constants, empirically determined in [58].

The field metabolic rate, I_F (units W), of an individual marine organism can be approximated as

$$I_F = I_B + P. \tag{S3.0.11}$$

Following [2] and others, we assume that the energy intake per unit time (through food consumption) of a swimming individual is proportional to encountered food, which is proportional to the volume swept out by the perceptual radius of the individual. This is in turn proportional to swimming speed, u. Reference [58] computed the optimal swimming speed, u_{opt}, by optimizing the quantity u/I_F, the distance covered per unit metabolic energy spent. Solving

$$\frac{d}{du}\left(\frac{u}{I_F}\right) = \frac{1}{I_B + P} - \frac{(3-w)P}{(I_B+P)^2} = 0 \tag{S3.0.12}$$

leads to

$$I_B = (2-w)P \tag{S3.0.13}$$

which gives a unique optimal swimming speed

$$u_{opt} = \left(\frac{2k_{I_B} f_p k_{ml}^{\frac{2-w}{3}}}{(2-w)k_{al}k_{C_T}\rho_w^{1-w}}\right)^{\frac{1}{3-w}} \left(\frac{e^{\frac{-E_{I_B}}{kT}}}{\mu^w}\right)^{\frac{1}{3-w}} m^{\frac{e_{I_B} - \frac{2-w}{3}}{3-w}} \tag{S3.0.14}$$

where the second and third factors, respectively, indicate the temperature and mass dependence of u_{opt} and the first factor is a constant. The density of water ρ_w depends slightly on temperature and salinity. However, compared to the other temperature-dependent terms in S3.0.14 this variation is small for liquid-state water over realistic ranges of temperature and salinity, so ρ_w is treated as a constant.

We used an Arrhenius model of viscosity:

$$\mu = k_\mu e^{\frac{-E_\mu}{kT}}. \tag{S3.0.15}$$

See section S8.2 for validation that this model accurately describes the temperature dependence of viscosity. Substituting into S3.0.14, we get

$$u_{opt} = \overline{k}_{u_{opt}} e^{\frac{-E_{u_{opt}}}{kT}} m^{e_{u_{opt}}} \tag{S3.0.16}$$

where

$$\overline{k}_{u_{opt}} = \left(\frac{2k_{I_B} f_p k_{ml}^{\frac{2-w}{3}}}{(2-w)k_{al}k_{C_T}k_\mu^w\rho_w^{1-w}}\right)^{\frac{1}{3-w}} \tag{S3.0.17}$$

$$E_{u_{opt}} = \frac{E_{I_B} - wE_\mu}{3-w} \tag{S3.0.18}$$

$$e_{u_{opt}} = \frac{e_{I_B} - \frac{2-w}{3}}{3-w}. \tag{S3.0.19}$$

It is straightforward to use S3.0.13 and S3.0.11 to show that

$$I_F = \frac{3-w}{2-w} I_B, \tag{S3.0.20}$$

i.e.,

$$I_F = \overline{k}_{I_F} e^{\frac{-E_{I_F}}{kT}} m^{e_{I_F}} \tag{S3.0.21}$$

where

$$\overline{k}_{I_F} = \frac{3-w}{2-w}\overline{k}_{I_B}, \tag{S3.0.22}$$

$$E_{I_F} = E_{I_B} \tag{S3.0.23}$$

and

$$e_{I_F} = e_{I_B}. \tag{S3.0.24}$$

Dropping the explicit temperature terms from S3.0.4, S3.0.16, and S3.0.21, for a single region or temperature we have

$$I_B = k_{I_B} m^{e_{I_B}}, \tag{S3.0.25}$$

$$u_{opt} = k_{u_{opt}} m^{e_{u_{opt}}}, \tag{S3.0.26}$$

and

$$I_F = k_{I_F} m^{e_{I_F}}. \tag{S3.0.27}$$

Reference [35] provides empirical support for the power law dependence of S3.0.26 (see section S8.5).

S4. The joint distribution of individual mass and asymptotic mass

The theory of this section is an augmented version of the theory of reference [2]. Notation has been adapted. Additions are indicated.

S4.1. **Volume searched.** The volume searched per unit time (units $m^3 \cdot s^{-1}$) by an individual fish swimming at optimal speed is

(S4.1.1) $$v = u_{opt}\pi r_{per}^2,$$

where r_{per} is the perceptual radius in meters. Following [2], we assume

(S4.1.2) $$r_{per} = k_{r_{per}} l.$$

Reference [58] makes a similar assumption based on experimental results on visual contrast thresholds in goldfish [24]. Using S4.1.1, S4.1.2, S3.0.26 and S3.0.9, it is straightforward to show that

(S4.1.3) $$v = k_v m^{e_v}$$

where

(S4.1.4) $$e_v = e_{u_{opt}} + 2/3.$$

S4.2. **Consumption.** Let

(S4.2.1) $$N_m = k_{N_m} m^{e_{N_m}}$$

denote the individual size distribution in an area of the ocean, which has units of individuals per m^3, so that $N_m(m)dm$ is the number of individuals per m^3, regardless of taxonomy, that have body mass between m and $m + dm$. The distribution of $\omega = \log_e(m)$ is proportional to

(S4.2.2) $$\exp((e_{N_m} + 1)\omega) = m^{e_{N_m}+1},$$

as proved in section S2.1 and by [2, 47, 61] and others. The classical size spectrum (sometimes also called the abundance spectrum) is a constant plus the $\log_e$ of this,

(S4.2.3) $$(e_{N_m} + 1)\omega,$$

plotted against ω, and hence is linear of slope $e_{N_m} + 1$.

Consumption, c, of an individual organism of mass m is related to N_m because it is proportional to food encountered:

(S4.2.4) $$c = v \int_0^\infty m_p N_m(m_p)\phi(m_p, m)dm_p,$$

where

(S4.2.5) $$\phi(m_p, m) = \frac{\tau_f}{\sigma_f\sqrt{2\pi}} \exp\left(\frac{-1}{2\sigma_f^2}\left(\log_e\left(\frac{m}{\beta_f m_p}\right)\right)^2\right)$$

is a dimensionless feeding preference kernel, m_p is prey mass, β_f (dimensionless) is a preferred consumer-to-resource mass ratio, σ_f (dimensionless) determines the width of the kernel, and τ_f (dimensionless) is a parameter that controls the overall likelihood of catching and consuming encountered prey. The units of c are $kg \cdot s^{-1}$. Substituting S4.2.1 and S4.2.5 into S4.2.4 gives

(S4.2.6) $$c = v \int_0^\infty \frac{k_{N_m} m_p^{e_{N_m}+1}\tau_f}{\sigma_f\sqrt{2\pi}} \exp\left(\frac{-1}{2\sigma_f^2}\left(\log_e\left(\frac{m}{\beta_f}\right) - \log_e(m_p)\right)^2\right) dm_p.$$

Substituting $x_{pref} = \log_e\left(\frac{m}{\beta_f}\right)$ and $x = \log_e(m_p)$, $m_p = \exp(x)$, $dm_p = \exp(x)dx$ gives

(S4.2.7) $$c = \frac{k_{N_m}\tau_f}{\sigma_f\sqrt{2\pi}} v \int_0^\infty m_p^{e_{N_m}+1} \exp\left(\frac{-1}{2\sigma_f^2}\left(\log_e\left(\frac{m}{\beta_f}\right) - \log_e(m_p)\right)^2\right) dm_p$$

(S4.2.8) $$= \frac{k_{N_m}\tau_f}{\sigma_f\sqrt{2\pi}} v \int_{-\infty}^\infty \exp((e_{N_m}+1)x) \exp\left(\frac{-1}{2\sigma_f^2}(x_{pref} - x)^2\right) \exp(x)dx$$

(S4.2.9) $$= \frac{k_{N_m}\tau_f}{\sigma_f\sqrt{2\pi}} v \int_{-\infty}^\infty \exp\left(\frac{-1}{2\sigma_f^2}(x_{pref} - x)^2 + (e_{N_m}+2)x\right) dx.$$

Applying lemma S13.0.1,

(S4.2.10) $$c = \left(\frac{k_{N_m}\tau_f}{\sigma_f\sqrt{2\pi}} v\right)\left(\sigma_f\sqrt{2\pi} \exp\left(x_{pref}(e_{N_m}+2) + \frac{(e_{N_m}+2)^2\sigma_f^2}{2}\right)\right)$$

(S4.2.11) $$= k_{N_m}\tau_f k_v m^{e_v} \exp\left(x_{pref}(e_{N_m}+2) + \frac{(e_{N_m}+2)^2\sigma_f^2}{2}\right)$$

(S4.2.12) $$= \left(\frac{\tau_f k_v k_{N_m} \exp((e_{N_m}+2)^2\sigma_f^2/2)}{\beta_f^{e_{N_m}+2}}\right) m^{e_v + e_{N_m} + 2}.$$

Thus,

(S4.2.13) $$c = k_c m^{e_c},$$

where

$$k_c = \frac{\tau_f k_v k_{N_m} \exp((e_{N_m}+2)^2\sigma_f^2/2)}{\beta_f^{e_{N_m}+2}} \tag{S4.2.14}$$

and

$$e_c = e_v + e_{N_m} + 2. \tag{S4.2.15}$$

S4.3. **The individual size distribution.** Let f_e denote the efficiency of eating, units $\mathrm{J} \cdot \mathrm{kg}^{-1}$, i.e., the amount of energy obtained from a given mass of ingested food. This is the product $f_e = f_{di} f_{as}$ of an ingestion efficiency, f_{as} (percent food ingested that crosses the gut wall), and a conversion rate, f_{di}, of mass to energy by digestion. Ingestion times f_e must meet the demands of field metabolic rate at steady state, i.e.,

$$f_e c = I_F, \tag{S4.3.1}$$

where the dimensions of both sides are W. Substituting S4.2.12 and S3.0.27 into S4.3.1 gives

$$f_e \left(\frac{\tau_f k_v k_{N_m} \exp((e_{N_m}+2)^2\sigma_f^2/2)}{\beta_f^{e_{N_m}+2}} \right) m^{e_v + e_{N_m} + 2} = k_{I_F} m^{e_{I_F}}. \tag{S4.3.2}$$

Because the exponents of m in this equation must be equal, we have

$$e_{N_m} = e_{I_F} - e_v - 2. \tag{S4.3.3}$$

We also conclude from S4.3.2 that

$$k_{N_m} = \left(\frac{k_{I_F} \beta_f^{e_{N_m}+2}}{f_e \tau_f k_v \exp((e_{N_m}+2)^2\sigma_f^2/2)} \right). \tag{S4.3.4}$$

We can now also conclude from S4.2.14, S4.2.15 and S4.3.4 that

$$k_c = \left(\frac{\tau_f k_v \exp((e_{N_m}+2)^2\sigma_f^2/2)}{\beta_f^{e_{N_m}+2}} \right) \left(\frac{k_{I_F} \beta_f^{e_{N_m}+2}}{f_e \tau_f k_v \exp((e_{N_m}+2)^2\sigma_f^2/2)} \right) \tag{S4.3.5}$$

$$= \frac{k_{I_F}}{f_e}, \tag{S4.3.6}$$

and

$$e_c = e_v + e_{N_m} + 2 = e_{I_F}. \tag{S4.3.7}$$

S4.4. **Death rate by predation.** The death risk from predation for an individual of mass m is

$$d = \int_0^\infty v(m_p) N_m(m_p) \phi(m, m_p) dm_p \tag{S4.4.1}$$

$$= \int_0^\infty (k_v m_p^{e_v})(k_{N_m} m_p^{e_{N_m}}) \left(\frac{\tau_f}{\sigma_f \sqrt{2\pi}} \exp\left(\frac{-1}{2\sigma_f^2} \left(\log_e\left(\frac{m_p}{\beta_f m} \right) \right)^2 \right) \right) dm_p \tag{S4.4.2}$$

$$= \frac{k_v \tau_f}{\sigma_f \sqrt{2\pi}} k_{N_m} \int_0^\infty m_p^{e_v + e_{N_m}} \exp\left(\frac{-1}{2\sigma_f^2} (\log_e(m_p) - \log_e(\beta_f m))^2 \right) dm_p, \tag{S4.4.3}$$

where now m_p is the mass of a predator. Substituting $x_{pref} = \log_e(\beta_f m)$, $x = \log_e(m_p)$, $m_p = \exp(x)$, $dm_p = \exp(x)dx$, and $e_v + e_{N_m} = e_{I_F} - 2$ (see S4.3.3) gives

$$d = \frac{k_v \tau_f}{\sigma_f \sqrt{2\pi}} k_{N_m} \int_{-\infty}^\infty \exp((e_{I_F}-1)x) \exp\left(\frac{-1}{2\sigma_f^2} (x - x_{pref})^2 \right) dx \tag{S4.4.4}$$

$$= \frac{k_v \tau_f}{\sigma_f \sqrt{2\pi}} k_{N_m} \int_{-\infty}^\infty \exp\left(\frac{-1}{2\sigma_f^2} (x - x_{pref})^2 + (e_{I_F}-1)x \right) dx. \tag{S4.4.5}$$

By lemma S13.0.1, this equals

$$d = \frac{k_v \tau_f}{\sigma_f \sqrt{2\pi}} k_{N_m} \sigma_f \sqrt{2\pi} \exp\left(x_{pref}(e_{I_F}-1) + \frac{(e_{I_F}-1)^2\sigma_f^2}{2} \right) \tag{S4.4.6}$$

$$= k_v \tau_f k_{N_m} \beta_f^{e_{I_F}-1} m^{e_{I_F}-1} \exp((e_{I_F}-1)^2\sigma_f^2/2). \tag{S4.4.7}$$

Substituting S4.3.4 into S4.4.7 gives

$$d = \tag{S4.4.8}$$

$$k_v \tau_f \left(\frac{k_{I_F} \beta_f^{e_{N_m}+2}}{f_e \tau_f k_v \exp((e_{N_m}+2)^2\sigma_f^2/2)} \right) (\beta_f m)^{e_{I_F}-1} \exp((e_{I_F}-1)^2\sigma_f^2/2) \tag{S4.4.9}$$

$$= \left(\frac{k_{I_F} \beta_f^{e_{N_m}+e_{I_F}+1} \exp(((e_{I_F}-1)^2 - (e_{N_m}+2)^2)\sigma_f^2/2)}{f_e} \right) m^{e_{I_F}-1}. \tag{S4.4.10}$$

So

$$d = k_d m^{e_d} \tag{S4.4.11}$$

where

$$k_d = \frac{k_{I_F} \beta_f^{e_{N_m}+e_{I_F}+1} \exp(((e_{I_F}-1)^2 - (e_{N_m}+2)^2)\sigma_f^2/2)}{f_e} \tag{S4.4.12}$$

and

$$e_d = e_{I_F} - 1. \tag{S4.4.13}$$

S4.5. **Individual growth.** This section uses a von Bertalanffy equation of individual ontogenetic growth, paralleling the development of [2], but more closely following the similar and more explicit equations of [59]. That reference also supports the equations with a first-principles theory. We assume indeterminate growth because most marine organisms exhibit indeterminate growth. The starting equation of [59] is

$$I_F = \frac{m}{m_{cell}} I_{cell} + \frac{f_g}{m_{cell}} \frac{dm}{dt}, \tag{S4.5.1}$$

where m_{cell} (kg) is the average mass of a cell, I_{cell} (W) is the metabolic rate of a cell, and f_g (J) is the energy required to produce a cell. The two terms on the right side of this equation correspond, respectively, to the metabolism needed to maintain existing cells and the metabolism needed to create new cells. Because of inefficiencies, f_g will be less than $f_{di} m_{cell}$, which is the energy that would be obtained by digesting an amount of food equal to the mass of a cell. Rearranging S4.5.1 gives

$$\frac{dm}{dt} = \frac{m_{cell}}{f_g} I_F - \frac{I_{cell}}{f_g} m. \tag{S4.5.2}$$

We write

$$I_{cell} = \overline{k}_{I_{cell}} e^{\frac{-E_{I_F}}{kT}} m_{cell}^{e_{I_F}} \tag{S4.5.3}$$

and we substitute this equation and S3.0.21 into S4.5.2 to get

$$\frac{dm}{dt} = \frac{m_{cell}}{f_g} \overline{k}_{I_F} e^{\frac{-E_{I_F}}{kT}} m^{e_{I_F}} - \frac{\overline{k}_{I_{cell}} e^{\frac{-E_{I_F}}{kT}} m_{cell}^{e_{I_F}}}{f_g} m \tag{S4.5.4}$$

$$= \frac{e^{\frac{-E_{I_F}}{kT}}}{f_g} \left(m_{cell} \overline{k}_{I_F} m^{e_{I_F}} - \overline{k}_{I_{cell}} m_{cell}^{e_{I_F}} m \right). \tag{S4.5.5}$$

By the definition of asymptotic body mass,

$$\left. \frac{dm}{dt} \right|_{m=m_\infty} = 0. \tag{S4.5.6}$$

Therefore, plugging m_∞ into S4.5.5 gives

$$0 = m_{cell} \overline{k}_{I_F} m_\infty^{e_{I_F}} - \overline{k}_{I_{cell}} m_{cell}^{e_{I_F}} m_\infty, \tag{S4.5.7}$$

and therefore

$$\overline{k}_{I_{cell}} = \overline{k}_{I_F} \left(\frac{m_\infty}{m_{cell}} \right)^{e_{I_F}-1}. \tag{S4.5.8}$$

Substituting this equation into S4.5.5 gives

$$\frac{dm}{dt} = \frac{e^{\frac{-E_{I_F}}{kT}}}{f_g} \left(m_{cell} \overline{k}_{I_F} m^{e_{I_F}} - \overline{k}_{I_F} \left(\frac{m_\infty}{m_{cell}} \right)^{e_{I_F}-1} m_{cell}^{e_{I_F}} m \right) \tag{S4.5.9}$$

$$= \left(\frac{\overline{k}_{I_F} m_{cell}}{f_g} \right) e^{\frac{-E_{I_F}}{kT}} \left(m^{e_{I_F}} - m_\infty^{e_{I_F}-1} m \right) \tag{S4.5.10}$$

$$= \left(\frac{\overline{k}_{I_F} m_{cell}}{f_g} \right) e^{\frac{-E_{I_F}}{kT}} m^{e_{I_F}} \left(1 - \left(\frac{m}{m_\infty} \right)^{1-e_{I_F}} \right). \tag{S4.5.11}$$

We use the shorthand

$$g = \frac{dm}{dt}, \tag{S4.5.12}$$

so that

$$g = \overline{k}_g e^{\frac{-E_g}{kT}} m^{e_g} \left(1 - \left(\frac{m}{m_\infty} \right)^{1-e_{I_F}} \right) \tag{S4.5.13}$$

where

$$\overline{k}_g = \frac{\overline{k}_{I_F} m_{cell}}{f_g}; \tag{S4.5.14}$$

(S4.5.15) $$E_g = E_{I_F},$$

and

(S4.5.16) $$e_g = e_{I_F}.$$

Removing the explicit T dependence by including it in a parameter k_g,

(S4.5.17) $$g = k_g m^{e_g} \left(1 - \left(\frac{m}{m_\infty}\right)^{1-e_{I_F}}\right).$$

S4.6. **The joint distribution of individual mass and asymptotic mass.** Following [2], we let $N(m, m_\infty)$ be the joint distribution of individual mass and asymptotic mass. The joint distribution is constrained by the McKendrick-von Foerster equation,

(S4.6.1) $$\frac{\partial}{\partial m}(Ng) = -dN.$$

The left side of this equation represents the number of individuals growing into an infinitesimally narrow body mass category, minus the number growing out of the category. The right side of the equation represents mass-category-specific mortality. The equation holds for m in the range $f(m_\infty) \leq m \leq m_\infty$. We present evidence in section S8.4 that f can be described by

(S4.6.2) $$f(m_\infty) = \begin{cases} k_f m_\infty^{e_f} & \text{for } m_\infty \leq m_{cut} \\ m_{egg} & \text{for } m_\infty \geq m_{cut} \end{cases},$$

where m_{egg} is a fixed egg mass bigger than the eggs of all or almost all marine organisms that have asymptotic mass bigger than m_{cut}.

Substituting S4.5.17 and S4.4.11 into S4.6.1 gives

(S4.6.3) $$\frac{\partial}{\partial m}(N k_g (m^{e_{I_F}} - m m_\infty^{e_{I_F}-1})) = -k_d m^{e_{I_F}-1} N.$$

By lemma S13.0.2, the solution of this equation is

(S4.6.4) $$N = \tilde{k}_N m^{-e_{I_F}-k_{dg}} \left(1 - \left(\frac{m}{m_\infty}\right)^{1-e_{I_F}}\right)^{\frac{k_{dg}}{1-e_{I_F}}-1},$$

where $\tilde{k}_N$ is some function of m_∞ that is independent of m and

(S4.6.5) $$k_{dg} = \frac{k_d}{k_g}.$$

Since S4.6.1 applies only for m in the range $f(m_\infty) \leq m \leq m_\infty$, the solution S4.6.4 is biologically relevant only over the same range. By lemma S13.0.3,

(S4.6.6) $$\tilde{k}_N \propto m_\infty^{2e_{I_F}-e_v-3+k_{dg}}$$

for $m_\infty > m_{egg}$, so

(S4.6.7) $$N \propto m_\infty^{2e_{I_F}-e_v-3+k_{dg}} m^{-e_{I_F}-k_{dg}} \left(1 - \left(\frac{m}{m_\infty}\right)^{1-e_{I_F}}\right)^{\frac{k_{dg}}{1-e_{I_F}}-1}$$

for $m_\infty > m_{egg}$ and m in the range $f(m_\infty) \leq m \leq m_\infty$. Our derivation in this section differs from that of [2] by considering explicitly the domains over which S4.6.1 and S4.6.7 hold. This is a minor elaboration of [2] but is necessary for the individual asymptotic-size distribution computed in the main text.

Equation S4.6.7 was derived regardless of temperature and region area. The parameters in S4.6.7 also do not depend on these environmental factors. The value of e_{I_F} in S4.6.7 does not depend on the temperature of R or its metaregion because body-mass scaling of metabolic rate is independent of temperature [22]. Theory predicts the same independence for optimal swimming speed, i.e., $e_{u_{opt}}$ is independent of temperature (section S3), and hence so is e_v (see S4.1.4). By S4.6.5, S4.4.12, and S4.5.14,

(S4.6.8) $$k_{dg} = \frac{f_g \beta_f^{2e_{I_F}-e_v-1} \exp(((e_{I_F}-1)^2 - (e_{I_F}-e_v)^2)\sigma_f^2/2)}{f_{di} f_{as} m_{cell}},$$

and hence k_{dg} can be expressed in terms of e_{I_F}, e_v, β_f, σ_f, and an efficiency by which ingested food is converted into biomass. No evidence we are aware of suggests these parameters depend on temperature or area. Reference [3] provided evidence that β_f and σ_f are independent of temperature.

S5. The dispersal model

S5.1. The neutral model dispersal parameter, $\mathfrak{m}$. If a death occurs at (x_1, y_1) then the total propagule pressure from within D is proportional to

$$\int_{(x_2,y_2)\in D} \varphi((x_1,y_1),(x_2,y_2))dx_2dy_2, \tag{S5.1.1}$$

and so the average replacement pressure from inside D after a death somewhere in D is proportional to

$$\int_{(x_1,y_1)\in D}\int_{(x_2,y_2)\in D} \varphi((x_1,y_1),(x_2,y_2))dx_2dy_2dx_1dy_1. \tag{S5.1.2}$$

By similar reasoning, the average replacement pressure from outside D after a death somewhere in D is

$$\int_{(x_1,y_1)\in D}\int_{(x_2,y_2)\notin D} \varphi((x_1,y_1),(x_2,y_2))dx_2dy_2dx_1dy_1. \tag{S5.1.3}$$

We therefore set

$$\frac{\mathfrak{m}}{1-\mathfrak{m}} = \frac{\int_{(x_1,y_1)\in D}\int_{(x_2,y_2)\notin D} \varphi((x_1,y_1),(x_2,y_2))dx_2dy_2dx_1dy_1}{\int_{(x_1,y_1)\in D}\int_{(x_2,y_2)\in D} \varphi((x_1,y_1),(x_2,y_2))dx_2dy_2dx_1dy_1}. \tag{S5.1.4}$$

By lemma S13.0.4, the numerator of S5.1.4 is

$$4\pi\sigma_d^2\int_0^{2r_R} \exp\left(\frac{z^2}{-2\sigma_d^2}\right)\left(z^2\left(r_R^2-\frac{1}{4}z^2\right)\right)^{1/4} dz \tag{S5.1.5}$$

and the denominator is

$$-4\pi\sigma_d^2\int_0^{2r_R} \exp\left(\frac{z^2}{-2\sigma_d^2}\right)\left(z^2\left(r_R^2-\frac{1}{4}z^2\right)\right)^{1/4} dz + 2\pi^2\sigma_d^2 r_R^2. \tag{S5.1.6}$$

Setting $\frac{\mathfrak{m}}{1-\mathfrak{m}}$ equal to S5.1.5 over S5.1.6 and solving gives

$$\mathfrak{m} = \frac{2}{\pi r_R^2}\int_0^{2r_R} \exp\left(\frac{z^2}{-2\sigma_d^2}\right)\left(z^2\left(r_R^2-\frac{1}{4}z^2\right)\right)^{1/4} dz. \tag{S5.1.7}$$

Substituting $w = z/\sigma_d$, $z = \sigma_d w$, $dw = dz/\sigma_d$, $dz = \sigma_d dw$ gives

$$\mathfrak{m} = \frac{2\sigma_d^2}{\pi r_R^2}\int_0^{\frac{2r_R}{\sigma_d}} \exp\left(\frac{-w^2}{2}\right)\left(w^2\left(\left(\frac{r_R}{\sigma_d}\right)^2-\frac{1}{4}w^2\right)\right)^{1/4} dw. \tag{S5.1.8}$$

Because $\exp(-w^2/2)$ becomes small very quickly as w increases, we can replace

$$\left(w^2\left(\left(\frac{r_R}{\sigma_d}\right)^2-\frac{1}{4}w^2\right)\right)^{1/4} \tag{S5.1.9}$$

in S5.1.8 by any expression that approximates it well for small values of w. But

$$\left(w^2\left(\left(\frac{r_R}{\sigma_d}\right)^2-\frac{1}{4}w^2\right)\right)^{1/4} \approx \left(w^2\left(\frac{r_R}{\sigma_d}\right)^2\right)^{1/4} \tag{S5.1.10}$$

for small w and large r_R/σ_d. Therefore, we can write

$$\mathfrak{m} \approx \frac{2\sigma_d^{3/2}}{\pi r_R^{3/2}}\int_0^{\frac{2r_R}{\sigma_d}} \exp\left(\frac{-w^2}{2}\right)\sqrt{w}dw \tag{S5.1.11}$$

for large r_R/σ_d. We used this approximation for $r_R/\sigma_d > 100$ because it simplified numeric integration.

By plotting $\log_e(\frac{\mathfrak{m}}{1-\mathfrak{m}})$ against $\log_e(\frac{r_R}{\sigma_d})$, we observed that $\log_e(\frac{\mathfrak{m}}{1-\mathfrak{m}})$ asymptotically approaches the line

$$\log_e\left(\frac{\mathfrak{m}}{1-\mathfrak{m}}\right) = -\frac{3}{2}\log_e\left(\frac{r_R}{\sigma_d}\right) - 0.4215886 \tag{S5.1.12}$$

as $\log_e(\frac{r_R}{\sigma_d})$ goes to ∞, and asymptotically approaches the line

$$\log_e\left(\frac{\mathfrak{m}}{1-\mathfrak{m}}\right) = -2\log_e\left(\frac{r_R}{\sigma_d}\right) + 0.2874602 \tag{S5.1.13}$$

as $\log_e(\frac{r_R}{\sigma_d})$ goes to $-\infty$. For numeric computations, we used these linear approximations for $\log_e(\frac{r_R}{\sigma_d}) > 15$ and $\log_e(\frac{r_R}{\sigma_d}) < -7$, respectively. A better sense of how $\mathfrak{m}$ behaves can be obtained by plotting $\log_e(\frac{\mathfrak{m}}{1-\mathfrak{m}})$ against $\log_e(\frac{r_R}{\sigma_d})$ (figure S1), and that plot also helps confirm the functional form of S5.1.12 and S5.1.13.

S5.2. **The dispersal kernel width, σ_d.** Larval dispersal was considered in the main text; we here elaborate on the dependence of adult dispersal on m_∞. If adult dispersal of organisms of asymptotic mass m_∞ is proportional to the average total distance swam by such organisms in their adult lifetimes, then

$$\sigma_d \propto \int_{a_{mat}}^{\infty} p(a) u_{opt}(a) da, \tag{S5.2.1}$$

where a is age, a_{mat} is age at maturity, and $p(a)$ is the probability of survival to age a. By equation S4.5.13,

$$\frac{dm}{da} = \overline{k}_g e^{\frac{-E_g}{kT}} m^{e_g} \left(1 - \left(\frac{m}{m_\infty}\right)^{1-e_{I_F}}\right), \tag{S5.2.2}$$

so

$$da = \frac{dm}{\overline{k}_g e^{\frac{-E_g}{kT}} m^{e_g} \left(1 - \left(\frac{m}{m_\infty}\right)^{1-e_{I_F}}\right)}. \tag{S5.2.3}$$

Therefore, S5.2.1 becomes

$$\sigma_d \propto \int_{m_{mat}}^{m_\infty} p(m) e^{\frac{-E_{u_{opt}}}{kT}} m^{e_{u_{opt}}} \frac{dm}{e^{\frac{-E_{I_F}}{kT}} m^{e_{I_F}} \left(1 - \left(\frac{m}{m_\infty}\right)^{1-e_{I_F}}\right)}, \tag{S5.2.4}$$

where $p(m)$ is the probability of surviving to mass m, m_{mat} is mass at maturity, and we used S3.0.16, S4.5.15, and S4.5.16 to simplify. But

$$p(m) \propto \frac{N(m, m_\infty)}{N(m_{mat}, m_\infty)} \tag{S5.2.5}$$

$$= \left(\frac{m}{m_{mat}}\right)^{-e_{I_F} - k_{dg}} \frac{\left(1 - \left(\frac{m}{m_\infty}\right)^{1-e_{I_F}}\right)^{\frac{k_{dg}}{1-e_{I_F}} - 1}}{\left(1 - \left(\frac{m_{mat}}{m_\infty}\right)^{1-e_{I_F}}\right)^{\frac{k_{dg}}{1-e_{I_F}} - 1}}, \tag{S5.2.6}$$

so S5.2.4 simplifies to

$$\sigma_d \propto \int_{m_{mat}}^{m_\infty} \left(\frac{m}{m_{mat}}\right)^{-e_{I_F} - k_{dg}} \frac{\left(1 - \left(\frac{m}{m_\infty}\right)^{1-e_{I_F}}\right)^{\frac{k_{dg}}{1-e_{I_F}} - 1}}{\left(1 - \left(\frac{m_{mat}}{m_\infty}\right)^{1-e_{I_F}}\right)^{\frac{k_{dg}}{1-e_{I_F}} - 1}} \frac{e^{\frac{-E_{u_{opt}}}{kT}} m^{e_{u_{opt}}} dm}{e^{\frac{-E_{I_F}}{kT}} m^{e_{I_F}} \left(1 - \left(\frac{m}{m_\infty}\right)^{1-e_{I_F}}\right)} \tag{S5.2.7}$$

$$= \frac{e^{\frac{E_{I_F} - E_{u_{opt}}}{kT}} m_{mat}^{e_{I_F} + k_{dg}}}{\left(1 - \left(\frac{m_{mat}}{m_\infty}\right)^{1-e_{I_F}}\right)^{\frac{k_{dg}}{1-e_{I_F}} - 1}} \int_{m_{mat}}^{m_\infty} m^{e_{u_{opt}} - 2e_{I_F} - k_{dg}} \left(1 - \left(\frac{m}{m_\infty}\right)^{1-e_{I_F}}\right)^{\frac{k_{dg}}{1-e_{I_F}} - 2} dm. \tag{S5.2.8}$$

The temperature dependence of this expression is the same as that given in the main text (section 2.4). If parameter values are known, the m_∞ dependence of S5.2.8 can be calculated numerically for $m_\infty > m_{egg}$ and plotted, for any given assumption about how m_{mat} depends on m_∞. See section S8.6 for results of that analysis.

S6. The neutral model and formulas for expected numbers of species

We here specify the details of the neutral model and present the formulas used in the main text for expected numbers of species in the community and metacommunity.

S6.1. **The neutral model.** We used one of the several variant models which underlie the neutral theory of community dynamics introduced by Hubbell [26]. Our model was called the "Moran model" in [19]; that reference described the differences between the Moran model and other similar models (see pp. 490 and 503).

The community $C(m_\infty, \alpha m_\infty)$ and metacommunity $M(m_\infty, \alpha m_\infty)$ have sizes $J_C(m_\infty, \alpha m_\infty)$ and $J_M(m_\infty, \alpha m_\infty)$ which are fixed. All individuals in C and M have equal risk of death per unit time. Deaths happen asynchronously, whereas in the variant model called the "Wright-Fisher model" in [19], generations are discrete and all individuals die at once at the end of each time step. A vacancy created by a death in the metacommunity is immediately filled by a new individual. The new individual is of an entirely new species with probability ν, and otherwise is the offspring of an existing individual selected randomly from the metacommunity, including the individual that just died (self-replacement is allowed). A vacancy created by a death in the community is also immediately filled by a new individual. With probability $\mathfrak{m}$, the new individual is the offspring of an individual selected randomly from the metacommunity. Otherwise the new individual is the offspring of an individual selected randomly from the community (including the individual that just died, so that self-replacement is again allowed). The model allows speciation only in the metacommunity, a reasonable approximation if $J_M(m_\infty, \alpha m_\infty)$ is much bigger than $J_C(m_\infty, \alpha m_\infty)$. The only difference between the Moran model and model of reference [56], called the "Hubbell model" in [19], is that the Moran

model allows self-replacement and the Hubbell model does not. This distinction makes no substantial difference for our analysis.

S6.2. **Formulas for expected numbers of species.** Reference [20] derives the following formulas for the expected number of species in the metacommunity

$$S_M(m_\infty, \alpha m_\infty) = \theta \sum_{i=1}^{J_C} \frac{1}{\theta + i - 1} \tag{S6.2.1}$$

and community

$$S_C(m_\infty, \alpha m_\infty) \approx \theta(\Psi(\theta + I(\Psi(I + J_C) - \Psi(I))) - \Psi(\theta)), \tag{S6.2.2}$$

where Ψ is the digamma function

$$\Psi(z) = \frac{d}{dz}(\ln(\Gamma(z))). \tag{S6.2.3}$$

See [1] for properties of both Ψ and the gamma function, Γ. The approximation S6.2.2 is "extremely accurate" according to [20] (their Appendix A). Reference [20] used $\theta = 2J_M\nu$ and $I = \frac{\mathfrak{m}(J_C - 1)}{1-\mathfrak{m}}$ but they considered the Wright-Fisher model. The reasoning of [19] indicates that S6.2.1 and S6.2.2 also apply for the Moran model with

$$\theta = \frac{\nu J_M}{1 - \nu} \tag{S6.2.4}$$

and

$$I = \frac{\mathfrak{m} J_C}{1 - \mathfrak{m}}. \tag{S6.2.5}$$

Properties of the digamma function allow the simplification

$$S_M(m_\infty, \alpha m_\infty) = \theta(\Psi(\theta + J_M) - \Psi(\theta)). \tag{S6.2.6}$$

For $x > 5$ the approximation

$$\Psi(x) \approx \log_e(x) \tag{S6.2.7}$$

introduces less than 7% error; for $x > 10$ it introduces less than 2.5% error; for $x > 18$ it introduces less than 1% error and the error rate continues to decline with increasing x. We therefore apply the approximation to equation S6.2.6 to get

$$\begin{aligned} S_M &\approx \theta(\log_e(\theta + J_M) - \log_e(\theta)) && \text{(S6.2.8)} \\ &= \theta \log_e\left(1 + \frac{J_M}{\theta}\right) && \text{(S6.2.9)} \\ &= J_M \frac{\nu \log_e(1/\nu)}{1 - \nu}, && \text{(S6.2.10)} \end{aligned}$$

which is the approximation for S_M used in the main text. Applying the approximation S6.2.7 to the expression S6.2.2 for S_C gives

$$\begin{aligned} S_C &\approx \theta(\log_e(\theta + I(\log_e(I + J_C) - \log_e(I))) - \log_e(\theta)) && \text{(S6.2.11)} \\ &= \theta \log_e\left(1 + \frac{I}{\theta}\left(\log_e\left(1 + \frac{J_C}{I}\right)\right)\right) && \text{(S6.2.12)} \\ &= J_M \frac{\nu}{1-\nu} \log_e\left(1 - \frac{\mathfrak{m}\log_e(\mathfrak{m})}{1-\mathfrak{m}}\left(\frac{J_C}{J_M}\frac{1-\nu}{\nu}\right)\right), && \text{(S6.2.13)} \end{aligned}$$

which is the approximation for S_C used in the main text.

S6.3. **An alternative neutral model.** The neutral model has been criticized [31, 39, 40] for its unrealistic, point-mutation model of speciation, and for some of the consequences of this assumption, including: new species arise too frequently; many species have very low abundance; and the average species lifetime is too short compared to what is known about species lifetimes from fossil data [51]. A very recent improvement of the neutral model replaced the point-speciation assumption in the model by a "protracted speciation" concept by which incipient species must survive for a period of time before becoming full-fledged species [51]. The new model reportedly remedies many of the problems with the basic neutral model that had been raised. The augmented model has the same input as the basic neutral model (J_M, J_C, ν, $\mathfrak{m}$, where now ν is the probability that a new individual in the metacommunity is a new incipient species) but also has one additional parameter, τ, which encodes the number of generations it takes for an incipient species to become a full-fledged species, if it survives that long. We did not use the new model because an equation for the expected number of species in the community, S_C, has not been published. Once such an equation is developed, it would be interesting to see if using the protracted-speciation neutral model affects the predictions of the theory of this study. An assumption about how τ depends on m_∞ would have to be made or empirically supported.

An equation for the expected numbers of species in the metacommunity exists for the protracted-speciation neutral model,

$$S_M = J_M \frac{\nu}{1-\nu} \frac{1+\tau\nu}{\nu+\tau\nu}. \quad \text{(S6.3.1)}$$

Under the assumptions that both τ and ν are independent of m_∞, this formula indicates that at least the metaregion-level diversity spectrum predictions of our theory would be unchanged by using the protracted-speciation neutral model in place of the basic neutral model, since S_M in the new formula is still proportional to J_M.

S7. Comparison between our theory and that of Etienne and Olff (2004)

Our theoretical strategy of describing how J_C, J_M, $\mathfrak{m}$ and ν depend on $m_{\infty,l}$, and then using formulas to determine how S_C and S_M depend on $m_{\infty,l}$ is similar to and was partly inspired by the approach of Etienne and Olff [20] but differs in several respects.

First, we use m_∞ bounds to delimit communities and metacommunities instead of m bounds. In marine systems, individuals grow through many orders of magnitude, so the composition of m-delimited communities would change by individual growth as well as through births, deaths, and migration, violating neutral-model assumptions and making the neutral model inapplicable to these communities. In contrast, because the m_∞ of an individual is considered fixed at birth by its species, the categories of asymptotic mass used to delimit communities and metacommunities in our theory do not change with individual growth, and the neutral model applies.

Second, our derivations of J_C, J_M, and $\mathfrak{m}$ differ from those of [20]. Our theory focusses on marine systems, whereas the theory of [20] is not targeted to any specific ecosystem type, having been framed to try to develop general insight.

Third, reference [20] cited [16] as justification for their assumption that their speciation parameter ν_k depends on average species body mass as a power law with negative exponent, but we do not believe [16] has any direct bearing on either the ν_k parameter of [20] or the ν parameter of our study, and we have argued that ν is constant. Numbers of species in taxa of a given rank have been plotted against typical taxon body mass for various groups, usually showing a negative relationship (e.g., reference [57], in which numbers of mammalian species per family are plotted against average family body mass). Reference [16] used an alternative method to show that the most diverse taxon tended to lie toward the small end of the body mass range. These results have been interpreted to support models in which smaller species have higher rates of diversification. But rates of diversification have little bearing on ν because they differ from it in at least two ways: they are total rates, not per-recruit rates, and smaller taxa are also likely to be numerically more abundant; and they do not represent speciation directly, but rather the net effects of extinction and species establishment. Species establishment is related to speciation, but is not the same [37]. Furthermore, the relationship between taxon diversity and typical taxon body mass has been shown to be much less consistently negative when phylogenetic dependencies are considered [34].

Finally, reference [20] took a clade-specific viewpoint. Our theory is for the whole community, including all clades.

S8. Model parameters

Derivations of parameter values from prior measurements are described here. All parameter values needed for results are summarized in table S1.

S8.1. **Metabolic rates.** The values of $\bar{k}_{I_B}$, E_{I_B} and e_{I_B} were obtained by analyzing a large database of fish metabolic rates, body masses, and temperatures provided in [60]. Only E_{I_B} and e_{I_B} were needed for the results presented in the main text. Reference [60] also provides data for birds, mammals, amphibians, and reptiles, but only the fish data were used. The reference cites [5] as the source for their fish data, and [5] downloaded the data from FishBase [21]. Reference [60] provides body mass in grams, temperature in °C, and basal metabolic rate in $\text{mL} \cdot \text{O}_2 \cdot \text{hr}^{-1}$. These measurements were converted to kg, °K, and W, respectively, the latter using the conversion $1\text{W} = 0.05\text{mL} \cdot \text{O}_2 \cdot \text{s}^{-1}$ provided on p. 26 of [35]. The data set contained 1107 records from 82 species at temperatures ranging from 2°C to 35°C and body masses ranging from 3×10^{-5}kg to 9.09kg.

A linear mixed-effects model [44] was fitted using the nlme package in the R programming language. The model had response variable $\log_e(I_B)$, fixed-effects predictors $\log_e(m)$ and $\frac{1}{kT}$, and a random effect on intercept for species to take into account the pseudoreplication that comes from having multiple measurements for some species. The model had slope $e_{I_B} = 0.7982$ (standard error 0.0099), slope $-E_{I_B} = -0.5782$ (standard error 0.0219) with respect to $\frac{1}{kT}$, and mean intercept $21.2631 = \log_e(\bar{k}_{I_B})$ (standard error 0.8771). Model predictions, including the estimated random effects for species, explained 97.3% of the variation in $\log_e(I_B)$. Model predictions without the random effects explained 87.8% of the variation. See [11] for an earlier analysis of the same patterns using fewer data. The parameter e_{I_F}, needed for equation S4.6.7, was equal to e_{I_B} (see S3.0.24).

Although it is statistically less appropriate, we also examined a model with no random effects for comparison with the mixed-effects model. Many prior analyses of the mass and temperature dependence of metabolic rate have used fixed-effects models, ignoring pseudoreplication (e.g., [22]). The model had slope 0.8496 (standard error 0.0085) with respect to $\log_e(m)$, slope -0.3538 (standard error 0.0212) with respect to $\frac{1}{kT}$, and intercept 12.5761 (standard error 0.8550). These values were substantially different from those of the mixed-effects model, but in addition to its statistical inappropriateness, the fixed-effects model also had a much worse Akaike Information Criterion (AIC; see [8]) compared to the mixed-effects model: 2174.74 compared to 1099.83. The fixed-effects model explained 90.1% of the variation in $\log_e(I_B)$.

S8.2. **Viscosity of water.** Data on the viscosity of sea water at a range of temperatures were downloaded from the Chemical Hazards Response Information System of the U.S. Coastguard (`www.chrismanual.com/Intro/prop.htm`; downloaded data are reproduced in table S2) and used to derive k_μ and E_μ. Temperatures were converted to °K and viscosities to $\mathrm{kg} \cdot \mathrm{m}^{-1} \cdot \mathrm{s}^{-1}$. A linear (fixed-effects) model was fitted with response variable $\log_e(\mu)$ and predictor $\frac{1}{kT}$. The slope was $0.1781 = -E_\mu$ (standard error 0.0010) and the intercept was $-13.8738 = \log_e(k_\mu)$ (standard error 0.0405). The linear model explained 99.98% of the variation in $\log_e(\mu)$.

S8.3. **Feeding kernel parameters.** The parameter β_f is the consumer-to-resource mass ratio preferred by the consumer, which will differ from the central tendency of the realized ratio, β_r, because smaller prey are more common and therefore will be consumed more relative to how much they are preferred. A commonly used value for β_r, which we adopt here, is 1000; the central tendency of the data analyzed in [3] support this assumption. A commonly used value for β_f, which we also adopt, is 100 [2, 54]. Using S4.2.1 and S4.2.5, the realized feeding kernel is

$$\phi_r(m_p, m) = N_m(m_p)\phi(m_p, m) \tag{S8.3.1}$$

$$\propto m_p^{e_{N_m}} \exp\left(\frac{-1}{2\sigma_f^2}(x - x_{pref})^2\right), \tag{S8.3.2}$$

where m_p is prey mass, $x = \log_e(m_p)$, and $x_{pref} = \log_e\left(\frac{m}{\beta_f}\right)$. Therefore,

$$\phi_r(m_p, m) \tag{S8.3.3}$$

$$\propto \exp\left(\frac{-1}{2\sigma_f^2}(x_{pref} - x)^2 + e_{N_m} x\right) \tag{S8.3.4}$$

$$= \exp\left(\frac{-1}{2\sigma_f^2}(x^2 - 2(x_{pref} + \sigma_f^2 e_{N_m})x + x_{pref}^2)\right) \tag{S8.3.5}$$

$$= \exp\left(\frac{-1}{2\sigma_f^2}(x^2 - 2(x_{pref} + \sigma_f^2 e_{N_m})x + (x_{pref} + \sigma_f^2 e_{N_m})^2 - (x_{pref} + \sigma_f^2 e_{N_m})^2 + x_{pref}^2)\right) \tag{S8.3.6}$$

$$= \exp\left(\frac{-1}{2\sigma_f^2}(x - (x_{pref} + \sigma_f^2 e_{N_m}))^2\right) \exp\left(x_{pref}^2 - (x_{pref} + \sigma_f^2 e_{N_m})^2\right) \tag{S8.3.7}$$

$$\propto \exp\left(\frac{-1}{2\sigma_f^2}(x - (x_{pref} + \sigma_f^2 e_{N_m}))^2\right). \tag{S8.3.8}$$

But this is equal to

$$\exp\left(\frac{-1}{2\sigma_r^2}(x - x_{realized})^2\right), \tag{S8.3.9}$$

where σ_r is the width of the realized feeding kernel and $x_{realized} = \log_e\left(\frac{m}{\beta_r}\right)$. Therefore,

$$x_{pref} + \sigma_f^2 e_{N_m} = x_{realized} = \log_e\left(\frac{m}{\beta_r}\right) \tag{S8.3.10}$$

and

$$\sigma_r = \sigma_f, \tag{S8.3.11}$$

whereby

$$\log_e\left(\frac{m}{\beta_f}\right) + \sigma_f^2 e_{N_m} = \log_e\left(\frac{m}{\beta_r}\right). \tag{S8.3.12}$$

Solving for σ_f gives

$$\sigma_f = \sqrt{\frac{\log_e(\beta_f) - \log_e(\beta_r)}{e_{N_m}}}, \tag{S8.3.13}$$

which determines the value 1.0722 for σ_f and σ_r used in the model and appearing in table S1.

S8.4. **Egg size.** We here provide support for the functional form of $f(m_\infty)$ and values for the parameters m_{egg}, m_{cut}, e_f, and k_f, based on literature measurements. Data on average body size and egg size of copepod families were collated in reference [46] and plotted in figure S2, where a log-log-linear relationship can be seen. Reference [18] collated egg sizes and estimates of maximum size for 305 freshwater and marine teleost species and observed an upper bound in $\log_{10}$ fish egg diameter that increased with $\log_{10}$ fish maximal length, with slope about 0.5. This upper bound held up to fish length about $10^{1.5} = 31.6$cm $= 0.316$kg, after which a maximum egg diameter of about 5mm $= 6.5 \times 10^{-5}$kg held for marine species. These upper bounds for fish were plotted in figure S2 alongside the copepod data of [46]. The size-dependent fish upper bound is approximately consistent with that for copepods. These results support the use of $m_{egg} = 6.5 \times 10^{-5}$kg, $m_{cut} = 0.316$kg, and $e_f = 0.5$, from which one can solve for $k_f = 1.16 \times 10^{-4}$. All copepod and fish lengths were here converted to masses using S3.0.9. All egg diameters were converted to volumes by assuming spherical eggs, and then were converted to masses by assuming egg density equaled 1000kg/m^3, approximately the density of water. Elasmobranch species that bear live young will violate the limits described by $f(m_\infty)$, but the abundance and diversity of those species are low in the world's shelf seas relative to teleost and invertebrate species, so the approximation of ignoring them for quantifying $f(m_\infty)$ is acceptable.

Results from other studies provide further support for the chosen functional form of $f(m_\infty)$ and for our parameter values. Reference [28] collated egg diameter measurements of 254 marine teleosts and egg volume measurements of 105 marine teleosts and found distributions with long right tails and only a few species above the cutoff of 5mm, or equivalently $\frac{4}{3}\pi\left(\frac{5\text{mm}}{2}\right)^3 = 65.45\text{mm}^3$, that we adopted above. Reference [30] plotted egg size versus fish length for marine gobioid fishes. All recorded marine gobioids had eggs smaller than our maximum size, m_{egg}. Figure 6 of [30] shows an increase in egg sizes with increasing adult size, up to a threshold of about 10cm fish length (similar to our $m_{cut} = 31.6$cm), above which there was no dependence. Reference [25] examined gadoid fishes and found a similar pattern. Reference [23] includes a large metastudy of the relationship between adult size and egg size in a wide variety of taxa, finding that data generally supported a power law relationship with exponent that was around 0.5 but that varied somewhat by group.

S8.5. **Other parameters.** Reference [58] empirically determined $w = 0.58$ (dimensionless) from data of [6]. The value $e_{u_{opt}} = 0.1342$ then follows from S3.0.19 and the value of e_{I_B} (section S8.1). The value $e_v = 0.8009$ in turn follows from S4.1.4. Reference [35] provides not only empirical support for the power law dependence of u_{opt} on m, but also empirically finds $e_{u_{opt}} = 0.13$, supporting our theoretical result. Given the values of w, E_μ (section S8.2) and E_{I_B} (section S8.1), the value $E_{u_{opt}} = 0.2816$ follows from S3.0.18. We follow reference [59] in using $m_{cell} = 3\times10^{-12}$kg and $f_g = 2.1 \times 10^{-5}$J. The value $e_{N_m} = -2.003$ follows from S4.3.3 and the values already specified. The ingestion efficiency f_{as} is about 0.85, according to measurements of references [17, 43], and we used that value. The value of f_{di} is probably not too different from the energy content of fish as measured in a bomb calorimeter. Reference [10] measured the energy content of 10 species of Patagonian marine fish and 5 species of Patagonian marine crustacean in this way, obtaining values from 2.507×10^6 to 7.148×10^6J/kg wet mass, with mean value 4.462×10^6J/kg wet mass, so we take this mean value for f_{di}. For k_{dg}, note that all values in S4.6.8 have been determined. Inserting values and computing, we get $k_{dg} = 0.737$. We used $k_{ml} = 10$ (with units of l in m and units of m in kg).

S8.6. **Adult dispersal kernel width parameter.** Given the parameter values determined above, we plotted S5.2.8 numerically against m_∞ for two reasonable assumptions about how m_{mat} is related to m_∞. Assuming m_{mat} is a fixed fraction of m_∞, the result was always an exact power law to within computer numeric accuracy, with exponent that was always 0.336. Values of m_{mat}/m_∞ ranging from 0.01 to 0.5 were used. Assuming m_{mat} is a fixed fraction of the way from $f(m_\infty)$ to m_∞, equation S5.2.8 was still extremely close to a power law for fractions ranging from 0.01 to 0.5, with exponent varying very little: from 0.3268 for the fraction 0.01 up to 0.3364 for the fraction 0.5.

S8.7. **Bounds for K_1 and K_2.** We derive two very wide bounds for K_1 and K_2, chosen to comfortably encompass the K_1 and K_2 values for the systems we consider. Recall that K_1 is defined as the value of r_R/σ_d for the reference category m_{egg} to αm_{egg}. Because $\sigma_d \propto m_{\infty,l}^{e_{\sigma_d}}$, we know $r_R/\sigma_d \propto m_{\infty,l}^{-e_{\sigma_d}}$, and therefore

$$\frac{r_R}{\sigma_d} = K_1\left(\frac{m_{\infty,l}}{m_{egg}}\right)^{-e_{\sigma_d}}. \tag{S8.7.1}$$

Rearranging gives

$$K_1 = \frac{r_R}{\sigma_d}\left(\frac{m_{\infty,l}}{m_{egg}}\right)^{e_{\sigma_d}} \tag{S8.7.2}$$

for the asymptotic mass category with lower boundary $m_{\infty,l}$, where $m_{\infty,l}$ is arbitrary. Using $m_{\infty,l} = 1000$kg gives

$$K_1 = \frac{r_R}{\sigma_d(1000\text{kg})}\left(\frac{1000\text{kg}}{m_{egg}}\right)^{e_{\sigma_d}}. \tag{S8.7.3}$$

Certainly $\sigma_d > 10$km for organisms in the m_∞ category 1000kg to α1000kg, so

$$K_1 < \frac{r_R}{10\text{km}}\left(\frac{1000\text{kg}}{m_{egg}}\right)^{e_{\sigma_d}} \leq \frac{r_R}{10\text{km}}\left(\frac{1000\text{kg}}{m_{egg}}\right)^{0.3}. \tag{S8.7.4}$$

Using $r_R = \sqrt{A_R/\pi}$, we have

$$K_1 < \sqrt{\frac{A_R}{\pi}}\left(\frac{1}{10\text{km}}\right)\left(\frac{1000\text{kg}}{m_{egg}}\right)^{0.3}, \tag{S8.7.5}$$

and therefore

$$A_R > K_1^2\pi\left(\frac{m_{egg}}{1000\text{kg}}\right)^{0.6} 100\text{km}^2. \tag{S8.7.6}$$

By definition,

$$K_2 = \frac{J_C(1-\nu)}{J_M\nu}. \tag{S8.7.7}$$

Defining J_W to be the number of individuals in continental shelf seas worldwide with m_∞ in the range $m_{\infty,l}$ to $\alpha m_{\infty,l}$, νJ_W is the total number of speciation events per generation in the m_∞ category. Because speciation is rare and R is assumed to be large ($> 10000\text{km}^2$),

$$\nu J_W \ll J_C \approx J_C(1-\nu). \tag{S8.7.8}$$

We reasonably assume

$$\nu J_W < \frac{J_C(1-\nu)}{10}, \tag{S8.7.9}$$

so that, by rearranging,

$$10 < \frac{J_C(1-\nu)}{\nu J_W}. \tag{S8.7.10}$$

Because K_2 is greater than the right side of S8.7.10, we have

$$K_2 > 10, \tag{S8.7.11}$$

the first of our two bounds.

Given $m_{\infty,l}$ and α, let $J_{C,min}$ be a minimal value of J_C for regions of area 10000km^2, so that $J_{C,min}/10000\text{km}^2$ is a minimal population density for such regions. For larger regions,

$$J_C \geq A_R \frac{J_{C,min}}{10000\text{km}^2}, \tag{S8.7.12}$$

so

$$K_2 > \frac{J_C(1-\nu)}{\nu J_W} \geq \frac{A_R}{10000\text{km}^2}\frac{J_{C,min}(1-\nu)}{\nu J_W} > \frac{A_R}{1000\text{km}^2}, \tag{S8.7.13}$$

where the last inequality follows from S8.7.10. Using this and S8.7.6, we have

$$K_2 > K_1^2 \pi \left(\frac{m_{egg}}{1000\text{kg}}\right)^{0.6}\left(\frac{1}{10}\right) = 1.54 \times 10^{-5} K_1^2. \tag{S8.7.14}$$

This is the second of our two bounds.

S9. Approximations

S9.1. Approximations involved in modeling the individual asymptotic-size distribution, J_C, and J_M. In the main text, the individual asymptotic-size distribution was approximated by a power law in m_∞ with exponent -1.49, for m_∞ in the range m_{egg} to 1000kg. The good accuracy of this approximation is depicted in main text figure 2B. We then used the approximation

$$\begin{aligned}
J_C(m_{\infty,l}) &\propto J_M(m_{\infty,l}) && \text{(S9.1.1)}\\
&\propto \int_{m_{\infty,l}}^{\alpha m_{\infty,l}} N_{m_\infty}(m_\infty) dm_\infty && \text{(S9.1.2)}\\
&\underset{\sim}{\propto} \int_{m_{\infty,l}}^{\alpha m_{\infty,l}} m_{\infty,l}^{-1.49} dm_\infty && \text{(S9.1.3)}\\
&\propto m_{\infty,l}^{-0.49}. && \text{(S9.1.4)}
\end{aligned}$$

To determine the accuracy of this approximation, we computed S9.1.2 numerically for several values of α and compared the results to S9.1.4. The approximation was good (figure S3, black lines).

S9.2. Approximations involved in modeling S_M. In section S6.2 and in the main text we used the approximations

$$S_M \approx J_M \frac{\nu \log_e(1/\nu)}{1-\nu} \tag{S9.2.1}$$

and

$$J_M \underset{\sim}{\propto} m_{\infty,l}^{-0.49} \tag{S9.2.2}$$

to conclude that

$$S_M \underset{\sim}{\propto} m_{\infty,l}^{-0.49}, \tag{S9.2.3}$$

and therefore that the global diversity spectrum is approximately linear of slope -0.49. Recall that the global diversity spectrum is the plot of $\log_e(S_M)$ against $\log_e(m_{\infty,l})$. To examine the accuracy of the approximations used here, we log transform the expression for S_M (S6.2.6) and compute its derivative with respect to $\log_e(m_{\infty,l})$:

$$\log_e(S_M) = \log_e\left(\frac{\nu}{1-\nu}\right) + \log_e(J_M) + \log_e(\Psi(\theta + J_M) - \Psi(\theta)); \tag{S9.2.4}$$

$$\begin{aligned}
\frac{d\log_e(S_M)}{d\log_e(m_{\infty,l})} &= \left[\frac{d\log_e(S_M)}{d\log_e(J_M)}\right]\left[\frac{d\log_e(J_M)}{d\log_e(m_{\infty,l})}\right] && \text{(S9.2.5)}\\
&= \left[1 + \frac{d}{d\log_e(J_M)}\log_e(\Psi(\theta+J_M) - \Psi(\theta))\right]\left[\frac{d\log_e(J_M)}{d\log_e(m_{\infty,l})}\right]. && \text{(S9.2.6)}
\end{aligned}$$

The approximation that led to S9.2.1 was $\Psi(x) \approx \log_e(x)$ for large enough x (section S6.2). If θ is large enough,

$$\text{(S9.2.7)} \quad \Psi(\theta + J_M) - \Psi(\theta) \approx \log_e(\theta + J_M) - \log_e(\theta)$$

$$\text{(S9.2.8)} \quad = \log_e\left(1 + \frac{J_M}{\theta}\right)$$

$$\text{(S9.2.9)} \quad = -\log_e(\nu),$$

which does not depend on J_M. So

$$\text{(S9.2.10)} \quad \frac{d}{d\log_e(J_M)} \log_e(\Psi(\theta + J_M) - \Psi(\theta))$$

in S9.2.6 should be approximately 0 for large enough θ, and S_M should scale with $m_{\infty,l}$ in approximately the same way as J_M. To examine accuracy here, we compute the derivative in S9.2.10 exactly:

$$\text{(S9.2.11)} \quad \frac{d}{d\log_e(J_M)} \log_e(\Psi(\theta + J_M) - \Psi(\theta)) = \frac{\Psi_3(\theta + J_M)(\theta + J_M) - \Psi_3(\theta)\theta}{\Psi(\theta + J_M) - \Psi(\theta)},$$

where Ψ_3 is the trigamma function (the derivative of the digamma function). So

$$\text{(S9.2.12)} \quad \frac{d\log_e(S_M)}{d\log_e(m_{\infty,l})} = \left[1 + \frac{\Psi_3(\theta + J_M)(\theta + J_M) - \Psi_3(\theta)\theta}{\Psi(\theta + J_M) - \Psi(\theta)}\right] \frac{d\log_e(J_M)}{d\log_e(m_{\infty,l})}.$$

For $10^6 \leq J_M \leq 10^{26}$ and $2 \leq \theta \leq J_M/10$, which are very wide bounds,

$$\text{(S9.2.13)} \quad 1 > 1 + \frac{\Psi_3(\theta + J_M)(\theta + J_M) - \Psi_3(\theta)\theta}{\Psi(\theta + J_M) - \Psi(\theta)} > 0.978,$$

and this expression gets closer to 1 as θ or J_M gets bigger. The reasonableness of these constraints and the inequalities in S9.2.13 are supported below. We used S9.2.12 and S9.2.13 and the plot of $\log_e(J_M)$ versus $\log_e(m_{\infty,l})$ (figure S3, black lines) to provide upper bounds for the plot of $\log_e(S_M)$ versus $\log_e(m_{\infty,l})$ (figure S3, red lines). Plots were virtually the same as the plot of $\log_e(J_M)$ versus $\log_e(m_{\infty,l})$ because the expression in S9.2.13 is so close to 1. The expressions S9.2.12 and S9.2.13 and figure S3 also indicate that the plot of $\log_e(S_M)$ versus $\log_e(m_{\infty,l})$ must be nearly linear with an overall slope between 97.8% and 100% of the overall slope of $\log_e(J_M)$ versus $\log_e(m_{\infty,l})$, i.e., between about -0.48 and -0.49, to two significant figures. These slopes agree with empirical results (see main text). Thus all the approximations used to arrive at theoretical predictions on the scaling of S_M were adequate.

We now argue in support of the constraints $10^6 \leq J_M \leq 10^{26}$ and $2 \leq \theta \leq J_M/10$ and the inequalities in S9.2.13. The total area of all 63 LMEs considered is $7.58 \times 10^{13}\text{m}^2$, so the total mass of the water to 1000m depth in that area, divided by the minimum size of any of the organisms we consider (which is $f(m_{egg}) = 9.42 \times 10^{-7}$kg), is $(7.58 \times 10^{13}\text{m}^2 \times 1000\text{m} \times 1000\text{kg/m}^3)/(9.42 \times 10^{-7}\text{kg}) = 8.05 \times 10^{25}$, using 1000kg/m^3 as the approximate density of water. This is less than the upper bound used for J_M and is more than J_M could be for any of the asymptotic mass categories we consider. The lower bound for J_M, 10^6, is certainly smaller than the number of all organisms worldwide in continental shelf seas, including juveniles, of asymptotic mass between 1000kg/α and 1000kg, for reasonable values of α, and hence will also work as a lower bound for other asymptotic mass categories. Because $\theta = \frac{\nu J_M}{1-\nu}$ and ν is very small, $\theta \leq J_M/10$ (and this bound also follows easily from S8.7.9). Under the neutral model used here, the probability that two individuals, drawn at random from the metacommunity, are of the same species is $1/(1 + \nu J_M) \approx 1/(1 + \theta)$ [19]. It seems highly reasonable to assume this probability is at most 1/3 (or much less) for the categories of asymptotic mass we consider, and hence that $\theta \geq 2$. We carried out constrained minimizations and maximizations of

$$\text{(S9.2.14)} \quad 1 + \frac{\Psi_3(\theta + J_M)(\theta + J_M) - \Psi_3(\theta)\theta}{\Psi(\theta + J_M) - \Psi(\theta)},$$

beginning from over 6000 points in a grid of $\log_{10}(\theta)$ and $\log_{10}(J_M)$ values subject to the listed constraints. The bounds in S9.2.13 encompassed the results of these optimizations.

For confirmation, we adopted a second approach to quantifying the quality of the approximations used to conclude S9.2.3. To understand the scaling of S_M versus $m_{\infty,l}$, it suffices to plot

$$\text{(S9.2.15)} \quad \log_{10}(S_M) = \log_{10}\left(\frac{\nu}{1-\nu}\right) + \log_{10}(J_M) + \log_{10}(\Psi(\theta + J_M) - \Psi(\theta))$$

versus $\log_{10}(m_{\infty,l})$, ignoring the intercept of the plot. This can only be done if one is given a value for J_M in a reference category of asymptotic body mass, and values for $\nu/(1-\nu)$ and α. The advantage of using the approximation in S9.2.1 is that $\nu/(1-\nu)$ and a reference-category value for J_M are not needed to plot $\log_{10}(S_M)$ against $\log_{10}(m_{\infty,l})$ (again ignoring the intercept of the plot). Therefore, if S9.2.1 is a good approximation, then plots of S9.2.15 against $\log_{10}(m_{\infty,l})$ should be insensitive to the reference-category value for J_M and the value of $\nu/(1-\nu)$ used. We plotted S9.2.15 against $\log_{10}(m_{\infty,l})$ for a wide range of values for J_M in the reference category $m_{\infty,l} = (1000/\alpha)$kg to 1000kg and a wide range of values for $\nu/(1-\nu)$, obtaining results nearly identical to the approximation-based plot for all values used (figure S4). The range of $\nu/(1-\nu)$ used was obtained by starting with the constraint $10^{-1} \leq \theta \leq J_M/10$, which was similar to the constraint $2 \leq \theta \leq J_M/10$ used above but was even broader, and dividing it by J_M to obtain $\frac{1}{10J_M} \leq \frac{\nu}{1-\nu} \leq \frac{1}{10}$.

S9.3. **Approximations involved in modeling S_C.** In section S6.2 and in the main text we used the approximations

$$S_C \approx J_M \frac{\nu}{1-\nu} \log_e \left(1 - \frac{\mathfrak{m} \log_e(\mathfrak{m})}{1-\mathfrak{m}} \left(\frac{J_C}{J_M} \frac{1-\nu}{\nu}\right)\right) \tag{S9.3.1}$$

and

$$J_C \propto J_M \underset{\sim}{\propto} m_{\infty,l}^{-0.49} \tag{S9.3.2}$$

to conclude that the regional diversity spectrum is approximately linear with slope in a range and depending on region temperature and area. We here examine the accuracy of the approximations used. To understand the scaling of S_C versus $m_{\infty,l}$, it suffices to plot

$$\log_{10}(\theta) + \log_{10}(\Psi(\theta + I(\Psi(I + J_C) - \Psi(I))) - \Psi(\theta)) \tag{S9.3.3}$$

versus $\log_{10}(m_{\infty,l})$, ignoring the intercept of the plot (see S6.2.2). This can be done if one is given values for $\theta = \nu J_M/(1-\nu)$ in a reference category of asymptotic body mass and values for K_1, K_2, α, and e_{σ_d}. To see this, note that K_1 and e_{σ_d} determine r_R/σ_d because

$$\frac{r_R}{\sigma_d} = K_1 \left(\frac{m_{\infty,l}}{m_{egg}}\right)^{-e_{\sigma_d}}. \tag{S9.3.4}$$

This quantity in turn determines $\mathfrak{m}$ (main text and section S5.1). J_C is determined by its scaling, which is the same as that of J_M, and by its value in the reference category; the value of J_C in the reference category is determined by K_2 and the value of θ in the reference category because $K_2 = J_C/\theta$. J_C and $\mathfrak{m}$ together determine I. The advantage of using the approximations in S9.3.1 and S9.3.2 is that only K_1, K_2, and e_{σ_d} are needed to plot $\log_{10}(S_C)$ against $\log_{10}(m_{\infty,l})$ (again ignoring the intercept of the plot). Therefore, if S9.3.1 and S9.3.2 are good approximations, then plots of S9.3.3 against $\log_{10}(m_{\infty,l})$ should be insensitive to α and to the reference-category value of θ used, and should resemble the plots generated using the approximations. For the example values of K_1, K_2, and e_{σ_d} used in main text figure 3A-C, we plotted S9.3.3 against $\log_{10}(m_{\infty,l})$ for $\alpha = 1.05$ and 2 and for a wide reasonable range of values of θ in the reference category $m_{\infty,l} = (1000/\alpha)$kg to 1000kg (figure S5). Plots varied little with the values of α and reference-category θ used, and were very similar to the approximation-based plots (main text figure 3). In particular, slopes of linear approximations were nearly the same (dashed lines on figure S5).

We also showed that the results presented in main text figure 3D-F were not dependent in substance on the approximations made. For any given values of α and reference-category θ, we could compute the diversity spectrum using S9.3.3 for the three values of e_{σ_d} used in main text figure 3 and for many values of $\log_{10}(K_1)$ and $\log_{10}(K_2)$ spanning the ranges used in panels D-F of that figure. In this way, for any given α and reference-category θ, we could produce plots comparable to main text figure 3D-F, but not making use of the approximations used there. Comparison of the new plots with main text figure 3D-F indicate whether the approximations used in the main text are adequate for our purposes. We did this for $\alpha = 1.05$ and 2 and for reference-category θ equal to a wide range of values (we used the values listed in the caption of figure S5). Plots are presented for a subset of reference-category θ values in figure S6; results followed the same pattern for all reference-category θ used. Slopes were very similar to the approximation-based values in the reasonable region of K_1 and K_2 delineated by dashed lines in figure S6, indicating that the approximations used in the main text were adequate. To determine whether diversity spectra computed using S9.3.3 were always close to linear, as they were for the example plots of figure S5, we computed root mean squared errors from the best linear approximation for all diversity spectra computed. These were never bigger than 0.1039, and were usually substantially less, indicating that linear approximations were good enough to capture the most important patterns in diversity spectra. When the arrows of main text figure 4 were superimposed on the plots of figure S6, they produced the same conclusions as main text figure 4 regarding the theoretically expected trends of diversity spectrum slope across gradients of temperature and region area. Although the maximal slopes occurring on figure S6 were greater than (shallower than) the rough upper bound of -0.1 given in the main text as a likely practical limit for diversity spectrum slope of large regions, they only exceeded that value at the very edges of the reasonable bounds of K_1 and K_2, and -0.1 still seems likely to be a practical upper bound for real shelf-sea regions.

The "corners" which appear on figures S3-S5 for $\log_{10}(m_{\infty,l})$ between -1 and 0 are of minor importance for our purpose of understanding broad-scale patterns. However, they are caused by the corner assumed in the function f (figure S2), and would be rounded off and reduced even further in importance if f were replaced by a more realistic, rounded off function itself.

S10. Additional methods for testing model predictions

S10.1. **Region definitions and environmental characteristics.** LMEs are listed with their environmental characteristics (T, A_R, and net primary productivity, P_{net}) in table S3. Province names are: Antarctic, Arctic, Cape Horn, Europe, Hawaii, Indian Ocean, North Atlantic, Northeast Pacific, Northwest Atlantic, Northwest Pacific, South Africa, South Australia, Tropical East Atlantic, Tropical East Pacific, Tropical West Atlantic. Basin names are: Antarctic, Arctic, Indian Ocean, North Atlantic, North Pacific, South Atlantic, South Pacific. Latitudinal band names are: North, South, Tropical. All regions at all spatial scales are comprised of LMEs. The province, basin, and latitudinal-band membership of each LME is listed in table S3, precisely defining the provinces, basins, and latitudinal bands. Regions are mapped in figures S7-S10. Some region names are used twice at different spatial scales. For instance,

"Antarctic" can refer to an LME, a province, or a basin. Environmental information is in table S4 for provinces, basins, latitudinal bands, and for the global region consisting of all 63 LMEs.

S10.2. **Testing for linearity.** A composite test that data $m_{\infty,1},\ldots,m_{\infty,n}$ came from a distribution $\varphi(m_\infty|\Theta)$ for unknown values of the parameters Θ can be carried out with these steps: 1) obtain the maximum likelihood estimate $\hat{\Theta}$; 2) obtain the Kolmogorov-Smirnov statistic for $\varphi(m_\infty|\hat{\Theta})$ and the empirical cdf of $m_{\infty,1},\ldots,m_{\infty,n}$; 3) simulate n data points from $\varphi(m_\infty|\hat{\Theta})$, here denoted $m_{\infty,1}^{sim},\ldots,m_{\infty,n}^{sim}$; 4) obtain the maximum likelihood estimate $\hat{\Theta}^{sim}$ for the simulated data set; 5) obtain the Kolmogorov-Smirnov statistic for $\varphi(m_\infty|\hat{\Theta}^{sim})$ and the empirical cdf of $m_{\infty,1}^{sim},\ldots,m_{\infty,n}^{sim}$; 6) repeat steps 3-5 many times; 7) compare the distribution of Kolmogorov-Smirnov statistics so obtained to the statistic obtained in step 2, getting a p-value. This was done for φ a tP distribution with truncation points 1 and 1000kg, and Θ the exponent. The number of simulated data sets used was 10000.

The *empirical diversity spectrum* described in brief in section 5 of the main text is described in detail here. Recalling that the pdf of a tP distribution is $\varphi(m_\infty) = cm_\infty^{-(b+1)}$ for m_∞ between the truncation points 1 and 1000kg (where c is a normalization constant), the cdf is $\phi(m_\infty) = -\frac{c}{b}(m_\infty^{-b} - 1)$ for m_∞ between the same truncation points. Recalling that the diversity spectrum is the $\log_{10}$ of the pdf of $\eta_\infty = \log_{10}(m_\infty)$, plotted against η_∞, the diversity spectrum is $\log_{10}(\log_e(10)) + \log_{10}(c) - b\log_{10}(m_\infty)$, plotted against η_∞ (section S2.1). It is easy to show from here that the diversity spectrum is $\log_{10}(\log_e(10)(-b\phi(m_\infty) + c))$ plotted against η_∞. This transformation converts the cdf to the diversity spectrum. The transformation was applied to the empirical cdf to provide the empirical diversity spectrum.

S10.3. **Averaging procedures for computing T and P_{net}.** Data extracted from the remote sensing and primary production modeling projects listed in the main text were average sea surface temperature and primary production for each LME except the Arctic, in each month from 1997-2007, though data for some months were missing for some LMEs due to cloud and ice cover. Accompanying each monthly temperature or productivity estimate was a number from 0 to 1 representing the portion of the LME that was visible from the satellites, on which the estimate was based. For each of the 12 calendar months and each LME, means across all years for which data were available for that month and LME were computed for temperature and productivity, using means weighted by the proportions of the LME visible. In this way a single average temperature and productivity for each calendar month was computed for each LME. To get overall average temperature, T, and productivity, P_{net}, figures for each LME, we averaged over available months. For T, data were always available for all 12 months, for all 53 systems considered in the linear regressions in the main text, so averages could not have been biased by missing months. For P_{net}, there were gaps in the data set, because data were never available in the period 1997-2007 for some months in some LMEs (e.g., the Antarctic was always covered by ice in some months of its winter). P_{net} data were only used to verify that P_{net} and T were not positively related and that P_{net} and A_R were not related; we tested these assumptions for the 45 LMEs which were used in the linear models of the main text and for which P_{net} estimates were based on all 12 months, getting similar results to what is reported in the main text using all 53 LMEs.

S10.4. **Additional linear models of diversity spectrum slope.** A weighted linear model with predictors $\log_{10}(A_R)$ and T and response variable diversity spectrum slope was fitted for the 53 LMEs used in the regression of the main text. The weighting was according to the inverses of the variances of diversity spectrum slope estimates. The model coefficient for T was significantly different from 0 (t-test, $p = 0.020$) and the coefficient of $\log_{10}(A_R)$ was close to significantly different from 0 (t-test, $p = 0.075$). The T slope was positive and the $\log_{10}(A_R)$ slope was negative, as predicted by theory.

S11. Data

S11.1. **Mass-length conversion and maximum masses.** FishBase provides data on the maximum length ever observed for fish species, but maximum mass data were needed for comparison to theory. Mass versus length regression relationships for individuals of a species have been determined for many species (e.g., many are compiled in [9]), however these are not appropriate for converting maximum lengths to maximum masses for our application for two reasons. First, published relationships are intraspecific regressions, describing relationships between lengths and masses of individuals of a species. Plugging maximum length ever observed into such relationships to obtain maximum mass creates extrapolation errors. These errors may not be large, since R^2 for mass-length regressions are typically high, but they would inevitably be present, and would be worse for regressions based on fewer data. Second, and more importantly, only a minority of the fish species in our analysis have published mass-length regressions. Although there are many regressions published, there are many more species in our analysis. For instance, in any single LME, at most 57.8% of species in weight classes from $1 - 1000$kg are included in the large compilation of [9] (608 species) and the median among all LMEs is 20.1% included.

Instead we carried out an interspecific regression between maximum masses and maximum lengths. Maximum mass-maximum length pairs for species were taken from the International Game Fish Association (IGFA) website (`http://www.igfa.org/`, accessed 30 July 2013), which provides both mass and length data for world-record fish caught by angling. Freshwater fish were excluded. Reef-associated fish were excluded. We extracted 525 species records. Accepting these measurements as surrogates of asymptotic or maximum mass and length, we performed a regression of $\log_{10}$(mass, kg) versus $\log_{10}$(length, m), obtaining slope 2.541 (95% confidence intervals 2.459 to 2.623), intercept 1.038 (95% confidence intervals 1.014 to 1.061), and R^2 0.877. We also tried using the relationship $m_\infty = 10l_\infty^3$, which is a standard and oft-used, though rough, approximation for converting lengths to masses. All results of

the study were substantially the same with either regression, indicating that results are insensitive to the particular regression used.

In using maximum masses recorded as surrogates for asymptotic mass, we made a further approximation. Estimates of maximum length reported by FishBase are dependent on the number of individuals per species that were available. This, in turn, is variable among species. We compared $\log_{10}$ maximum lengths derived from FishBase to $\log_{10}$ length world record information from the IGFA. For 82.4% of the 525 species for which comparisons could be made, FishBase lengths were greater, and for 97.3% of records, FishBase $\log_{10}$ lengths were more than IGFA $\log_{10}$ lengths minus 0.1. These results corroborate the use of the FishBase data as reasonable approximate surrogates for our use of studying distributions of $\log_{10}$ asymptotic mass.

S11.2. **Cephalopods and scyphozoans.** Cephalopods also contribute to the diversity of animals in the size range considered in this study, but the data available suggest they make a small contribution to diversity. A global inventory of the body sizes and distributions of all cephalopods was not available, but total species richness of cephalopods was reported to be < 650 species [33] or, more recently, approximately 700 species (R.E. Young, K.M. Mangold and M. Vecchione, unpublished data). This compares with approximately 15000 species of marine fishes [32]. For the LMEs of the Arctic, Atlantic and Antarctic, the body sizes and distributions of all cephalopods can be assessed because references [49, 50] provide information on maximum body size (mantle length), distribution and diversity. Reference [49] described larger species of cephalopods that were known from, or expected to be taken in, fishery catches. To estimate species richness by LME, each species was assigned to LMEs based on its distribution map. Any species with a mantle length > 30cm was assumed to attain a weight of at least 1kg [50]. Among Atlantic and Arctic Ocean LMEs, cephalopod diversity was typically around 5% of fish diversity for species > 1kg asymptotic mass. The LME where the contribution of cephalopods was greatest was the Antarctic (about 10% of fish diversity in the range $1 - 1000$kg). This gives us some confidence that cephalopod diversity accounts for a consistently small proportion of fish diversity in the asymptotic mass range 1 to 1000kg; omitting this small proportion will not substantially affect the log-scale patterns we examined.

Another group that contributes to the asymptotic mass range considered would be scyphozoans. According to [15], there are approximately 200 morphospecies of scyphozoans, but molecular evidence suggests that this number should probably be doubled. Many of these species would never reach a mass above 1kg, but even if they all did, scyphozoans would represent only a tiny fraction of the species richness of marine pelagic animals with $m_\infty > 1$kg. Most scyphozoans entering the range 1 to 1000kg will be at the small end of this range, where fish are also most diverse, hence their effect on diversity spectra will be minimal.

S11.3. **Data errors documented by Robertson, 2008.** Reference [48] assessed the accuracy of OBIS location records for species of shallow-water shore fishes from the greater Caribbean region. The author of that reference examined the first species, in alphabetical order, in each genus of greater Caribbean shore fish, comparing OBIS location records with published range maps for those species. The author reported that large fractions of species examined had "large scale errors". But for almost all species for which errors were documented, only a small fraction of the location points associated with the species are indicated by [48] as being erroneous. So whereas errors were large scale in the sense that individual sightings were sometimes erroneously placed very far outside species ranges, and errors were common in the sense that many species had at least one individual-sighting record that was erroneous, raw error rates for individual-sighting records were not so common. Could a few errors in individual-sighting records per species have resulting in errors in the much coarser LME-level species occurrence data we used? We examined this question, finding this very unlikely. To examine the nature of the errors documented in [48] and their possible effects on the coarser data we used, we re-examined the first species for which errors were documented of the first five families examined in reference [48] for which errors were documented.

The species *Ginglymostoma cirratum* in the family Ginglymostomidae was documented by [48] as having 264 location points, of which 5 (1.9%) appeared clearly wrong to the author of that study. The erroneous location information documented in [48] is "S. France for Nigeria" and "Offshore Bahamas for Bimini" where this notation is explained to indicate that a site named Nigeria in primary sources was given GIS coordinates for the South of France. But the only LMEs bordering or close to the South of France are the Mediterranean Sea, the Celtic-Biscay Shelf, and the Iberian Coastal LME, none of which are among the LMEs listed for *Ginglymostoma cirratum* by FishBase. This indicates that errors of mis-locating a single sighting or a few sightings to the South of France were not incorporated in the LME-level distribution information provided by FishBase for this species. An error that mistakenly located a sighting in Bimini near the offshore Bahamas will also not have affected our LME-level data since both locations are in the Caribbean Sea LME.

The species *Mustelus canis* in the family Triakidae was listed by reference [48] as having 493 location points, of which 2 (0.41%) appeared clearly wrong to the author of that study. The error location information is given as "Cape Verde, Mauritania". The only LMEs close to those locations are the Canary Current and the Guinea Current, neither of which are listed by FishBase as among the LMEs overlapping the range of *Mustelus canis*. So any mis-location error of single sightings to the Cape Verde, Mauritania area was not incorporated in the LME-level distribution information provided by FishBase.

The species *Carcharhinus acronotus* of the family Carcharhinidae was documented as having 19 location points, of which 3 (15.8%) were documented as wrong by reference [48]. Error location information given was "TEP; TEP for Tobago; TEP for Colombia", where TEP stands for tropical Eastern Pacific. No LMEs in the Pacific Ocean basin

were listed by FishBase as among the LMEs overlapping the range of *Carcharhinus acronotus*, so any mis-location error of single sightings was not incorporated in the LME-level data of FishBase.

The species *Sphyrna lewini* of the family Sphyrnidae was documented as having 1106 location points, of which 4 (0.4%) were considered wrong by reference [48]. Error location information is given as "S France for Nigeria". But errors in the geo-referencing of a few points of this species are not important because the species is distributed worldwide in tropical and warm temperate waters. In particular, the Iberian Coastal and Mediterranean Sea LMEs, which abut the South of France, are already known to contain the species [14, 38], and were listed as among the LMEs containing the species by FishBase.

The species *Cirrhigaleus asper* of the family Squalidae was listed by reference [48] as having 22 location points, of which 1 (4.5%) was considered wrong. Location data were "W Africa for IO" where IO is the Indian Ocean. But no LMEs on the west side of Africa are listed as overlapping the range of the species in FishBase, so single-sighting mis-location errors have again not affected LME-level data.

Robertson characterizes the errors he describes as gross in scale and widespread in the database [48]. He offers many summary statistics to support this viewpoint (e.g., over 1/3 of the species he examined had errors and over 2/3 of the families he examined had errors). However, only a very small fraction of the actual location data points examined in reference [48] were designated as erroneous. Therefore, the errors reported will only be important for uses of the data for which one or a few errors per species spoil the data for the species. Our checks above indicate that our use of the data does not have that weakness; in all cases examined, errors did not propagate to the LME level occurrence data we used. We do not intend to say that either the FishBase or OBIS data sets are perfect (they are not), merely that the errors catalogued do not affect the analysis we carried out.

S12. Another future direction: vulnerability and generality

As mentioned in the introduction, our theoretical approach, which accounts for both body size and taxonomic identity, helps unify traditionally distinct species- and size-based approaches. Marine ecologists have traditionally emphasized the importance of body size over taxonomic identity, and terrestrial ecologists have traditionally emphasized taxonomic identity over body size. These traditions, while not universal, have been dominant enough to be reflected even in the data structures most commonly used: the size spectrum by marine ecologists, and the food web directed graph by freshwater and terrestrial ecologists. More integrated approaches have been emerging in the last decade, in which individuals are identified to species and also described by their functional role, often summarized by their body size (e.g., [27, 12, 13, 47, 45, 2, 41, 42, 55]). Our work extends this integrated approach and will help allow questions which have traditionally been asked in a food web (respectively, size spectrum) context to be transferred to a size spectrum (respectively, food web) context. For instance, the numbers of prey species that each predator species exploits (called its generality) and the numbers of predator species that each prey species is exploited by (called its vulnerability) are standard data quantified by a food web directed graph, but not directly encapsulated in a size spectrum approach. Under our model framework, it should be possible to derive equivalent concepts of generality and vulnerability. Since predator-prey mass ratios are relatively constant in marine environments [3], and since we showed that there are fewer large species per small species in colder ecosystems, we might infer, for instance, that each predator has a larger number of potential prey species in colder ecosystems compared to warmer ones. However, the simple counts of numbers of species used to answer generality/vulnerability questions in the food web context will need to be replaced by more nuanced notions that account for the degree to which a trophic relationship exists and the ontogenetic stages typically involved. This is because for any two species x and y in a marine system, and under the assumption of size-structured predation (realistic in a pelagic environment and adopted by our model; Appendix S4.2 and S8.3), some individuals of y will eat some individuals of x as long as the ranges $m_{egg,x}$ to $m_{\infty,x}$ and $m_{larvae,y}/\beta$ to $m_{\infty,y}/\beta$ overlap (here $m_{egg,x}$ is the size of the eggs of x, $m_{larvae,y}$ is the size of the larvae of y, and β is a predator-to-prey mass ratio). The "principle predators" of x will be those y for which these ranges overlap maximally: the largest possible range of ontogenetic growth stages of x are eaten by a corresponding stage of growth of the principle predators of x. But some stages of x are eaten by other, non-principle predators for which the ranges above overlap partially. Total consumption rates and affected growth stages of x depend on which parts of the ranges overlap, and by how much. The ratio of the numbers of species in a category of asymptotic mass to the numbers of principle predators of these species can be calculated straightforwardly from our theory at any spatial scale for which the diversity spectrum slope is known. Ratios involving non-principle predators could also be calculated in future work. Gut contents analyses and other empirical compilations of prey species for marine predators could be used to test predictions.

S13. Proofs and extended computations

Lemma S13.0.1.

$$\int_{-\infty}^{\infty} \exp\left(\frac{-1}{2\sigma^2}(x-A)^2 + Bx\right) dx = \sigma\sqrt{2\pi}\exp\left(AB + \frac{B^2\sigma^2}{2}\right) \tag{S13.0.1}$$

Proof. We proceed by completing the square:

$$\frac{-1}{2\sigma^2}(x-A)^2+Bx = \frac{-1}{2\sigma^2}(x^2-2(A+B\sigma^2)x+A^2) \tag{S13.0.2}$$

$$= \frac{-1}{2\sigma^2}(x^2-2(A+B\sigma^2)x+(A+B\sigma^2)^2-(A+B\sigma^2)^2+A^2) \tag{S13.0.3}$$

$$= \frac{-1}{2\sigma^2}((x-(A+B\sigma^2))^2-2AB\sigma^2-B^2\sigma^4) \tag{S13.0.4}$$

$$= \frac{-1}{2\sigma^2}(x-(A+B\sigma^2))^2+AB+\frac{B^2\sigma^2}{2}. \tag{S13.0.5}$$

So

$$\int_{-\infty}^{\infty}\exp\left(\tfrac{-1}{2\sigma^2}(x-A)^2+Bx\right)dx \tag{S13.0.6}$$

$$= \exp\left(AB+\tfrac{B^2\sigma^2}{2}\right)\int_{-\infty}^{\infty}\exp\left(\tfrac{-1}{2\sigma^2}(x-(A+B\sigma^2))^2\right)dx \tag{S13.0.7}$$

$$= \sqrt{2\pi}\sigma\exp\left(AB+\tfrac{B^2\sigma^2}{2}\right), \tag{S13.0.8}$$

where the last equality follows because $\int_{-\infty}^{\infty}\exp\left(\frac{-1}{2\sigma^2}(x-(A+B\sigma^2))^2\right)dx$ is $\sqrt{2\pi}\sigma$ times the density function of a normal distribution. □

Lemma S13.0.2. *The solution of*

$$\frac{\partial}{\partial m}(Nk_g(m^{e_{I_F}}-mm_\infty^{e_{I_F}-1}))=-k_dm^{e_{I_F}-1}N \tag{S13.0.9}$$

is

$$N=\tilde{k}_Nm^{-e_{I_F}-k_{dg}}\left(1-\left(\frac{m}{m_\infty}\right)^{1-e_{I_F}}\right)^{\frac{k_{dg}}{1-e_{I_F}}-1}, \tag{S13.0.10}$$

where $\tilde{k}_N$ is independent of m and $k_{dg}=\frac{k_d}{k_g}$.

Proof. It suffices to substitute S13.0.10 into S13.0.9 to verify equality. After substituting, the left side of S13.0.9 is

$$\tfrac{\partial}{\partial m}(Nk_g(m^{e_{I_F}}-mm_\infty^{e_{I_F}-1})) \tag{S13.0.11}$$

$$= \tilde{k}_Nk_g\tfrac{\partial}{\partial m}\left[m^{-e_{I_F}-k_{dg}}\left(1-\left(\tfrac{m}{m_\infty}\right)^{1-e_{I_F}}\right)^{\frac{k_{dg}}{1-e_{I_F}}-1}(m^{e_{I_F}}-mm_\infty^{e_{I_F}-1})\right] \tag{S13.0.12}$$

$$= \tilde{k}_Nk_g\tfrac{\partial}{\partial m}\left[m^{-k_{dg}}\left(1-\left(\tfrac{m}{m_\infty}\right)^{1-e_{I_F}}\right)^{\frac{k_{dg}}{1-e_{I_F}}-1}\left(1-\left(\tfrac{m}{m_\infty}\right)^{1-e_{I_F}}\right)\right] \tag{S13.0.13}$$

$$= \tilde{k}_Nk_g\tfrac{\partial}{\partial m}\left[m^{-k_{dg}}\left(1-\left(\tfrac{m}{m_\infty}\right)^{1-e_{I_F}}\right)^{\frac{k_{dg}}{1-e_{I_F}}}\right] \tag{S13.0.14}$$

$$= \tilde{k}_Nk_g\tfrac{\partial}{\partial m}\left[\left(m^{e_{I_F}-1}-m_\infty^{e_{I_F}-1}\right)^{\frac{k_{dg}}{1-e_{I_F}}}\right] \tag{S13.0.15}$$

$$= \tfrac{\tilde{k}_Nk_gk_{dg}}{1-e_{I_F}}\left(m^{e_{I_F}-1}-m_\infty^{e_{I_F}-1}\right)^{\frac{k_{dg}}{1-e_{I_F}}-1}(e_{I_F}-1)m^{e_{I_F}-2} \tag{S13.0.16}$$

$$= -\tilde{k}_Nk_dm^{e_{I_F}-2}\left(m^{e_{I_F}-1}-m_\infty^{e_{I_F}-1}\right)^{\frac{k_{dg}}{1-e_{I_F}}-1} \tag{S13.0.17}$$

$$= -\tilde{k}_Nk_dm^{-k_{dg}-1}\left(1-\left(\tfrac{m}{m_\infty}\right)^{1-e_{I_F}}\right)^{\frac{k_{dg}}{1-e_{I_F}}-1}. \tag{S13.0.18}$$

After substituting S13.0.10 into S13.0.9, the right side of S13.0.9 is

$$-k_dm^{e_{I_F}-1}N \tag{S13.0.19}$$

$$= -\tilde{k}_Nk_dm^{e_{I_F}-1}m^{-e_{I_F}-k_{dg}}\left(1-\left(\tfrac{m}{m_\infty}\right)^{1-e_{I_F}}\right)^{\frac{k_{dg}}{1-e_{I_F}}-1} \tag{S13.0.20}$$

$$= -\tilde{k}_Nk_dm^{-k_{dg}-1}\left(1-\left(\tfrac{m}{m_\infty}\right)^{1-e_{I_F}}\right)^{\frac{k_{dg}}{1-e_{I_F}}-1}, \tag{S13.0.21}$$

which is the same as S13.0.18, proving the lemma. □

Lemma S13.0.3. *For the prefactor $\tilde{k}_N$ of lemma S13.0.2,*

$$\tilde{k}_N \propto m_\infty^{2e_{I_F}-e_v-3+k_{dg}} \tag{S13.0.22}$$

for $m_\infty > m_{\text{egg}}$.

Proof. We apply the proof of [2] in the domain $m \geq m_{egg}$. We know

$$N_m(m) = \int_m^\infty N(m, m_\infty) dm_\infty \tag{S13.0.23}$$

for m in this domain. So

$$k_{N_m} m^{e_{N_m}} = \int_m^\infty \tilde{k}_N(m_\infty) m^{-e_{I_F}-k_{dg}} \left(1 - \left(\frac{m}{m_\infty}\right)^{1-e_{I_F}}\right)^{\frac{k_{dg}}{1-e_{I_F}}-1} dm_\infty, \tag{S13.0.24}$$

by S4.2.1, S4.3.3 and S13.0.10. Letting $x = \frac{m}{m_\infty}$,

$$k_{N_m} m^{e_{N_m}} = \int_0^1 \tilde{k}_N\left(\frac{m}{x}\right) m^{-e_{I_F}-k_{dg}} \left(\frac{m}{x^2}\right) \left(1 - x^{1-e_{I_F}}\right)^{\frac{k_{dg}}{1-e_{I_F}}-1} dx, \tag{S13.0.25}$$

and therefore

$$k_{N_m} m^{e_{N_m}+e_{I_F}+k_{dg}} = \int_0^1 \tilde{k}_N\left(\frac{m}{x}\right) \left(\frac{m}{x^2}\right) \left(1 - x^{1-e_{I_F}}\right)^{\frac{k_{dg}}{1-e_{I_F}}-1} dx. \tag{S13.0.26}$$

We define $\tilde{\kappa}_N(\frac{m}{x})$ implicitly by

$$\tilde{k}_N\left(\frac{m}{x}\right) = \left(\frac{m}{x}\right)^{e_{N_m}+e_{I_F}+k_{dg}-1} \tilde{\kappa}_N\left(\frac{m}{x}\right), \tag{S13.0.27}$$

so that

$$k_{N_m} m^{e_{N_m}+e_{I_F}+k_{dg}} = \int_0^1 \left(\frac{m}{x}\right)^{e_{N_m}+e_{I_F}+k_{dg}-1} \tilde{\kappa}_N\left(\frac{m}{x}\right) \left(\frac{m}{x^2}\right) \left(1 - x^{1-e_{I_F}}\right)^{\frac{k_{dg}}{1-e_{I_F}}-1} dx. \tag{S13.0.28}$$

and

$$k_{N_m} = \int_0^1 \tilde{\kappa}_N\left(\frac{m}{x}\right) x^{-e_{N_m}-e_{I_F}-k_{dg}-1} \left(1 - x^{1-e_{I_F}}\right)^{\frac{k_{dg}}{1-e_{I_F}}-1} dx. \tag{S13.0.29}$$

Since S13.0.29 holds for all $m \geq m_{egg}$, and the left side of that equation is constant, the right side must also be constant, so $\tilde{\kappa}_N$ must be a constant, proving the lemma by S13.0.27 and S4.3.3. □

Lemma S13.0.4. *If*

$$\varphi((x_1, y_1), (x_2, y_2)) = \exp\left(\frac{(x_1 - x_2)^2 + (y_1 - y_2)^2}{-2\sigma_d^2}\right), \tag{S13.0.30}$$

then

$$\int_{(x_1,y_1)\in D} \int_{(x_2,y_2)\notin D} \varphi((x_1, y_1), (x_2, y_2)) dx_2 dy_2 dx_1 dy_1 \tag{S13.0.31}$$

$$= 4\pi\sigma_d^2 \int_0^{2r_{LME}} \exp\left(\frac{z^2}{-2\sigma_d^2}\right) \left(z^2 \left(r_{LME}^2 - \tfrac{1}{4}z^2\right)\right)^{1/4} dz \tag{S13.0.32}$$

and

$$\int_{(x_1,y_1)\in D} \int_{(x_2,y_2)\in D} \varphi((x_1, y_1), (x_2, y_2)) dx_2 dy_2 dx_1 dy_1 \tag{S13.0.33}$$

$$= -4\pi\sigma_d^2 \int_0^{2r_{LME}} \exp\left(\frac{z^2}{-2\sigma_d^2}\right) \left(z^2 \left(r_{LME}^2 - \tfrac{1}{4}z^2\right)\right)^{1/4} dz + 2\pi^2\sigma_d^2 r_{LME}^2. \tag{S13.0.34}$$

Proof. We start by examining

$$\int_{(x_2,y_2)\in U} \varphi((x_1, y_1), (x_2, y_2)) dx_2 dy_2 = \int_{(x_2,y_2)\in U} \exp\left(\frac{(x_1 - x_2)^2 + (y_1 - y_2)^2}{-2\sigma_d^2}\right) dx_2 dy_2 \tag{S13.0.35}$$

where U is either $\mathbb{R}^2 \setminus D$ or D. Without loss of generality we can assume D is centered at the origin and (x_1, y_1) is on the non-positive x axis. Substituting $x = x_2 - x_1$ and $y = y_2 - y_1$, the integral becomes

$$\int_{U-(x_1,y_1)} \exp\left(\frac{x^2 + y^2}{-2\sigma_d^2}\right) dx dy. \tag{S13.0.36}$$

We let (ρ, θ) be the polar coordinates for (x, y), so that $x = \rho\cos(\theta)$, $y = \rho\sin(\theta)$, $dxdy = \rho d\rho d\theta$. See, for instance, [29] for a review of integration in polar coordinates. The integral becomes

$$\int_{U-(x_1,y_1)} \exp\left(\frac{\rho^2}{-2\sigma_d^2}\right) \rho d\rho d\theta. \tag{S13.0.37}$$

By the law of cosines (see figure S13),

$$r_{\text{LME}}^2 = \rho^2 + \rho_1^2 - 2\rho\rho_1\cos(\theta) \tag{S13.0.38}$$

where (ρ_1, θ_1) are the polar coordinates for (x_1, y_1) (so $\rho_1 = -x_1$ using the assumption that (x_1, y_1) is on the non-positive x axis). Solving S13.0.38 gives

$$\rho = \rho_1 \cos(\theta) \pm \sqrt{\rho_1^2 \cos^2(\theta) - \rho_1^2 + r_{\mathrm{LME}}^2}. \tag{S13.0.39}$$

But because $(x_1, y_1) \in D$ we know $\rho_1 < r$, so one of these solutions is negative and therefore invalid. So

$$\rho = \rho_1 \cos(\theta) + \sqrt{\rho_1^2 \cos^2(\theta) - \rho_1^2 + r_{\mathrm{LME}}^2}. \tag{S13.0.40}$$

For $U = \mathbb{R}^2 \setminus D$, S13.0.37 therefore becomes

$$\int_{-\pi}^{\pi} \int_{\rho_1 \cos(\theta)+\sqrt{\rho_1^2 \cos^2(\theta)-\rho_1^2+r_{\mathrm{LME}}^2}}^{\infty} \exp\left(\frac{\rho^2}{-2\sigma_d^2}\right) \rho d\rho d\theta \tag{S13.0.41}$$

and for $U = D$ it becomes

$$\int_{-\pi}^{\pi} \int_{0}^{\rho_1 \cos(\theta)+\sqrt{\rho_1^2 \cos^2(\theta)-\rho_1^2+r_{\mathrm{LME}}^2}} \exp\left(\frac{\rho^2}{-2\sigma_d^2}\right) \rho d\rho d\theta. \tag{S13.0.42}$$

The expression S13.0.41 simplifies to

$$\sigma_d^2 \int_{-\pi}^{\pi} \exp\left(\frac{(\rho_1 \cos(\theta) + \sqrt{\rho_1^2 \cos^2(\theta) - \rho_1^2 + r_{\mathrm{LME}}^2})^2}{-2\sigma_d^2}\right) d\theta \tag{S13.0.43}$$

and S13.0.42 simplifies to

$$-\sigma_d^2 \int_{-\pi}^{\pi} \exp\left(\frac{(\rho_1 \cos(\theta) + \sqrt{\rho_1^2 \cos^2(\theta) - \rho_1^2 + r_{\mathrm{LME}}^2})^2}{-2\sigma_d^2}\right) d\theta + 2\pi\sigma_d^2. \tag{S13.0.44}$$

To evaluate S13.0.31 and S13.0.33 we integrate S13.0.43 and S13.0.44, respectively, over $(x_1, y_1) \in D$. So S13.0.31 is

$$\sigma_d^2 \int_{-\pi}^{\pi} \int_0^{r_{\mathrm{LME}}} \int_{-\pi}^{\pi} \exp\left(\frac{(\rho_1 \cos(\theta)+\sqrt{\rho_1^2 \cos^2(\theta)-\rho_1^2+r_{\mathrm{LME}}^2})^2}{-2\sigma_d^2}\right) d\theta \rho_1 d\rho_1 d\theta_1 \tag{S13.0.45}$$

$$= 2\pi\sigma_d^2 \int_{-\pi}^{\pi} \int_0^{r_{\mathrm{LME}}} \exp\left(\frac{(\rho_1 \cos(\theta)+\sqrt{\rho_1^2 \cos^2(\theta)-\rho_1^2+r_{\mathrm{LME}}^2})^2}{-2\sigma_d^2}\right) \rho_1 d\rho_1 d\theta \tag{S13.0.46}$$

and S13.0.33 is

$$-\sigma_d^2 \int_{-\pi}^{\pi} \int_0^{r_{\mathrm{LME}}} \int_{-\pi}^{\pi} \exp\left(\frac{(\rho_1 \cos(\theta)+\sqrt{\rho_1^2 \cos^2(\theta)-\rho_1^2+r_{\mathrm{LME}}^2})^2}{-2\sigma_d^2}\right) d\theta \rho_1 d\rho_1 d\theta_1 + 2\pi^2\sigma_d^2 r_{\mathrm{LME}}^2 \tag{S13.0.47}$$

$$= -2\pi\sigma_d^2 \int_{-\pi}^{\pi} \int_0^{r_{\mathrm{LME}}} \exp\left(\frac{(\rho_1 \cos(\theta)+\sqrt{\rho_1^2 \cos^2(\theta)-\rho_1^2+r_{\mathrm{LME}}^2})^2}{-2\sigma_d^2}\right) \rho_1 d\rho_1 d\theta + 2\pi^2\sigma_d^2 r_{\mathrm{LME}}^2. \tag{S13.0.48}$$

Substituting $x_3 = \rho_1 \cos(\theta)$, $y_3 = \rho_1 \sin(\theta)$, $dx_3 dy_3 = \rho_1 d\rho_1 d\theta$, and $\rho_1^2 = x_3^2 + y_3^2$ makes S13.0.46 equal to

$$2\pi\sigma_d^2 \int_{(x_3,y_3)\in D} \exp\left(\frac{(x_3 + \sqrt{y_3^2 + r_{\mathrm{LME}}^2})^2}{-2\sigma_d^2}\right) dx_3 dy_3 \tag{S13.0.49}$$

and S13.0.48 equal to

$$-2\pi\sigma_d^2 \int_{(x_3,y_3)\in D} \exp\left(\frac{(x_3 + \sqrt{y_3^2 + r_{\mathrm{LME}}^2})^2}{-2\sigma_d^2}\right) dx_3 dy_3 + 2\pi^2\sigma_d^2 r_{\mathrm{LME}}^2. \tag{S13.0.50}$$

To simplify the integral that appears in S13.0.49 and S13.0.50, we use the transformation

$$x_4 = x_3 + \sqrt{y_3^2 + r_{\mathrm{LME}}^2} \tag{S13.0.51}$$

$$y_4 = y_3. \tag{S13.0.52}$$

Because

$$\det \begin{pmatrix} \frac{\partial x_4}{\partial x_3} & \frac{\partial x_4}{\partial y_3} \\ \frac{\partial y_4}{\partial x_3} & \frac{\partial y_4}{\partial y_3} \end{pmatrix} = \det \begin{pmatrix} 1 & \frac{\partial x_4}{\partial y_3} \\ 0 & 1 \end{pmatrix} = 1, \tag{S13.0.53}$$

this is an area-preserving transformation (see, for instance, theorem 3.13 on p. 67 of [53]). So

$$\int_{(x_3,y_3)\in D} \exp\left(\frac{(x_3 + \sqrt{y_3^2 + r_{\mathrm{LME}}^2})^2}{-2\sigma_d^2}\right) dx_3 dy_3 = \int_{\Omega} \exp\left(\frac{x_4^2}{-2\sigma_d^2}\right) dx_4 dy_4, \tag{S13.0.54}$$

where the latter integral is over Ω, the image of D under the transformation. It is straightforward to show that Ω is bounded on the left and the right by 0 and $2r_{\mathrm{LME}}$, respectively. To find the upper and lower bounds we substitute from S13.0.51 and S13.0.52 into the equation for the boundary of D, $x_3^2 + y_3^2 = r_{\mathrm{LME}}^2$:

$$(x_4 - \sqrt{y_4^2 + r_{\mathrm{LME}}^2})^2 + y_4^2 = r_{\mathrm{LME}}^2. \tag{S13.0.55}$$

After some algebraic manipulation, this comes to

$$y_4^4 = x_4^2\left(r_{\text{LME}}^2 - \frac{1}{4}x_4^2\right). \tag{S13.0.56}$$

So the right side of S13.0.54 becomes

$$\int_0^{2r_{\text{LME}}}\int_{\left(x_4^2\left(r_{\text{LME}}^2-\frac{1}{4}x_4^2\right)\right)^{1/4}}^{-\left(x_4^2\left(r_{\text{LME}}^2-\frac{1}{4}x_4^2\right)\right)^{1/4}} \exp\left(\frac{x_4^2}{-2\sigma_d^2}\right) dy_4 dx_4 \tag{S13.0.57}$$

$$= 2\int_0^{2r_{\text{LME}}} \exp\left(\frac{x_4^2}{-2\sigma_d^2}\right)\left(x_4^2\left(r_{\text{LME}}^2 - \frac{1}{4}x_4^2\right)\right)^{1/4} dx_4. \tag{S13.0.58}$$

Substituting this into S13.0.49 and S13.0.50 proves the lemma. □

References

[1] Abramowitz, M., Stegun, I.A. (1965) *Handboook of Mathematical Functions.* Dover, New York.

[2] Andersen, K., Beyer, J.E. (2006) Asymptotic size determines species abundance in the marine size spectrum. *The American Naturalist* 168, 54-61.

[3] Barnes, C., Maxwell, D., Reuman, D.C., Jennings, S. (2010) Global patterns in predator-prey size relationships reveal size dependency of trophic transfer efficiency. *Ecology* 91, 222-232.

[4] Blanchard, J.L., Jennings, S., Law, R., Castle, M.D., McCloghrie, P., Rochet, M.J., Benoit, E. (2009) How does abundance scale with body size in coupled, size-structured food webs? *Journal of Animal Ecology* 78 270-280.

[5] Bokma, F. (2004) Evidence against universal metabolic allometry. *Functional Ecology* 18, 184-187.

[6] Brett, J.R. (1964) The respiratory metabolism and swimming performance of young sockeye salmon. *Journal of the Fisheries Research Board of Canada*, 21, 1183-1226.

[7] Brown, J.H., Gillooly, J.F., Allen, A.P., Savage, V.M., West, G.B. (2004) Toward a metabolic theory of ecology. *Ecology* 85, 1771-1789.

[8] Burnham, K.P., Anderson, D.R. (2002) *Model Selection and Multimodel Inference: A Practical Information-Theoretic Approach, Second Edition.* Springer Science and Business Media, New York.

[9] Cheung, W.W.L., Sarmiento, J.L., Dunne, J., Frölicher, T.L., Lam, V.W.Y., Deng Palomares, M.L., Watson, R., Pauly, D. (2013) Shrinking of fishes exacerbates impacts of global ocean changes on marine ecosystems. *Nature Climate Change* 3, 254-258.

[10] Ciancio, J., Pascual, M.A., Beauchamp, D.A. (2007) Energy density of Patagonian aquatic organisms and empirical predictions based on water content. *Transactions of the American Fisheries Society* 136, 1415-1422.

[11] Clarke, A., Johnston, N.M. (1999) Scaling of metabolic rate with body mass and temperature in teleost fish. *Journal of Animal Ecology* 68, 893-905.

[12] Cohen, J. E., Jonsson, T., Carpenter, S.R. (2003) Ecological community description using the food web, species abundance and body size. *Proceedings of National Academy of Sciences* 100, 1781-1786.

[13] Cohen, J. E., Schittler, D. N., Raffaelli, D. G., Reuman, D. C. (2009) Food webs are more than the sum of their tri-trophic parts. *Proceedings of the National Academy of Sciences* 106, 22335-22340.

[14] Compagno, L.J.V. (1998) Sphyrnidae. Hammerhead and bonnethead sharks. p. 1361-1366. In: K.E. Carpenter and V.H. Niem (eds.) FAO identification guide for fishery purposes. The Living Marine Resources of the Western Central Pacific. FAO, Rome.

[15] Daly, M., Brugler, M.R., Cartwright, P., Collins, A.G., Dawson, M.N., Fautin, D.G., France, S.C., McFadden, C.S., Opresko, D.M., Rodriguez, E., Romano, S.L., Stake, J.L. (2007) The phylum Cnidaria: A review of phylogenetic patterns and diversity 300 years after Linnaeus. *Zootaxa* 1668, 127-182.

[16] Dial, K.P., Marzluff, J.M. (1988) Are the smallest organisms the most diverse? *Ecology* 69, 1620-1624.

[17] Drazen, J.C., Reisenbichler, K.R., Robinson, B.H. (2007) A comparison of absorption and assimilation efficiencies between four species of shallow- and deep-living fishes. *Marine Biology* 151, 1551-1558.

[18] Duarte, C.M., Alcaraz, M. (1989) To produce many small or few large eggs: a size-independent reproductive tactic of fish. *Oecologia* 80, 401-404.

[19] Etienne, R.S., Alonso, D. (2007) Neutral community theory: How stochasticity and dispersal limitation can explain species coexistence. *Journal of Statistical Physics* 128, 485-510.

[20] Etienne, R.S., Olff, H. (2004) How dispersal limitation shapes species body size distributions in local communities. *The American Naturalist* 163, 69-83.

[21] Froese, R., Pauly, D. (Eds) (2011) FishBase. World Wide Web electronic publication. `http://www.fishbase.org`.

[22] Gillooly, J.F., Brown, J.H., West, G.B., Savage, V.M., Charnov, E.L. (2001) Effect of size and temperature on metabolic rate. *Science* 293, 2248-2251.

[23] Hendriks, J.A., Mulder, C. (2008) Scaling of offspring number and mass to plant and animal size: model and metanalysis. *Oecologia* 155, 705-716.

[24] Hester, F.J. (1968) Visual contrast thresholds of the goldfish (*Carassins auratus*). *Vision Research* 8, 1315-1336.

[25] Hislop, J.R.G. (1984) A comparison of the reproductive tactics and strategies of cod, haddock, whiting and Norway pout in the Northern Sea. In: Potts GW, Wootton RJ (eds) *Fish reproduction: strategies and tactics.* Academic Press, Oxford, pp 311-330.

[26] Hubbell, S.P. (2001) *The Unified Neutral Theory of Biodiversity and Biogeography.* Princeton University Press, Princeton.

[27] Jennings, S., Pinnegar, J.K., Polunin, N.V.C., Boon, T. (2001) Weak cross-species relationships between body size and trophic level belie powerful size-based trophic structuring in fish communities. *Journal of Animal Ecology* 70, 934-944.

[28] Kamler, E. (2005) Parent-egg-progeny relationships in teleost fishes: an energetic perspective. *Reviews in Fish Biology and Fisheries* 15, 399-421.

[29] Larson, R.E., Hostetler, R.P., Edwards, B.H. (1990) *Calculus with Analytic Geometry, Fourth Edition.* D.C. Heath and Company, Lexington, Massachusetts.

[30] Miller, P.J. (1984) The tokology of gobioid fishes. In: Potts GW, Wootton RJ (eds) *Fish reproduction: strategies and tactics.* Academic Press, Oxford, pp 119-154

[31] Nee, S. (2005) The neutral theory of biodiversity: do the numbers add up? *Functional Ecology* 19, 173176.

[32] Nelson, J.S. (1994) *Fishes of the World.* Wiley, New York.

[33] Nesis, K.N. (1982) *Abridged key to the cephalopod mollusks of the world's ocean.* Light and Food Industry Publishing House, Moscow. (In Russian.). Translated into English by B.S. Levitov, ed. by L. A. Burgess (1987), *Cephalopods of the world.* T.F.H. Publications, Neptune City, NJ.

[34] Orme, C.D.L., Isaac, N.J.B., Purvis, A. (2002) Are most species small bodied? Not within species-level phylogenies. *Proceedings of the Royal Society of London Series B-Biological Sciences* 269, 1279-1287.

[35] Peters, R.H. (1983) *The Ecological Implications of Body Size.* Cambridge University Press, Cambridge.

[36] Petrakis, G., Stergiou, K.I. (1995) Weight-length relationships for 33 fish species in Greek waters. *Fisheries Research* 21, 465-469.

[37] Purvis, A., Orme, C.D.L., Dolphin, K. (2003) Why are most species small-bodied? p. 155-173. In: *Macroecology: Concepts and Consequences.* Oxford University Press, Oxford.

[38] Quéro, J.-C. (1984) Sphyrnidae. p. 122-125. In P.J.P. Whitehead, M.-L. Bauchot, J.-C. Hureau, J. Nielsen, and E. Tortonese (eds.) Fishes of the north-eastern Atlantic and the Mediterranean. UNESCO, Paris. Vol. 1.

[39] Ricklefs, R.E. (2003) A comment on Hubbells zero-sum ecological drift model. *Oikos* 100, 185192.

[40] Ricklefs, R.E. (2006) The unified neutral theory of biodiversity: do the numbers add up? *Ecology* 87, 14241431.

[41] Rossberg, A. (2012) A complete analytic theory for structure and dynamics of populations and communities spanning wide ranges in body size. *Advances in Ecological Research* 46, 427-521.

[42] Rossberg, A. (2013) *Food Webs and Biodiversity: Foundations, Models, Data.* Wiley, Oxford.

[43] Sturm, E.A., Horn, M.H. (1998) Food habits, gut morphology and pH, and assimilation efficiency of the zebrapersh *Hermosilla azurea*, an herbivorous kyphosid fish of temperate marine waters. *Marine Biology* 132, 515-522.

[44] Pinheiro, J.C., Bates, D.M. (2000) *Mixed-Effects Models in S and S-Plus.* Springer-Verlag, New York.

[45] Pope, J. G., Rice, J. C., Daan, N., Jennings, S., Gislason, H. (2006) Modelling an exploited marine fish community with 15 parameters: results from a simple size-based model. *ICES Journal of Marine Science* 63, 1029-1044.

[46] Poulin, R. (1995) Clutch size and egg size in free-living and parasitic copepods: a comparative analysis. *Evolution* 49, 325-336.

[47] Reuman, D.C., Mulder, C., Raffaelli, D., Cohen, J.E. (2008) Three allometric relations of population density to body mass: Theoretical integration and empirical tests in 149 food webs. *Ecology Letters* 11, 1216-1228.

[48] Robertson, D.R. (2008) Global biogeographical data bases on marine fishes: caveat emptor. *Diversity and Distributions* 14, 891-892.

[49] Roper, C.F.E., Sweeney, M.J., Nauen, C. (1984) *Cephalopods of the World.* FAO Fisheries Synposis 125: 1-327.

[50] Rosa, R., Dierssen, H.M., Gonzalez, L., Seibel, B.A. (2008) Large-scale diversity patterns of cephalopods in the Atlantic Ocean and deep sea. *Ecology* 89, 3449-3461.

[51] Rosindell, J., Cornell, S.J., Hubbbell, S.P., Etienne, R.S. (2010) Protracted speciation revitalizes the neutral theory of biodiversity. *Ecology Letters* 13, 716-727.

[52] Santos, M.N., Gaspar, M.B., Vasconcelos, P., Monteiro, C.C. (2002) Weight-length relationships for 50 selected fish species of the Algarve coast (southern Portugal). *Fisheries Research* 59, 289-295.

[53] Spivak, M. (1965) *Calculus on Manifolds.* Adison-Wesley Publishing Company, New York.

[54] Thygesen, U.H., Farnsworth, K.D., Andersen, K.H., Beyer, J.E. (2005) How optimal life history changes with the community size spectrum. *Proceedings of the Royal Society B* 272, 1323-1331.

[55] Trebilco, R., Baum, J.K., Salomon, A.K., Dulvy, N.K. (2013) Ecosystem ecology: size-based constraints on the pyramids of life. *Trends in Ecology and Evolution* 28, 423-431.

[56] Vallade, M., Houchmmandzadeh, B. (2003) Analytic solution of a neutral model of biodiversity. *Physical Review E* 68, 061902.

[57] Van Valen, L. (1973) Body size and numbers of plants and animals. *Evolution* 27, 27-35.

[58] Ware, D.M. (1978) Bioenergetics of pelagic fish: Theoretical change in swimming speed in relation with body size. *Journal of the Fisheries Research Board of Canada* 35, 220-228.

[59] West, G.B., Brown, J.H., Enquist, B.J. (2001) A general model for ontogenetic growth. *Nature* 413, 628-631.

[60] White, C.R, Phillips, N.F., Seymour, R.S. (2006) The scaling and temperature dependence of vertebrate metabolism. *Biology Letters* 2, 125-127.

[61] White, E.P., Ernest, S.K.M., Kerkhoff, A.J., Enquist, B.J. (2007) Relationships between size and abundance in ecology. *Trends in Ecology and Evolution* 22, 323-330.

[62] White, E.P., Enquist, B.J., Green, J.L. (2008) On estimating the exponent of power-law frequency distributons. *Ecology* 89, 905-912.

Figure S1. Plot of logit($\mathfrak{m}$) against $\log_e(r_R/\sigma_d)$. Here logit($\mathfrak{m}$) is defined as $\log_e(\mathfrak{m}/(1-\mathfrak{m}))$. See section S5.1.

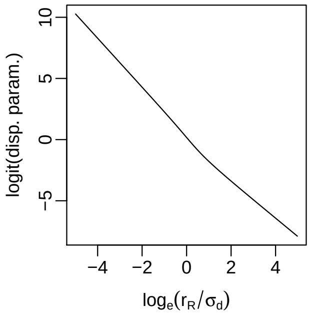

Figure S2. Egg mass limit, $f(m_\infty)$. Average egg masses versus average adult masses of copepod families, from [46] (open circles), and the approximate upper bound of marine teleost egg masses versus estimated maximal mass, from [18] (solid lines). See section S8.4.

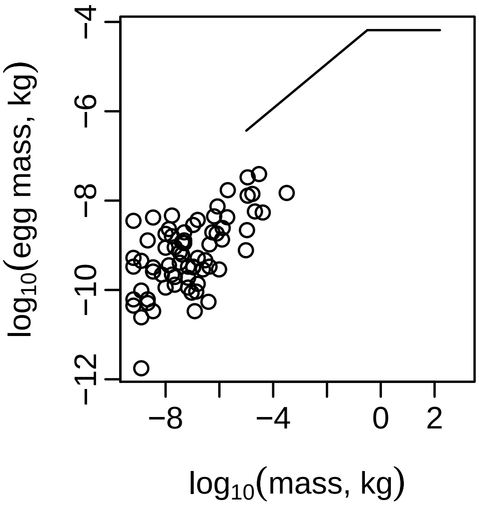

Figure S3. Accuracy of approximations used for J_C, J_M, and S_M. The solid black lines (which overlap with the solid red lines for much of their length) are from S9.1.2 for $\alpha = 1.05$ (A), $\alpha = 1.25$ (B), $\alpha = 1.5$ (C), and $\alpha = 2$ (D). The dashed black lines are the best linear approximations to the solid black lines, and have slope -0.49 to two significant figures on all panels; the good fit and slope of these lines show that S9.1.4 is a good approximation. The solid red lines have derivative equal to 97.8% of the derivative of the solid black lines, and represent upper bounds for the scaling of S_M (see S9.2.12 and S9.2.13). The dashed red lines are the best linear approximations to the solid red lines, and have slope -0.48 to two significant figures on all panels. The notation J is used to represent a variable proportional to both J_C and J_M.

A, B, C, D

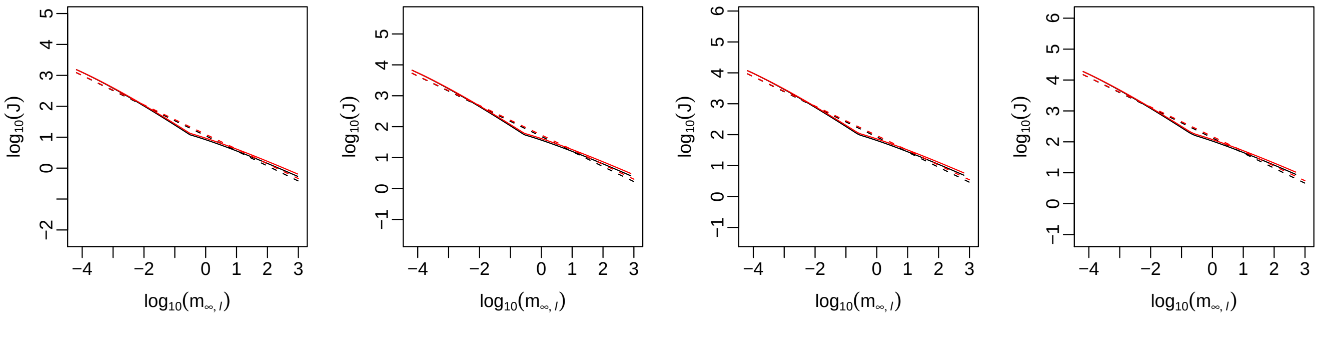

FIGURE S4. Accuracy of approximations used for S_M. The solid red lines use S9.2.1; dashed red lines are the best linear approximations to the solid red lines and have slope -0.49 to two significant figures on all panels. Solid black lines, which overlap with the red lines for most of their length, use the exact equation S9.2.15, showing good agreement with the approximation. There are 414 solid black lines on each panel, produced using a grid of 414 values of the parameter pair consisting of $\nu/(1-\nu)$ and J_M in the reference category $m_{\infty,l} = (1000/\alpha)$kg to 1000kg. Points in the grid of pairs spanned the constraints $10^6 \leq J_M \leq 10^{26}$ and $\frac{1}{10 J_M} \leq \frac{\nu}{1-\nu} \leq \frac{1}{10}$ and were evenly spaced on log-log axes. Black lines overlap and vary little overall. The values of α used were 1.05 (A), 1.25 (B), 1.5 (C), and 2 (D).

A, B, C, D

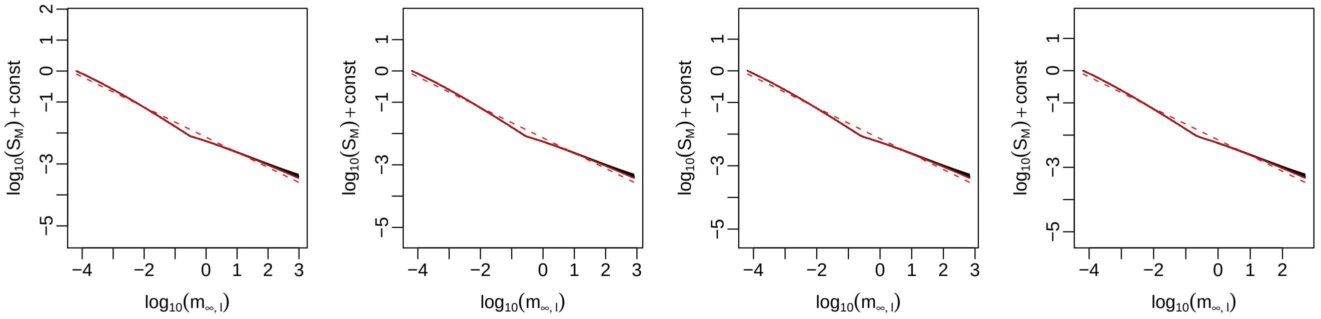

FIGURE S5. Accuracy of approximations used for S_C: example diversity spectra. Solid black lines are plots of S9.3.3 against $\log_{10}(m_{\infty,l})$ for: $\alpha = 1.05$ (A, B, C) and 2 (D, E, F); e_{σ_d} equal to 0.2 (A, D), 0.3 (B, E), and 0.4 (C, F); and for θ in the reference category $m_{\infty,l} = (1000/\alpha)$kg to 1000kg equal to 2, 4, 10, 20, 50, 100, 300, 500, and 11 more values whose base-10 logarithms are evenly spaced between 3 and 25 (multiple black lines appear on each panel and overlap almost entirely). Red lines reproduce the lines on main text figure 3A-C, and hence illustrate, though comparison with the black lines, the quality of the approximations used in the main text. Dashed black lines are best linear approximations of the mean of the solid black lines. Slopes of black and red dashed lines are given in the upper right of each panel, and were the same to two significant figures on each panel.

A, B, C

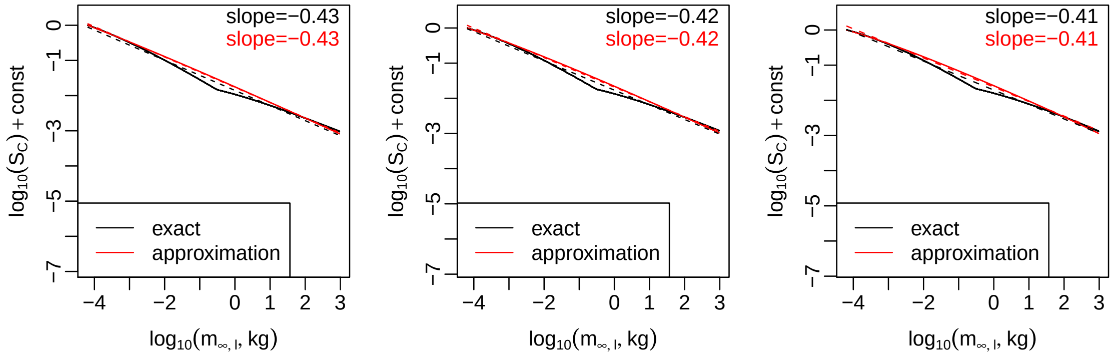

D, E, F

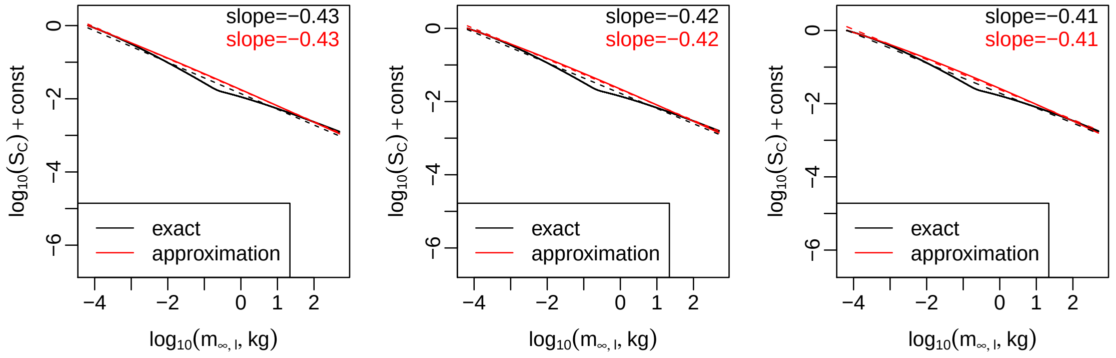

FIGURE S6. Accuracy of approximations used for S_C: diversity spectrum slopes. Plots are analogous to main text figure 3D-F, but use S9.3.3 instead of the approximations S9.3.1 and S9.3.2. The left three columns use $\alpha = 1.05$; the right three use $\alpha = 2$. Columns 1 and 4 use $e_{\sigma_d} = 0.2$, columns 2 and 5 use $e_{\sigma_d} = 0.3$, and columns 3 and 6 use $e_{\sigma_d} = 0.4$. Rows use values of θ in the reference category $m_{\infty,l} = (1000/\alpha)$kg to 1000kg equal to 2, 10, 300, 10^3, 10^{14}, and 10^{25}, respectively.

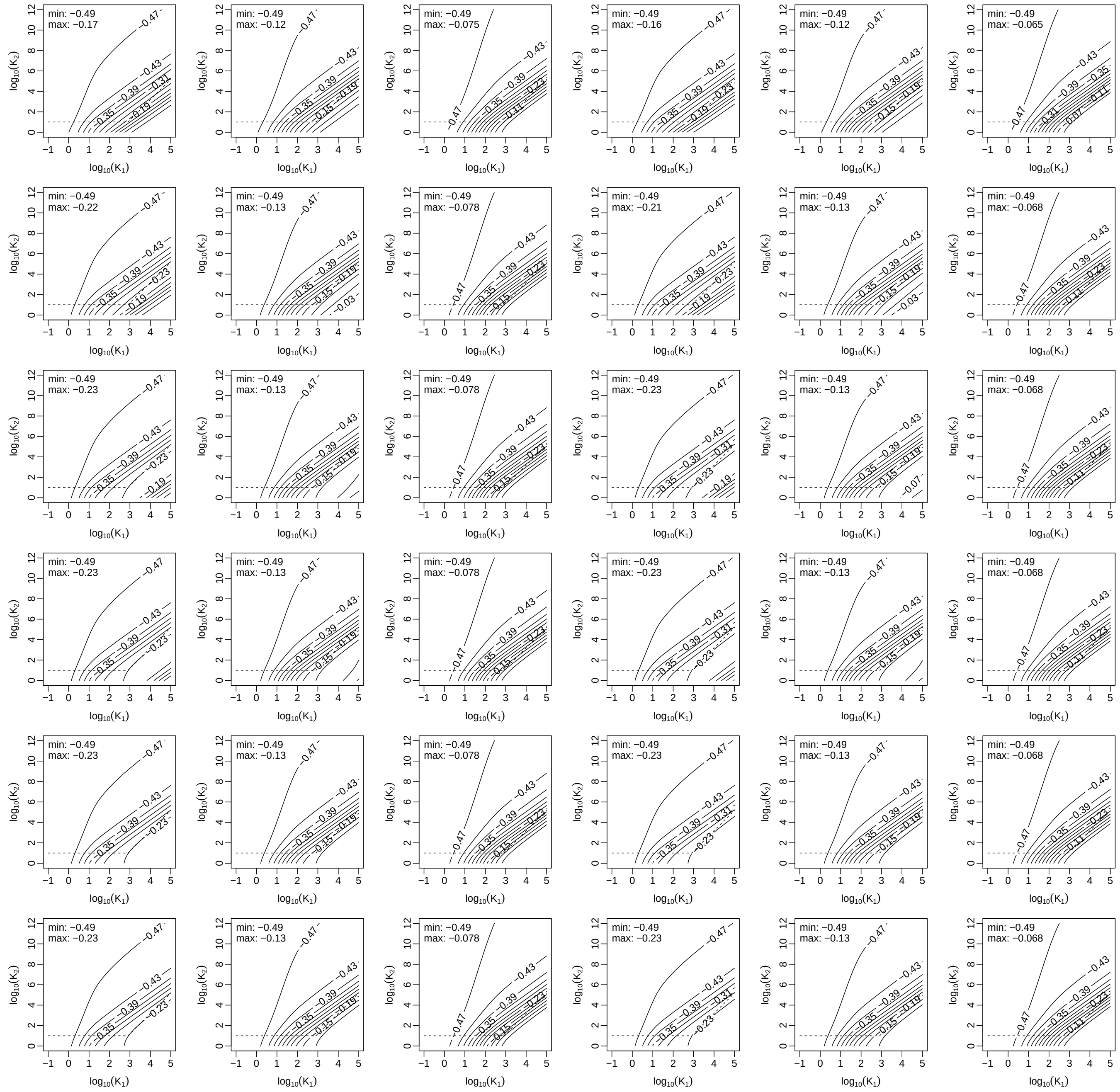

Figure S7. Map of LMEs. Codes correspond to table S3.

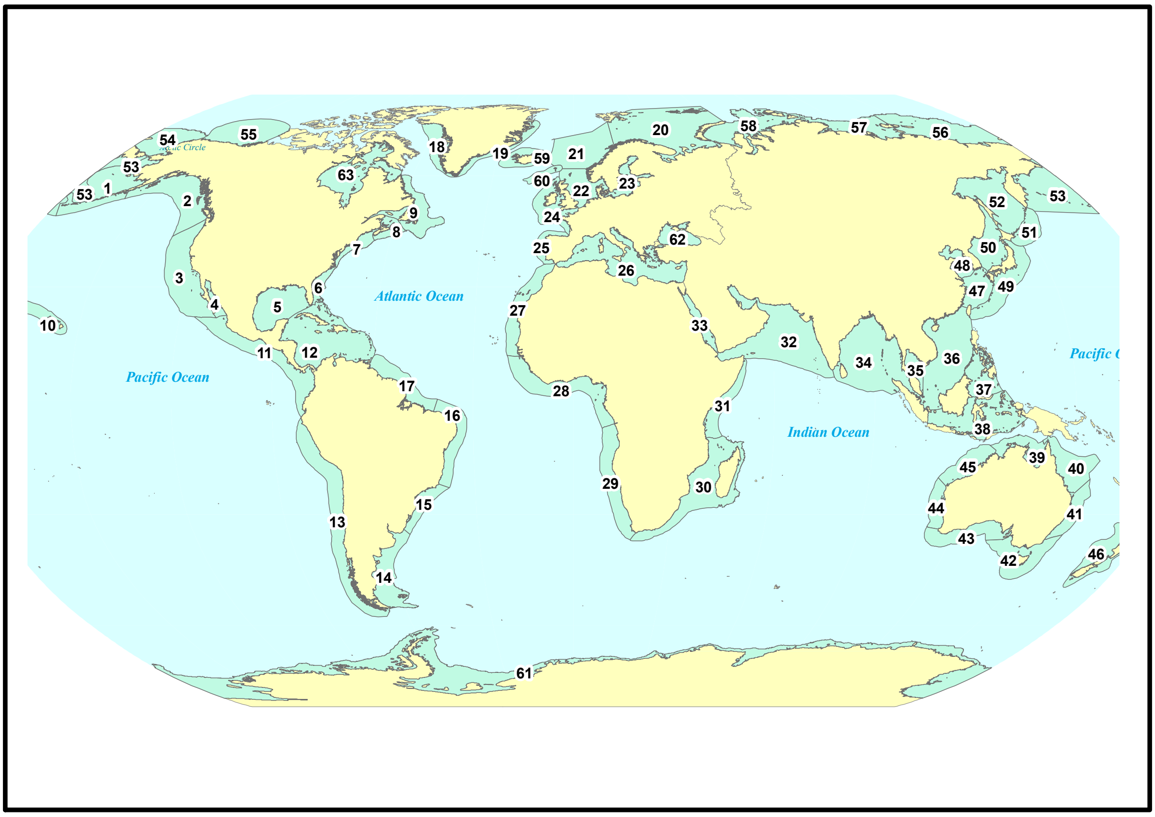

Figure S8. Map of provinces.

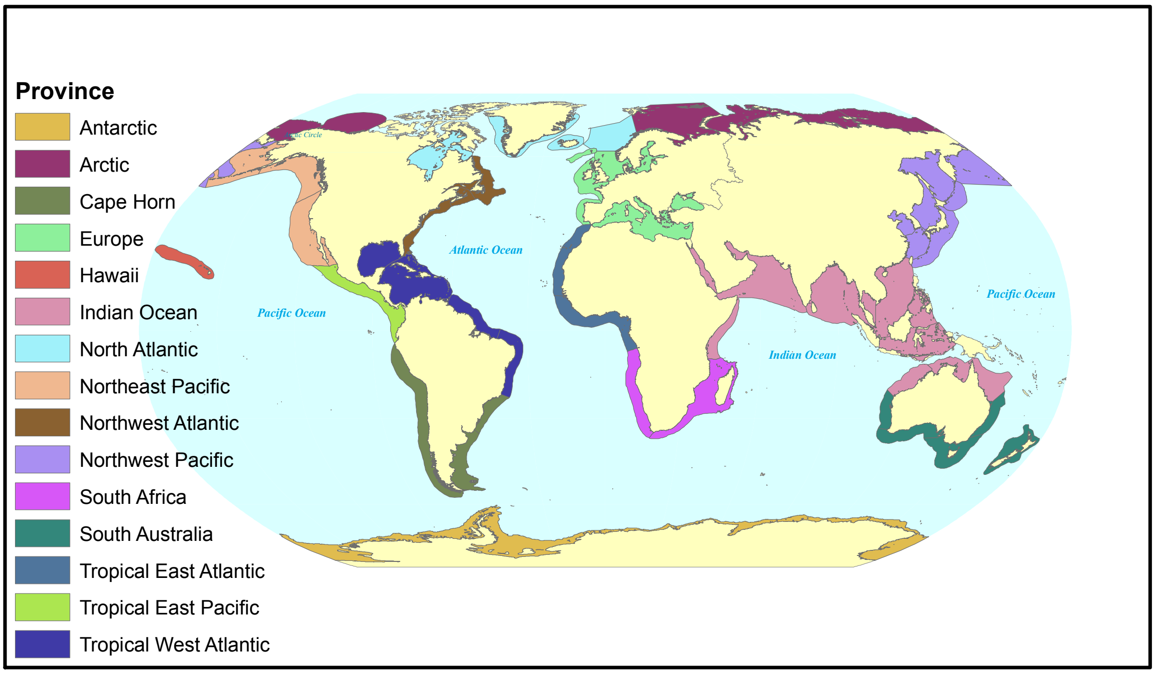

Figure S9. Map of basins.

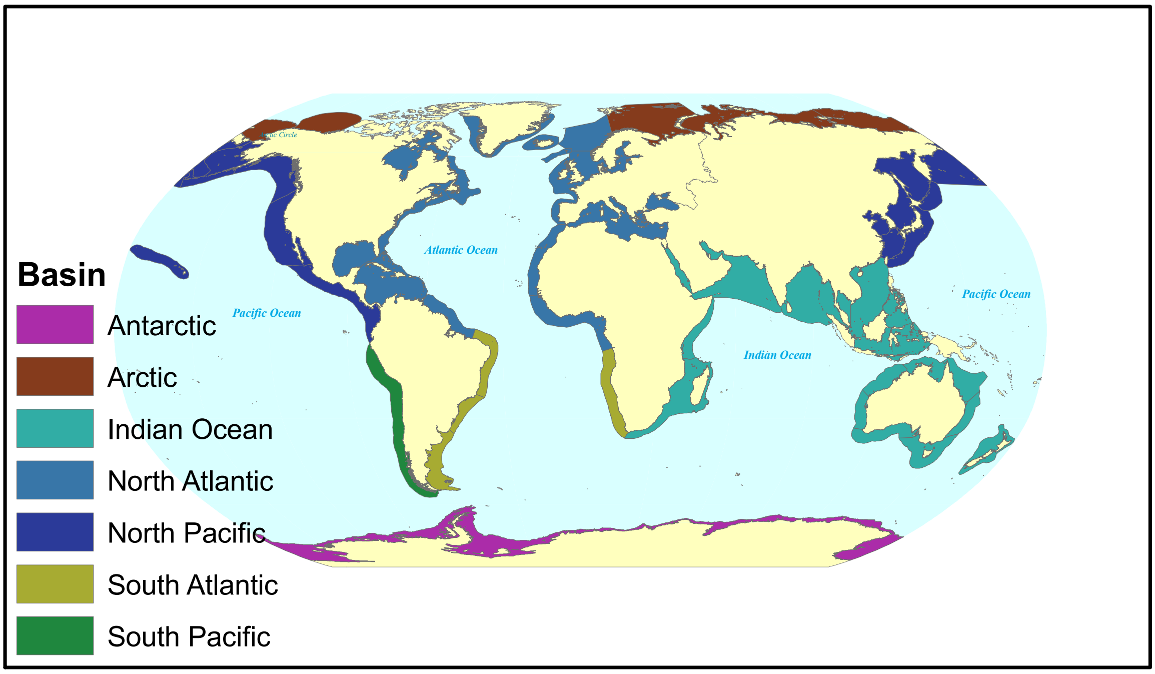

Figure S10. Map of latitudinal bands.

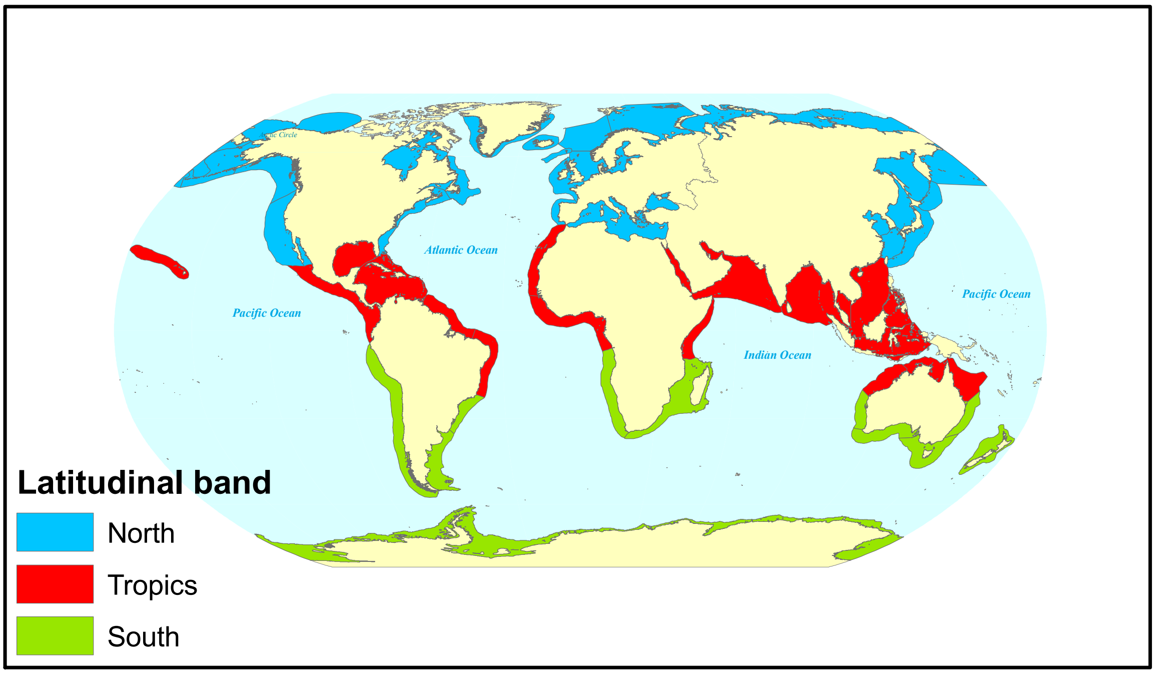

FIGURE S11. Diversity spectrum plots for regions for which the tP was rejected. Regions are indicated using numbers in the corners of each panel, according to the codes in tables S3 and S4. Panels in the first column show empirical diversity spectrum plots together with the diversity spectrum corresponding to the best-fitting tP distribution. When the two plots on these panels are similar, the diversity spectrum was close to linear. The second and third columns of panels show tP and qtP probability plots. When these plots are straight, the corresponding distribution was a reasonable fit for that region. The final column shows the diversity spectra corresponding to the best-fitting tP and qtP distributions. Results show that, except for a few LMEs (Baltic Sea, Faroe Plateau, Iceland Shelf, Norwegian Sea, West Greenland Shelf, which have codes 23, 60, 59, 21, and 18, respectively) and a single province (the North Atlantic, which has code 7) and a single basin (the South Atlantic, code 6), a linear diversity spectrum is a good approximation even for systems for which the tP was statistically rejected. It was a reasonable approximation even for some of the systems listed above.

LMEs

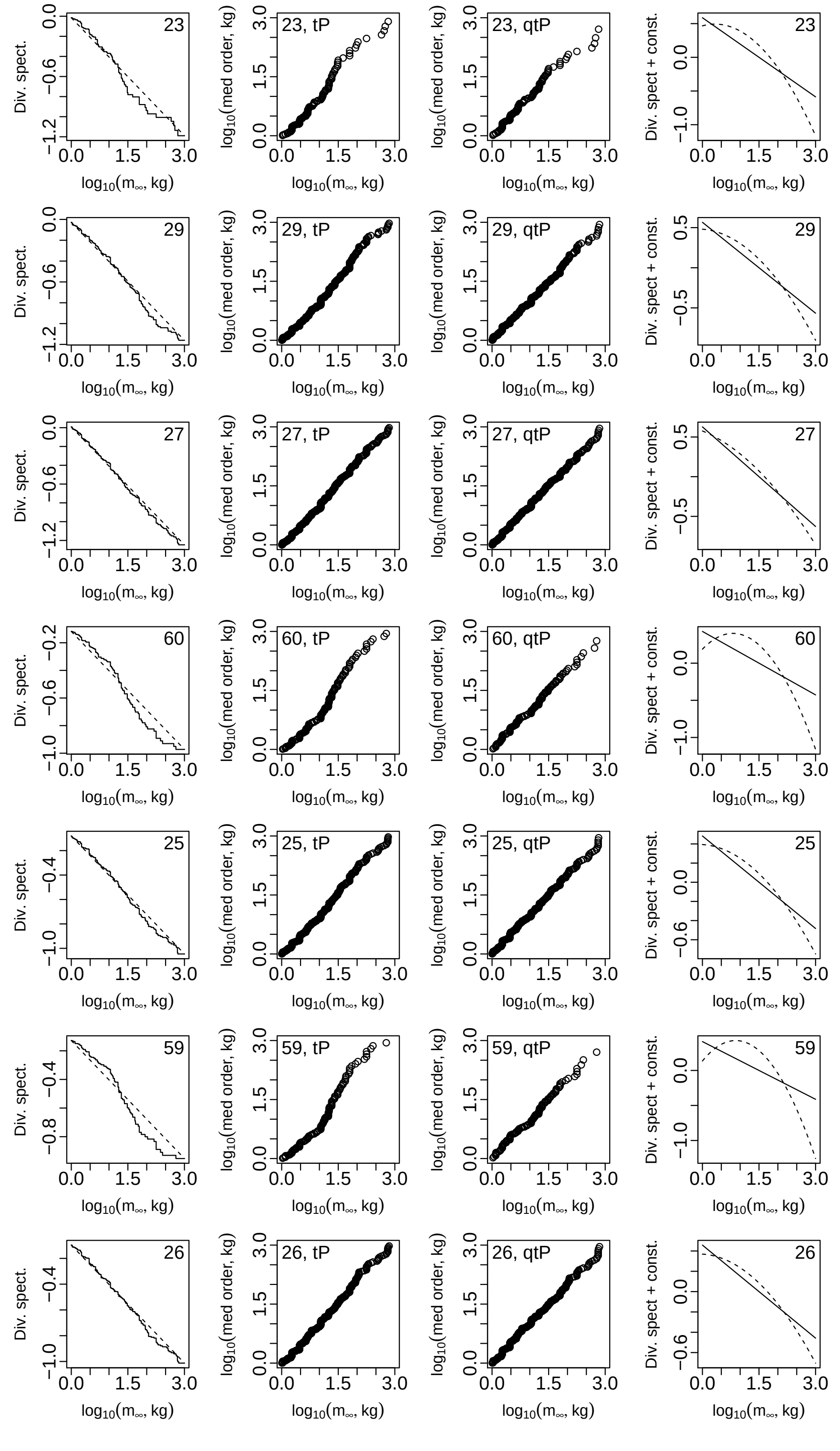

FIGURE S11

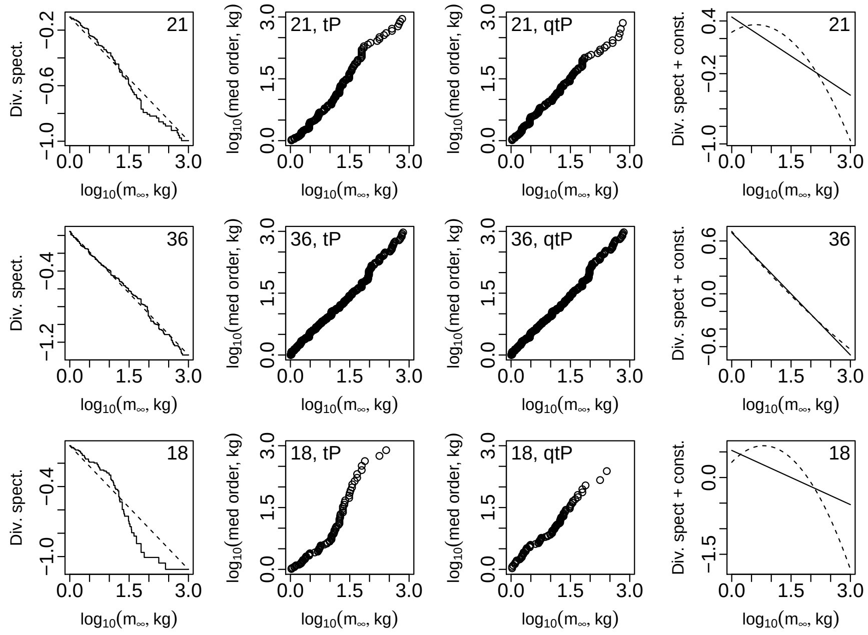

Provinces

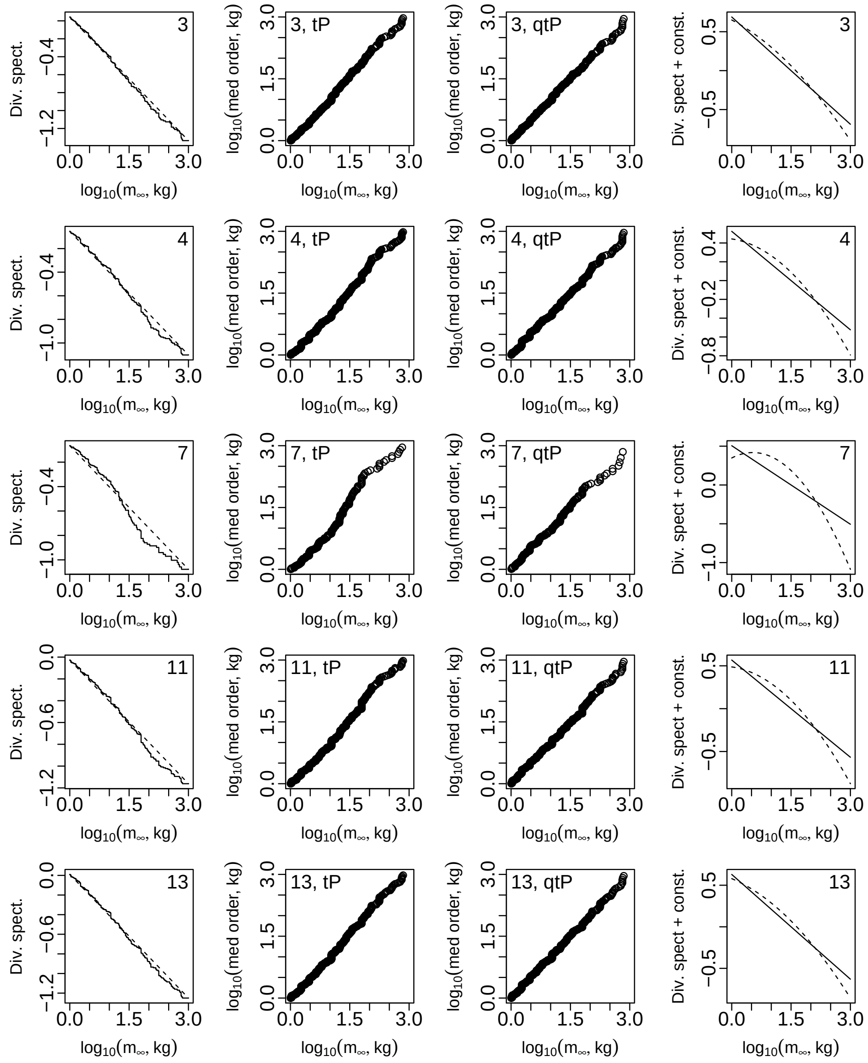

Basins

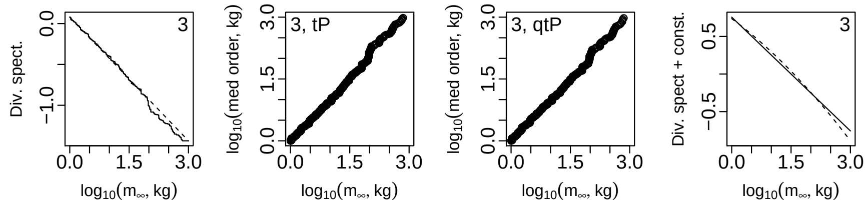

Figure S11

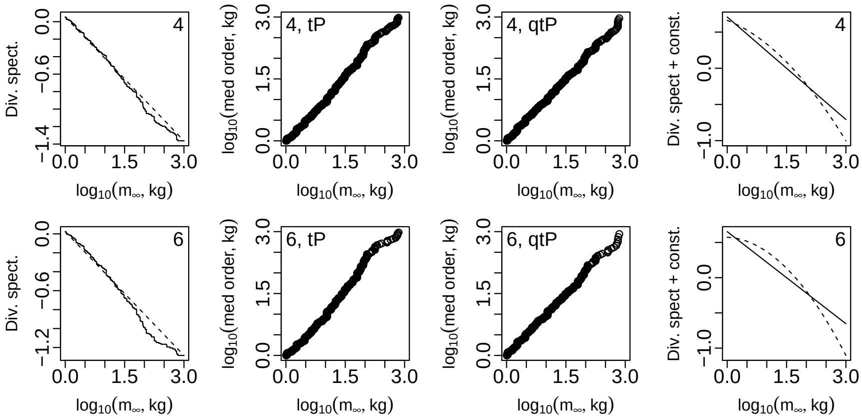

Latitudinal bands

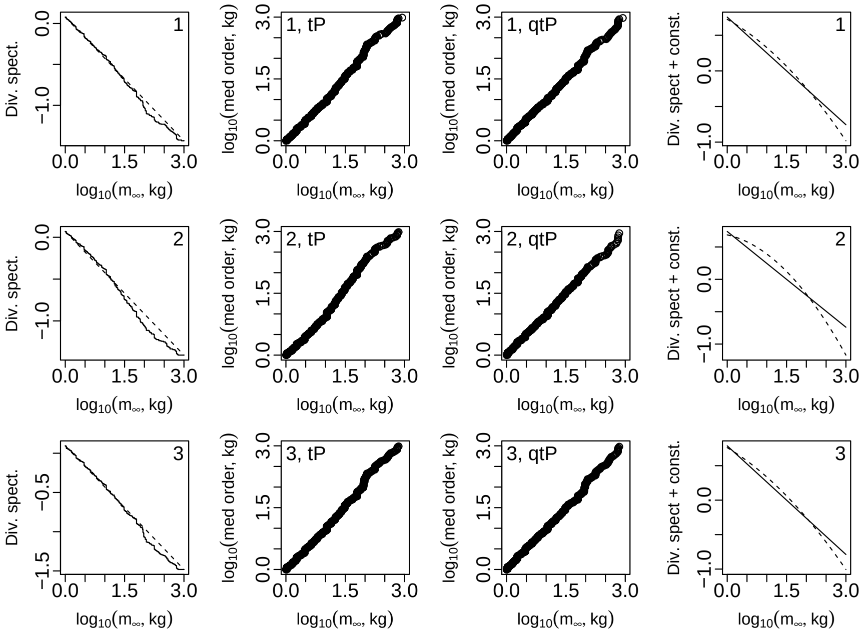

Global continental shelf seas

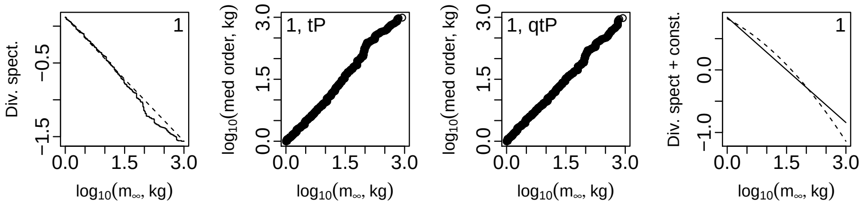

Figure S12. Diversity spectrum plots for the global region, including marine mammals. The first panel shows the empirical diversity spectrum together with the diversity spectrum corresponding to the best-fitting tP distribution. Because the two plots on these panels are similar, the diversity spectrum was close to linear. The second and third panels show tP and qtP probability plots. Because these plots are straight, the corresponding distributions were reasonable fits, and the qtP was hardly better than the tP. The final panel shows the diversity spectra corresponding to the best-fitting tP and qtP distributions; these are very similar. Results show that a linear diversity spectrum is a good but not perfect approximation, even though the tP distribution was statistically rejected (table S6) due to large sample size.

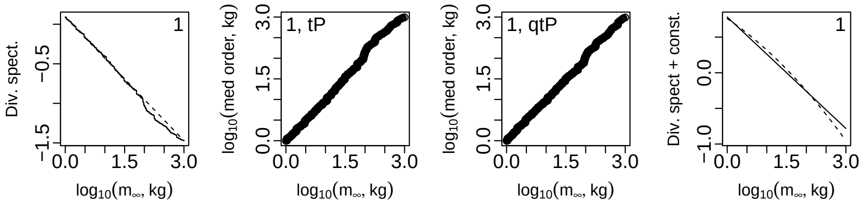

Figure S13. Diagram supporting S13.0.38.

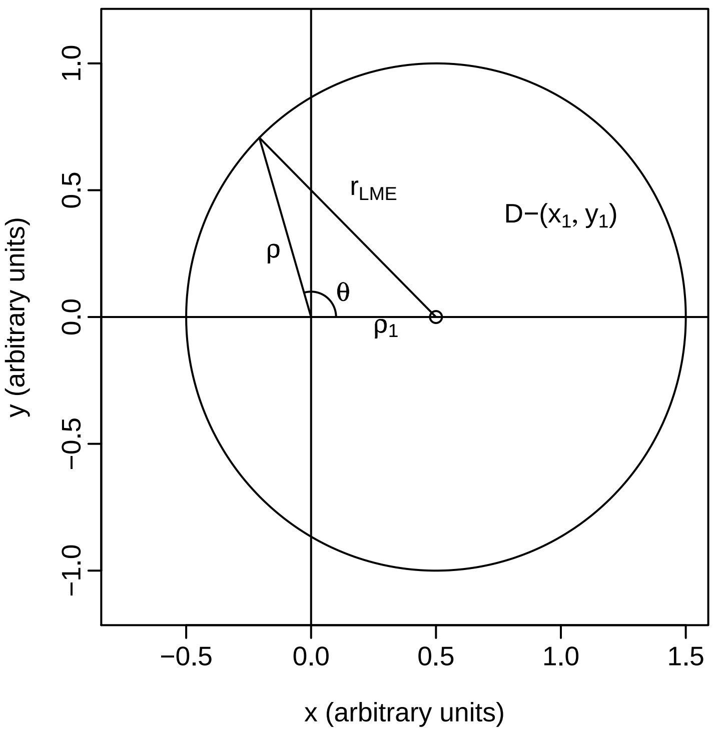

TABLE S1. Model parameters. ISD stands for individual size distribution, IASD stands for individual asymptotic-size distribution.

	Definition	Units	Value	Equation	Source
Cellular level					
m_{cell}	Mass of a cell	kg	3×10^{-12}	S4.5.1	[59]
Individual level					
E_{I_B}	Activation energy, basal metabolic rate	eV	0.5782	S3.0.4	S8.1
e_{I_B}	Mass exponent, basal metabolic rate	-	0.7982	S3.0.4	S8.1
E_{I_F}	Activation energy, field metabolic rate	eV	0.5782	S3.0.21	S3.0.23
e_{I_F}	Mass exponent, field metabolic rate	-	0.7982	S3.0.21	S3.0.24
$E_{u_{opt}}$	Activation energy, optimal swimming speed	eV	0.2816	S3.0.16	S3.0.18
$e_{u_{opt}}$	Mass exponent, optimal swimming speed	-	0.1342	S3.0.16	S3.0.19
e_v	Mass exponent, volume searched	-	0.8009	S4.1.3	S4.1.4
e_c	Mass exponent, consumption	-	0.7982	S4.2.13	S4.3.7
e_d	Mass exponent, death risk by predation	-	-0.2018	S4.4.11	S4.4.13
e_g	Mass exponent, ontogenetic growth	-	0.7982	S4.5.17	S4.5.16
k_{dg}	k_d/k_g	$\mathrm{kg}^{e_g-e_d-1}$	0.737	S4.6.5	S4.6.5
k_{ml}	Prefactor, mass-length allometry	$\mathrm{kg} \cdot \mathrm{m}^{-3}$	10	S3.0.9	[36, 52]
σ_f	Feeding kernel width parameter	-	1.0722	S4.2.5	S8.3.13
β_f	Preferred consumer-to-resource mass ratio	-	100	S4.2.5	[2, 54]
σ_r	Realized feeding kernel width parameter	-	1.0722	S8.3	S8.3.11
β_r	Realized consumer-to-resource mass ratio	-	1000	S8.3	[3]
w	See S3.0.6	-	0.58	S3.0.6	[58]
Efficiencies					
f_e	Energy obtained from 1kg ingested food	$\mathrm{J} \cdot \mathrm{kg}^{-1}$	3.793×10^6	S4.3	S4.3
f_{di}	Mass-energy conversion *after* crossing gut wall	$\mathrm{J} \cdot \mathrm{kg}^{-1}$	4.462×10^6	S4.3	[10]
f_{as}	Fraction ingested food crossing gut wall	-	0.85	S4.3	[17, 43]
f_g	Energy required to produce a cell	J	2.1×10^{-5}	S4.5.1	[59]
Physical properties of the ecosystem					
E_μ	Activation energy, dynamic viscosity of water	eV	-0.1781	S3.0.15	S8.2
Egg sizes					
m_{egg}	See S4.6.2	kg	6.5×10^{-5}	S4.6.2	S8.4
m_{cut}	See S4.6.2	kg	0.316	S4.6.2	S8.4
e_f	See S4.6.2	-	0.5	S4.6.2	S8.4
k_f	See S4.6.2	kg^{1-e_f}	1.16×10^{-4}	S4.6.2	S8.4
Community/metacommmunity level					
e_{N_m}	Mass exponent, ISD	-	-2.003	S4.3.3	S4.3.3
$e_{N_{m\infty}}$	Asymptotic mass exponent, IASD	-	-1.49	main text	main text

Table S2. Viscosity of sea water at a range of temperatures. Data were taken from the Chemical Hazards Response Information System of the U.S. Coast Guard (www.chrismanual.com/Intro/prop.htm), and are for "standard" sea water containing 35g salts per kg of solution.

Temp. (°F)	Visc. (Centipoise)	Temp. (°K)	Visc. ($kg \cdot m^{-1} \cdot s^{-1}$)
30	1.88	272.04	0.00188
40	1.61	277.59	0.00161
50	1.40	283.15	0.00140
60	1.21	288.71	0.00121
70	1.06	294.26	0.00106
80	0.92	299.82	0.00092
90	0.82	305.37	0.00082
100	0.73	310.93	0.00073

TABLE S3. List of LMEs. Membership in larger regions and environmental characteristics (temperature, T, net primary productivity, P_{net}, and area) are also listed. N=North, S=South, W=West, E=East, T=tropical, Lat.=latitudinal. The column n is the number of species of non-reef-associated fish listed by FishBase as occurring in the region and having asymptotic mass between 1 and 1000kg. Regions are mapped in figures S7 - S10.

LME name	code	n	T ($°K$)	P_{net} (mg C/m^2 d)	Area (m^2)	Province	Basin	Lat. band
Agulhas Current	30	234	298.89	471.93	2.61E+12	S Africa	Indian Ocean	S
Antarctic	61	75	271.92	696.68	4.17E+12	Antarctic	Antarctic	S
Arabian Sea	32	187	300.68	1030.91	3.90E+12	Indian Ocean	Indian Ocean	T
Baltic Sea	23	107	281.69	1066.35	3.58E+11	Europe	N Atlantic	N
Barents Sea	20	32	276.11	710.15	1.68E+12	Arctic	Arctic	N
Bay of Bengal	34	148	301.85	548.01	3.64E+12	Indian Ocean	Indian Ocean	T
Beaufort Sea	55	48	271.91	1036.24	7.52E+11	Arctic	Arctic	N
Benguela Current	29	394	293.05	1100.39	1.44E+12	S Africa	S Atlantic	S
Black Sea	62	93	288.65	1156.78	4.31E+11	Europe	N Atlantic	N
California Current	3	279	290.36	575.02	2.19E+12	NE Pacific	N Pacific	N
Canary Current	27	556	294.59	940.72	1.10E+12	T E Atlantic	N Atlantic	T
Caribbean Sea	12	342	300.94	409.47	3.23E+12	T W Atlantic	N Atlantic	T
Celtic-Biscay Shelf	24	209	286.29	748.98	7.38E+11	Europe	N Atlantic	N
Chukchi Sea	54	42	273.37	1003.90	5.36E+11	Arctic	Arctic	N
E Bering Sea	1	100	278.18	674.48	1.34E+12	NE Pacific	N Pacific	N
E Brazil Shelf	16	281	300.31	295.08	1.06E+12	T W Atlantic	S Atlantic	T
E China Sea	47	341	295.37	782.40	7.75E+11	NW Pacific	N Pacific	N
E Greenland Shelf	19	77	275.90	464.22	3.09E+11	N Atlantic	N Atlantic	N
E Siberian Sea	56	25	272.09	1199.57	9.00E+11	Arctic	Arctic	N
E-Central Australia	41	311	296.31	383.20	6.39E+11	S Australia	Indian Ocean	S
Faroe Plateau	60	121	283.06	488.54	1.53E+11	Europe	N Atlantic	N
Guinea Current	28	323	300.11	831.35	1.91E+12	T E Atlantic	N Atlantic	T
Gulf of Alaska	2	143	282.56	670.50	1.45E+12	NE Pacific	N Pacific	N
Gulf of California	4	109	298.02	1019.89	2.15E+11	NE Pacific	N Pacific	N
Gulf of Mexico	5	278	299.41	430.91	1.51E+12	T W Atlantic	N Atlantic	T
Gulf of Thailand	35	110	302.26	647.67	3.68E+11	Indian Ocean	Indian Ocean	T
Hudson Bay	63	9	274.44	772.68	8.20E+11	N Atlantic	N Atlantic	N
Humboldt Current	13	268	289.14	846.74	2.53E+12	Cape Horn	S Pacific	S
Iberian Coastal	25	317	290.14	620.51	2.90E+11	Europe	N Atlantic	N
Iceland Shelf	59	108	279.99	634.65	3.08E+11	N Atlantic	N Atlantic	N
Indonesian Sea	38	297	301.94	614.47	2.25E+12	Indian Ocean	Indian Ocean	T
Insular Pacific-Hawaiian	10	152	298.26	214.53	9.77E+11	Hawaii	N Pacific	T
Kara Sea	58	5	272.60	1132.16	7.60E+11	Arctic	Arctic	N
Kuroshio Current	49	260	296.70	423.19	1.33E+12	NW Pacific	N Pacific	N
Laptev Sea	57	26	272.27	1517.95	4.71E+11	Arctic	Arctic	N
Mediterranean Sea	26	321	293.37	418.40	2.46E+12	Europe	N Atlantic	N

LME name	code	n	T (°K)	P_{net} (mg C/m^2 d)	Area (m^2)	Province	Basin	Lat. band
N Australia	39	214	301.56	830.24	7.75E+11	Indian Ocean	Indian Ocean	T
N Brazil Shelf	17	249	300.99	890.74	1.04E+12	T W Atlantic	N Atlantic	T
N Sea	22	123	283.74	937.50	6.62E+11	Europe	N Atlantic	N
NE Australia	40	124	299.68	271.23	1.27E+12	Indian Ocean	Indian Ocean	T
NE U.S. Continental Shelf	7	273	285.47	1194.18	3.13E+11	NW Atlantic	N Atlantic	N
New Zealand Shelf	46	321	288.70	554.86	9.61E+11	S Australia	Indian Ocean	S
Newfoundland-Labrador Shelf	9	103	278.14	758.68	8.58E+11	NW Atlantic	N Atlantic	N
Norwegian Sea	21	148	280.66	545.56	1.11E+12	N Atlantic	N Atlantic	N
NW Australia	45	116	301.17	441.51	9.20E+11	Indian Ocean	Indian Ocean	T
Oyashio Current	51	22	279.74	682.56	5.33E+11	NW Pacific	N Pacific	N
Pacific Central-American	11	270	300.71	703.23	1.96E+12	T E Pacific	N Pacific	T
Patagonian Shelf	14	165	284.23	1082.21	1.14E+12	Cape Horn	S Atlantic	S
Red Sea	33	167	301.32	564.41	4.41E+11	Indian Ocean	Indian Ocean	T
S Brazil Shelf	15	316	296.11	566.91	5.58E+11	Cape Horn	S Atlantic	S
S China Sea	36	607	301.08	464.11	3.14E+12	Indian Ocean	Indian Ocean	T
Scotian Shelf	8	124	280.98	1030.97	2.70E+11	NW Atlantic	N Atlantic	N
SE Australia	42	50	288.47	519.52	1.20E+12	S Australia	Indian Ocean	S
SE U.S. Continental Shelf	6	239	298.72	535.43	3.02E+11	NW Atlantic	N Atlantic	N
Sea of Japan	50	243	286.37	558.97	9.73E+11	NW Pacific	N Pacific	N
Sea of Okhotsk	52	163	277.35	603.55	1.52E+12	NW Pacific	N Pacific	N
Somali Coastal Current	31	96	300.39	744.39	8.34E+11	Indian Ocean	Indian Ocean	T
Sulu-Celebes Sea	37	147	302.03	472.48	9.95E+11	Indian Ocean	Indian Ocean	T
SW Australia	43	141	290.66	458.00	1.05E+12	S Australia	Indian Ocean	S
W Bering Sea	53	128	277.88	541.93	2.01E+12	NW Pacific	N Pacific	N
W Greenland Shelf	18	79	274.42	564.12	3.62E+11	N Atlantic	N Atlantic	N
W-Central Australia	44	140	295.69	409.50	5.52E+11	S Australia	Indian Ocean	S
Yellow Sea	48	337	288.08	1324.96	4.21E+11	NW Pacific	N Pacific	N

Table S4. Province, basin, latitudinal band, and global-region codes and areas. The column n is the number of species of non-reef-associated fish listed by FishBase as occurring in the region and having asymptotic mass between 1 and 1000kg.

Region name	code	n	Area (m^2)
Provinces			
Antarctic	1	75	4.17E+12
Arctic	2	74	5.09E+12
Cape Horn	3	555	4.23E+12
Europe	4	511	5.09E+12
Hawaii	5	152	9.77E+11
Indian Ocean	6	829	1.85E+13
North Atlantic	7	200	2.91E+12
Northeast Pacific	8	393	5.20E+12
Northwest Atlantic	9	415	1.74E+12
Northwest Pacific	10	718	7.56E+12
South Africa	11	536	4.05E+12
South Australia	12	488	4.40E+12
Tropical East Atlantic	13	597	3.02E+12
Tropical East Pacific	14	270	1.96E+12
Tropical West Atlantic	15	516	6.83E+12
Basins			
Antarctic	1	75	4.17E+12
Arctic	2	74	5.09E+12
Indian Ocean	3	1216	2.55E+13
North Atlantic	4	1075	1.85E+13
North Pacific	5	1062	1.57E+13
South Atlantic	6	679	4.20E+12
South Pacific	7	268	2.53E+12
Latitudinal bands			
North	1	1542	2.76E+13
South	2	1253	1.69E+13
Tropical	3	1808	3.13E+13
Global continental shelf seas			
Global region	1	2885	7.58E+13

Table S5. Truncated Pareto fit results for the regions deemed linear. $-b$ is the diversity spectrum slope. Diversity spectrum slopes of LMEs are mapped in the main text. Upper and lower 95% and 99% confidence intervals are also given for the truncated Pareto fit. The column var(b) is variance of the estimates of b for the resamplings (see section 5 of the main text).

Region name	b, lower 99%	b, lower 95%	b	b, upper 95%	b, upper 99%	var(b)
LMEs						
Agulhas Current	0.207	0.224	0.291	0.359	0.386	0.0012
Antarctic	0.565	0.595	0.731	0.904	0.942	0.0061
Arabian Sea	0.140	0.169	0.237	0.312	0.342	0.0015
Barents Sea	0.172	0.219	0.364	0.539	0.618	0.0068
Bay of Bengal	0.136	0.151	0.234	0.319	0.348	0.0018
Beaufort Sea	0.311	0.352	0.486	0.658	0.687	0.0059
Benguela Current	0.313	0.325	0.378	0.430	0.445	0.0007
Black Sea	0.161	0.188	0.288	0.398	0.429	0.0029
California Current	0.282	0.305	0.370	0.434	0.460	0.0012
Canary Current	0.361	0.372	0.418	0.467	0.489	0.0006
Caribbean Sea	0.384	0.404	0.464	0.532	0.549	0.0011
Celtic-Biscay Shelf	0.267	0.286	0.357	0.429	0.460	0.0013
Chukchi Sea	0.329	0.385	0.551	0.748	0.815	0.0089
East-Central Australia	0.301	0.321	0.383	0.451	0.469	0.0011
East Brazil Shelf	0.322	0.342	0.407	0.477	0.494	0.0013
East China Sea	0.389	0.411	0.472	0.538	0.550	0.0010
East Greenland Shelf	0.293	0.318	0.417	0.536	0.575	0.0030

Region name	b, lower 99%	b, lower 95%	b	b, upper 95%	b, upper 99%	var(b)
East Bering Sea	0.436	0.453	0.560	0.686	0.740	0.0036
Guinea Current	0.297	0.316	0.375	0.437	0.457	0.0010
Gulf of Alaska	0.379	0.389	0.485	0.590	0.631	0.0026
Gulf of California	0.334	0.354	0.458	0.571	0.612	0.0031
Gulf of Mexico	0.353	0.376	0.445	0.518	0.543	0.0014
Gulf of Thailand	0.132	0.158	0.256	0.355	0.387	0.0025
Humboldt Current	0.337	0.373	0.437	0.505	0.532	0.0013
Iberian Coastal	0.254	0.267	0.322	0.373	0.397	0.0008
Indonesian Sea	0.300	0.318	0.383	0.448	0.486	0.0012
Insular Pacific-Hawaiian	0.150	0.175	0.258	0.359	0.383	0.0021
Kuroshio Current	0.240	0.251	0.320	0.390	0.418	0.0013
Mediterranean Sea	0.235	0.252	0.305	0.363	0.381	0.0008
New Zealand Shelf	0.293	0.301	0.363	0.422	0.440	0.0010
Newfoundland-Labrador Shelf	0.217	0.239	0.329	0.434	0.465	0.0023
North Australia	0.235	0.264	0.334	0.416	0.450	0.0015
North Brazil Shelf	0.291	0.316	0.386	0.461	0.480	0.0014
North Sea	0.163	0.189	0.269	0.360	0.384	0.0018
Northeast Australia	0.339	0.376	0.469	0.583	0.622	0.0031
Northeast U.S. Continental Shelf	0.249	0.273	0.332	0.400	0.420	0.0010
Northwest Australia	0.321	0.340	0.449	0.573	0.586	0.0034
Pacific Central-American	0.315	0.342	0.405	0.485	0.506	0.0014
Patagonian Shelf	0.281	0.318	0.393	0.482	0.507	0.0020
Red Sea	0.226	0.256	0.338	0.432	0.454	0.0020
Scotian Shelf	0.208	0.223	0.310	0.405	0.435	0.0021
Sea of Japan	0.366	0.381	0.458	0.544	0.583	0.0016
Sea of Okhotsk	0.513	0.547	0.639	0.750	0.793	0.0031
Somali Coastal Current	0.067	0.096	0.195	0.301	0.315	0.0027
South Brazil Shelf	0.339	0.357	0.421	0.487	0.506	0.0011
South China Sea	0.401	0.415	0.463	0.516	0.526	0.0007
Southeast Australia	0.319	0.363	0.535	0.750	0.843	0.0100
Southeast U.S. Continental Shelf	0.288	0.312	0.391	0.473	0.489	0.0016
Southwest Australia	0.164	0.199	0.291	0.392	0.424	0.0025
Sulu-Celebes Sea	0.172	0.186	0.276	0.372	0.400	0.0024
West Bering Sea	0.476	0.509	0.611	0.719	0.757	0.0031
West-Central Australia	0.173	0.201	0.290	0.391	0.424	0.0023
Yellow Sea	0.323	0.345	0.403	0.472	0.498	0.0011
Provinces						
Antarctic	0.549	0.590	0.731	0.895	0.963	0.0063
Arctic	0.298	0.334	0.440	0.561	0.610	0.0038
Cape Horn	0.402	0.415	0.459	0.511	0.529	0.0006
Europe	0.292	0.306	0.349	0.396	0.415	0.0006
Hawaii	0.142	0.169	0.258	0.350	0.387	0.0021
Indian Ocean	0.430	0.434	0.477	0.522	0.535	0.0005
Northeast Pacific	0.376	0.392	0.449	0.513	0.528	0.0009
Northwest Atlantic	0.343	0.359	0.413	0.470	0.487	0.0008
Northwest Pacific	0.444	0.460	0.503	0.553	0.576	0.0006
South Africa	0.321	0.332	0.378	0.425	0.437	0.0005
South Australia	0.368	0.378	0.425	0.475	0.486	0.0006
Tropical East Atlantic	0.360	0.377	0.420	0.468	0.483	0.0005
Tropical East Pacific	0.313	0.334	0.405	0.482	0.511	0.0014
Tropical West Atlantic	0.415	0.429	0.479	0.534	0.545	0.0007
Basins						
Antarctic	0.570	0.591	0.731	0.903	0.965	0.0061
Arctic	0.294	0.321	0.440	0.567	0.595	0.0038
Indian Ocean	0.463	0.470	0.504	0.539	0.550	0.0003
North Atlantic	0.430	0.439	0.472	0.510	0.517	0.0003
North Pacific	0.490	0.502	0.540	0.582	0.590	0.0004
South Pacific	0.359	0.368	0.437	0.504	0.542	0.0013
Latitudinal bands						
N	0.463	0.474	0.503	0.536	0.545	0.0002

Region name	b, lower 99%	b, lower 95%	b	b, upper 95%	b, upper 99%	var(b)
S	0.452	0.461	0.493	0.523	0.534	0.0003
T	0.489	0.498	0.525	0.554	0.563	0.0002
Global continental shelf seas						
Global	0.532	0.536	0.561	0.585	0.590	0.0001
Global, with mammals	0.494	0.498	0.521	0.544	0.551	0.0001

Table S6. Statistics for regions for which the tP was statistically rejected. Column 2 has the p-value for the composite test that data came from a tP distribution with unknown exponent and truncation points 1 and 1000kg. The last column is for the likelihood ratio test comparing the fit of the tP and qtP distributions. See section 5 of the main text for details of the tests.

System name	tP test, p-value	spectrum linearity statistic	log lik. tP	log lik. qtP	lik. rat. test p-value
LMEs					
Baltic Sea	3.90E-03	0.9544	-399.48	-395.52	4.89E-03
Benguela Current	4.00E-04	0.9825	-1492.94	-1486.79	4.51E-04
Canary Current	2.00E-04	0.9930	-2016.89	-2013.08	5.80E-03
Faroe Plateau	7.00E-04	0.9374	-505.01	-495.69	<1.00E-04
Iberian Coastal	2.00E-04	0.9881	-1273.53	-1269.65	5.36E-03
Iceland Shelf	7.00E-04	0.9175	-455.39	-444.75	<1.00E-04
Mediterranean Sea	2.00E-04	0.9902	-1312.02	-1308.42	7.32E-03
Norwegian Sea	1.50E-03	0.9628	-610.22	-603.93	3.89E-04
South China Sea	2.70E-03	0.9949	-2098.39	-2098.06	4.15E-01
West Greenland Shelf	4.00E-04	0.8592	-307.17	-296.69	<1.00E-04
Provinces					
Cape Horn	3.00E-03	0.9939	-1926.67	-1923.90	1.86E-02
Europe	<1.00E-04	0.9887	-1995.05	-1989.26	6.62E-04
North Atlantic	2.60E-03	0.9570	-790.48	-781.50	<1.00E-04
South Africa	3.00E-04	0.9858	-2029.84	-2022.70	1.56E-04
Tropical East Atlantic	4.00E-04	0.9930	-2161.86	-2157.90	4.90E-03
Basins					
Indian Ocean	4.00E-04	0.9949	-4021.41	-4019.25	3.74E-02
North Atlantic	<1.00E-04	0.9900	-3681.12	-3671.61	<1.00E-04
South Atlantic	<1.00E-04	0.9782	-2417.71	-2403.36	<1.00E-04
Latitudinal bands					
N	<1.00E-04	0.9925	-5104.71	-5097.16	1.01E-04
S	<1.00E-04	0.9806	-4195.00	-4174.80	<1.00E-04
T	<1.00E-04	0.9927	-5845.59	-5837.89	<1.00E-04
Global continental shelf seas					
Global	<1.00E-04	0.9900	-8977.65	-8958.43	<1.00E-04
Global, with mammals	<1.00E-04	0.9953	-9631.51	-9623.30	<1.00E-04
